# Associations of body shape index (ABSI) and hip index with liver, metabolic, and inflammatory biomarkers in the UK Biobank cohort

**DOI:** 10.1038/s41598-022-12284-4

**Published:** 2022-05-25

**Authors:** Sofia Christakoudi, Elio Riboli, Evangelos Evangelou, Konstantinos K. Tsilidis

**Affiliations:** 1 Department of Epidemiology and Biostatistics, School of Public Health, Imperial College London, St Mary’s Campus, Norfolk place, London, W2 1PG UK; 2 Department of Inflammation Biology, School of Immunology and Microbial Sciences, King’s College London, London, UK; 3 Department of Hygiene and Epidemiology, University of Ioannina School of Medicine, Ioannina, Greece

**Keywords:** Type 2 diabetes, Dyslipidaemias, Metabolic syndrome, Obesity, Non-alcoholic fatty liver disease

## Abstract

Associations of liver, metabolic, and inflammatory biomarkers in blood with body shape are unclear, because waist circumference (WC) and hip circumference (HC) are dependent on overall body size, resulting in bias. We have used the allometric “a body shape index” (ABSI = WC_(mm)_$\,*\,$Weight_(kg)_^-2/3^$\,*\,$Height_(m)_^5/6^) and hip index (HI_women_ = HC_(cm)_$\,*\,$Weight_(kg)_^-0.482^$\,*\,$Height_(cm)_^0.310^, HI_men_ = HC_(cm)_$\,*\,$Weight_(kg)_^-2/5^$\,*\,$Height_(cm)_^1/5^), which are independent of body mass index (BMI) by design, in multivariable linear regression models for 121,879 UK Biobank men and 135,559 women. Glucose, glycated haemoglobin (HbA1c), triglycerides, low-density-lipoprotein cholesterol, apolipoprotein-B, alanine aminotransferase (ALT), gamma-glutamyltransferase, and lymphocytes were associated positively with BMI and ABSI but inversely with HI. High-density-lipoprotein cholesterol and apolipoprotein-A1 were associated inversely with BMI and ABSI but positively with HI. Lipid-related biomarkers and ALT were associated only with HI in obese men. C-reactive protein, neutrophils, monocytes, and alkaline phosphatase were associated positively, while bilirubin was associated inversely, with BMI and ABSI but not with HI. Associations were consistent within the clinical reference ranges but were lost or changed direction for low or high biomarker levels. Our study confirms associations with waist and hip size, independent of BMI, for metabolic biomarkers but only with waist size for inflammatory biomarkers, suggesting different contribution of the mechanistic pathways related to body shape.

## Introduction

Obesity is associated with type 2 diabetes, cardiovascular diseases, and chronic low-grade inflammation^1,2^. Obesity is additionally associated with non-alcoholic fatty liver disease (NAFLD)^3^, and the histological evidence for liver damage is dramatically reduced after bariatric surgery, alongside a reduction of body mass index (BMI)^4^. Nevertheless, diabetes and NAFLD can also be present in non-obese individuals^5,6^, and it remains unclear whether any improvements after bariatric surgery are a direct effect of fat reduction or the result of other metabolic and hormonal changes. Notably, the complications of obesity are dependent not simply on fat quantity but also on the location of fat accumulation, with abdominal and more specifically visceral fat contributing to a higher risk and gluteofemoral fat showing an apparently protective effect^7,8^, supporting the involvement of alternative mechanistic factors beyond fat excess. Studies evaluating associations with body shape, however, have traditionally used waist and hip circumference^9^, which are not only strongly correlated with each other, but as absolute measures of regional body size are dependent on overall body size reflected in BMI. Consequently, statistical models including simultaneously waist and hip circumference and BMI with the aim of mutual adjustment provide biased risk estimates, overestimating both the positive and the inverse associations^10,11^.

Conversely, “a body shape index” (ABSI) and hip index (HI) are allometric anthropometric indices independent of BMI by design and compare waist and hip size among individuals with the same weight and height^12,13^. Body shape phenotypes defined with ABSI and HI also provide information for body composition, with phenotypes with discordant ABSI and HI showing the largest difference in visceral adipose tissue (VAT) (lowest for small-ABSI-large-HI, “pear”, highest for large-ABSI-small-HI, “apple” phenotype) and, in women, also the largest difference in gynoid fat mass (lowest for “apple”, highest for “pear” phenotype)^14^. Associations of ABSI and HI with clinical outcomes such as cardio-metabolic diseases, cancer, and mortality have previously been evaluated^10,13,15,16^, but their associations with metabolic and inflammatory biomarkers have received little attention. Examining associations with clinically relevant biomarkers is important because biomarker dichotomisation in order to define a clinical outcome such as diabetes or dyslipidaemia overlooks the continuity in their spectrum and precludes the identification of associations within the biomarker reference ranges, which may provide mechanistic insights.

Therefore, using data from the UK Biobank cohort, we have examined the associations of body shape phenotypes based on ABSI and HI with blood levels of liver function tests and metabolic and inflammatory biomarkers and have explored the linearity of the associations across biomarker levels and their heterogeneity according to body size, medication use, and alcohol consumption.

## Methods

### Study population

UK Biobank is a population-based cohort, including half a million individuals registered with the National Health Service of the of the United Kingdom and living within 40 km of the assessment centres in England, Scotland, and Wales, which were recruited between 2006 and 2010 at age 40 to 70 years^17^. Consistent with our previous reports^10,14^, we limited this study to participants with self-reported white ancestry, as there were insufficient numbers from other ethnicities. We also excluded participants, if their anthropometric measurements were missing or extreme (height < 130 cm, waist circumference < 50 or > 160 cm, BMI < 18.5 or ≥ 45 kg/m^2^, as these BMI groups were small but could have large leverage on the results), or if their genetically-determined sex did not match the self-reported sex, or if they were pregnant at enrolment. To reduce the potential influence of reverse causality, we further excluded participants, if they had prevalent cancer at enrolment (defined as in^10^), if they developed cancer or died within two years after enrolment, if they had reported diabetes mellitus (including type 1 and type 2) at enrolment, or if they had reported thyroid, pituitary, adrenal, or other endocrine non-cancer illness, or inflammatory bowel disease, or liver disease, or kidney failure, or chronic respiratory disease, or heart failure (see lists of illnesses in the legend of Supplementary Figure S1), or if they were receiving lipid lowering drugs or exogenous glucocorticoids at enrolment (listed in Supplementary Table S1), or anti-hypertensive drugs, or hormone replacement therapy (HRT), or oral contraceptives (as these can affect some metabolic biomarkers, see definition of variables in Supplementary Methods). Finally, we excluded participants with missing all biomarker measurements (ensuring that each participant included in the study contributes to at least one biomarker). In total, we excluded 244,974 participants (48.8%, Supplementary Figure S1).

### Body-shape indices

Anthropometric measurements were obtained by trained UK Biobank technicians, at the natural indent or the umbilicus for waist circumference, or at the widest point for hip circumference^18^. We calculated ABSI for both sexes and HI for women with coefficients from the National Health and Nutrition Examination Survey (NHANES)^12,13^. For HI in men, we used coefficients based on UK Biobank data, to avoid the inverse correlation between HI and BMI introduced by the coefficients from NHANES^14^:

$${\text{ABSI }} = {\text{ WC}}\left( {{\text{mm}}} \right)*{\text{Weight}}\left( {{\text{kg}}} \right)^{{ - {2}/{3}}} *{\text{Height}}\left( {\text{m}} \right)^{{{5}/{6}}}$$

$${\text{HI}}_{{{\text{women}}}} = {\text{ HC}}\left( {{\text{cm}}} \right)*{\text{Weight}}\left( {{\text{kg}}} \right)^{{ - 0.{482}}} *{\text{Height}}\left( {{\text{cm}}} \right)^{{0.{31}0}}$$

$${\text{HI}}_{{{\text{men}}}} = {\text{ HC}}\left( {{\text{cm}}} \right)*{\text{Weight}}\left( {{\text{kg}}} \right)^{{ - {2}/{5}}} *{\text{Height}}\left( {{\text{cm}}} \right)^{{{1}/{5}}}$$

$${\text{BMI }} = {\text{ Weight}}\left( {{\text{kg}}} \right)*{\text{Height}}\left( {\text{m}} \right)^{{ - {2}}}$$

We standardised anthropometric indices to sex-specific z-scores when using them on a continuous scale (value minus mean, divided by standard deviation, SD). We defined body shape phenotypes with dichotomised ABSI (≥ 80 for men, ≥ 73 for women) and HI (≥ 49 for men, ≥ 64 for women), as in our previous study^14^: “pear” – small-ABSI-large-HI (reference), “slim”—small-ABSI-small-HI, “wide” – large-ABSI-large-HI, “apple”—large-ABSI-small-HI^14^. We defined BMI categories according to the World Health Organisation criteria: normal weight (BMI ≥ 18.5 to < 25 kg/m^2^, reference), overweight (BMI ≥ 25 to < 30 kg/m^2^), obese (BMI ≥ 30 to < 45 kg/m^2^) and additionally defined combined categories by ABSI, HI, and BMI (“pear” normal weight reference).

### Biomarker measurements

Blood samples in UK Biobank were obtained throughout the day (8 am to 9 pm), with no specific requirements for fasting. Serum levels of biomarkers were measured on a Beckman Coulter AU5800 analyser. As liver function tests, we examined bilirubin (total and direct, measured with colorimetric assays), and liver enzymes (measured with enzymatic rate assays): aspartate aminotransferase (AST), alanine aminotransferase (ALT), gamma glutamyltransferase (GGT), and alkaline phosphatase (ALP). As biomarkers of lipid metabolism, we examined high-density lipoprotein cholesterol (HDL-C, enzyme immune-inhibition assay), low-density lipoprotein cholesterol (LDL-C, enzymatic selective protection assay), triglycerides (enzymatic assay), apolipoproteins A1 and B (ApoA1, ApoB, immuno-turbidimetric assays). As biomarkers of glucose metabolism, we examined serum glucose (enzymatic assay) and HbA1c (measured in red blood cells with high-performance liquid chromatography on Bio-Rad VARIANT II Turbo analyser), which is not affected by fasting and provides information for glucose status over the last three months. As inflammatory biomarkers, we examined white blood cell counts (lymphocytes, monocytes, neutrophils) and serum levels of C-reactive protein (CRP, high sensitivity immuno-turbidimetric assay).

Direct bilirubin was below the limit of detection for 7.0% of men and 21.8% of women and, for these, we used quantile regression imputation of truncated left-censored data (QRILC), which estimates the parameters of the distribution from the available data (**imputeLCMD** v2.0 package in R). For the remaining biomarkers, values outside the limits of detection were few (< 0.5%) and were assigned, correspondingly, to half the lowest detected level or to the highest detected level (Supplementary Table S2).

We log-transformed all biomarker measurements, to mitigate the influence of right-skewed distributions, and calculated sex-specific z-scores, to provide a standardised scale for comparability.

### Statistical analysis

To examine associations of biomarkers with body shape, we calculated SD differences (95% confidence intervals) with multivariable linear regression models. To examine independent associations with waist and hip size, for each biomarker as an outcome, we used an additive model including ABSI, HI, and BMI on a continuous scale (sex-specific z-scores), and covariates, interpreting the estimates as SD difference in biomarker levels per one SD increment of the anthropometric index. To examine associations with body shape phenotypes, we used a model including the ABSI-by-HI cross-classification (“pear” reference, “slim”, “wide”, “apple”), BMI categories, and covariates. To examine heterogeneity by body size, we used categories combining body size and body shape in the BMI-by-ABSI-by-HI cross-classification, which is equivalent to an interaction model. As in our previous study^10^, covariates evaluated at enrolment and used for adjustment of all models comprised height, age, weight change within the last year preceding enrolment, smoking status, alcohol consumption, physical activity, Townsend deprivation index (tertiles), region of the assessment centre, and additionally time of blood collection, fasting time, use of nonsteroidal anti-inflammatory drugs (NSAID, as these may affect inflammatory factors), paracetamol use (as this may affect liver function tests), and in women also menopausal status, oral contraceptives use (never, past), HRT use (never, past), and age at the last live birth. Covariates were mainly defined as previously^10^, with the study specific definitions explained in Supplementary Methods. We replaced missing values for covariates with the median sex-specific value or category, as missing information was limited (Supplementary Table S2).

We examined the consistency of the associations with BMI, ABSI, or HI (on a continuous scale) across the available range of biomarker levels, using as a smoothing function generalised additive models with restricted maximum likelihood (REML) estimation (package mgcv v1.8–39 implemented in ggplot2 v.3.3.5 in R). For each of the anthropometric indices we used residuals from multivariable linear regression models including the other two anthropometric indices and covariates. We further examined non-linearity by including in fully adjusted models restricted cubic splines for one of the anthropometric indices on a continuous scale (using function rcs from package rms v.6.2–0 in R, with knots at -2, 0, and 2 for sex-specific z-scores) and the other two anthropometric indices on a linear scale. For biomarkers with plots suggesting change of direction in the associations towards the tails of the distributions, we additionally calculated SD differences with fully adjusted linear models for subgroups according to biomarker levels. As biomarkers reach clinically relevant levels towards the tails of their distributions, examining the central part of the distribution acts as a sensitivity analysis, excluding any underlying medical conditions contributing to the tails.

To evaluate the statistical significance of individual terms, we used Wald tests. To evaluate the contribution of body shape phenotypes overall, we used a likelihood ratio test, comparing a model including BMI categories and covariates with a model additionally including an ABSI-by-HI cross-classification. To evaluate heterogeneity by BMI, we used a likelihood ratio test, comparing the additive model including the ABSI-by-HI cross-classification, BMI categories and covariates with the interaction model including the BMI-by-ABSI-by-HI cross-classification and covariates. To evaluate non-linearity, we used a likelihood ratio test, comparing the additive model including BMI, ABSI, and HI on a continuous scale and covariates with the model replacing the linear term for one of the anthropometric indices with the corresponding restricted cubic splines. Tests for statistical significance were two-sided, considering *p* < 0.0001 as evidence for association (equivalent to a Bonferroni correction for 500 comparisons) and *p* < 1*10^–6^ as a strong evidence for association (equivalent to a Bonferroni correction for 50,000 comparisons).

To examine associations between biomarkers, we calculated partial Pearson correlation coefficients with adjustment for ABSI, HI, BMI (continuous, z-scores), and covariates as above (except for region of the assessment centre and age at the last live birth).

In sensitivity analyses, we examined the influence of covariates overall in unadjusted models (including only BMI, ABSI, and HI), the influence of recent weight change (excluding participants reporting weight loss or weight gain within the year preceding enrolment), and the influence of medication use and alcohol consumption, examining subgroups with no NSAID or paracetamol use, as well as subgroups with NSAID use, paracetamol use, and different quantities of alcohol consumption. All subgroup analyses were based on fully adjusted models, omitting the corresponding variable, which defined the subgroups.

We used R version 4.1.3 for all analyses^19^.

### Ethical approval and consent to participate

This research was conducted according to the principles expressed in the Declaration of Helsinki. The UK Biobank cohort has been approved by the North West Multicenter Research Ethics Committee, UK (Ref: 16/NW/0274). Written informed consent has been obtained from all study participants. The current study was approved by the UK Biobank access management board. Participants who had withdrawn consent by the time of the analysis were excluded from dataset.

## Results

### Cohort characteristics

The study included 121,879 men and 135,559 women. Consistent with our previous study^14^, participants with large ABSI (“apple” and “wide” phenotypes) were older and with less healthy lifestyle compared to participants with small ABSI (“pear” and “slim” phenotypes) (Table 1, Supplementary Table S3). The proportions of participants with NSAID or paracetamol use were comparable (Table 1).

**Table 1:** Anthropometric characteristics of study participants and biomarker levels.

MEN	Overall	Pear	Slim	Wide	Apple
Cohort: n (% per sex)	121,879	32,736 (26.9)	37,383 (30.7)	32,504 (26.7)	19,256 (15.8)
Age (years) ^**a**^	55.1 (8.1)	53.8 (8.2)	53.6 (7.9)	57.1 (7.9)	56.6 (7.7)
Anthropometric indices ^**a**^					
BMI (kg/m^2^)	27.1 (3.7)	26.8 (3.8)	27.2 (3.5)	27.1 (3.9)	27.6 (3.6)
ABSI	79.2 (4.0)	76.9 (2.4)	76.1 (2.8)	83.0 (2.4)	82.7 (2.2)
HI	49.1 (1.6)	50.1 (0.9)	47.7 (1.1)	50.4 (1.1)	47.9 (0.9)
Liver function tests ^**b**^					
Bilirubin total ($\upmu$mol/L)	9.52 (4.51–20.1)	9.79 (4.59–20.9)	9.60 (4.53–20.4)	9.36 (4.48–19.6)	9.17 (4.42–19.1)
Bilirubin direct ($\upmu$mol/L)	1.72 (0.78–3.79)	1.78 (0.81–3.93)	1.73 (0.78–3.82)	1.69 (0.78–3.70)	1.65 (0.76–3.60)
ALP (U/L)	77.9 (47.5–128)	76.2 (46.7–124)	77.0 (47.2–126)	79.4 (48.3–130)	80.3 (48.6–133)
GGT (U/L)	33.9 (10.9–106)	31.2 (10.4–93.0)	33.6 (11.1–102)	34.9 (11.0–111)	38.0 (11.7–123)
AST (U/L)	26.5 (15.8–44.5)	26.3 (16.0–43.4)	26.8 (16.1–44.7)	26.3 (15.6–44.4)	26.8 (15.6–46.2)
ALT (U/L)	24.1 (10.3–56.5)	23.0 (10.1–52.4)	24.5 (10.5–57.3)	24.0 (10.2–56.5)	25.7 (10.8–61.2)
Metabolic biomarkers ^**b**^					
HDL-C (mmol/L)	1.28 (0.82–1.99)	1.32 (0.85–2.04)	1.27 (0.82–1.98)	1.28 (0.82–1.99)	1.24 (0.80–1.93)
Apolipoprotein A1 (g/L)	1.42 (1.05–1.93)	1.44 (1.07–1.94)	1.42 (1.05–1.92)	1.43 (1.05–1.93)	1.40 (1.03–1.91)
LDL-C (mmol/L)	3.67 (2.42–5.56)	3.59 (2.36–5.46)	3.66 (2.41–5.56)	3.70 (2.45–5.59)	3.74 (2.48–5.64)
Apolipoprotein B (g/L)	1.06 (0.69–1.64)	1.03 (0.67–1.59)	1.06 (0.69–1.64)	1.07 (0.69–1.65)	1.09 (0.71–1.67)
Triglycerides (mmol/L)	1.69 (0.60–4.77)	1.53 (0.55–4.28)	1.70 (0.59–4.89)	1.72 (0.63–4.69)	1.91 (0.69–5.30)
HbA1c (mmol/mol)	34.3 (27.0–43.7)	33.9 (27.2–42.3)	34.2 (27.0–43.3)	34.6 (27.0–44.4)	35.0 (26.8–45.5)
Glucose (mmol/L)	4.90 (3.74–6.44)	4.85 (3.77–6.25)	4.91 (3.77–6.39)	4.91 (3.73–6.48)	4.97 (3.66–6.75)
Inflammatory biomarkers ^**b**^					
Lymphocytes (10^9^/L)	1.77 (0.97–3.23)	1.74 (0.96–3.14)	1.77 (0.98–3.20)	1.78 (0.96–3.29)	1.82 (1.00–3.33)
Monocytes (10^9^/L)	0.47 (0.24–0.93)	0.45 (0.23–0.89)	0.46 (0.24–0.91)	0.48 (0.23–0.97)	0.49 (0.25–0.97)
Neutrophils (10^9^/L)	3.90 (2.05–7.39)	3.76 (1.99–7.10)	3.83 (2.02–7.29)	3.99 (2.12–7.52)	4.09 (2.16–7.76)
CRP (mg/L)	1.21 (0.17–8.66)	1.02 (0.14- 7.35)	1.13 (0.16- 7.84)	1.36 (0.19- 9.59)	1.52 (0.23–10.2)
Subgroups: n (%)					
NO weight change	80,074 (65.7)	22,057 (67.4)	24,542 (65.7)	21,353 (65.7)	12,122 (63.0)
NO NSAID or paracetamol	87,379 (71.7)	24,124 (73.7)	26,895 (71.9)	22,969 (70.7)	13,391 (69.5)
NSAID use	23,126 (19.0)	5878 (18.0)	7218 (19.3)	6220 (19.1)	3810 (19.8)
Paracetamol use	20,009 (16.4)	4935 (15.1)	5992 (16.0)	5604 (17.2)	3478 (18.1)

Women	Overall	Pear	Slim	Wide	Apple
Cohort: n (% per sex)	135,559	35,985 (26.5)	31,054 (22.9)	39,632 (29.2)	28,888 (21.3)
Age (years)^**a**^	55.5 (7.9)	54.6 (8.0)	54.1 (7.8)	56.9 (7.8)	56.2 (7.8)
Anthropometric indices^**a**^					
BMI (kg/m^2^)	26.2 (4.4)	25.8 (4.5)	25.8 (4.1)	26.6 (4.8)	26.8 (4.1)
ABSI	73.3 (4.8)	69.7 (2.4)	69.3 (2.8)	77.2 (3.4)	77.0 (3.2)
HI	64.3 (2.4)	65.9 (1.4)	62.1 (1.6)	66.0 (1.6)	62.3 (1.4)
Liver function tests ^**b**^					
Bilirubin total ($\upmu$mol/L)	7.63 (3.72–15.6)	7.88 (3.81–16.3)	7.78 (3.76–16.1)	7.46 (3.68–15.1)	7.42 (3.67–15.0)
Bilirubin direct ($\upmu$mol/L)	1.32 (0.57–3.06)	1.39 (0.60–3.24)	1.36 (0.58–3.17)	1.28 (0.56–2.92)	1.26 (0.55–2.85)
ALP (U/L)	79.6 (45.3–140)	77.1 (43.7–136)	76.0 (43.0–134)	82.9 (47.8–144)	82.2 (47.7–141)
GGT (U/L)	22.2 (7.5–65.4)	20.2 (7.3–55.7)	20.9 (7.5–58.6)	23.3 (7.8–69.8)	24.9 (8.0–77.3)
AST (U/L)	23.0 (14.1–37.4)	22.6 (14.2–35.8)	22.8 (14.0–36.9)	23.2 (14.3–37.7)	23.5 (14.0–39.5)
ALT (U/L)	17.5 (7.8–39.1)	16.3 (7.7–34.2)	16.9 (7.7–37.0)	18.0 (8.0–40.2)	19.1 (8.1–44.9)
Metabolic biomarkers^**b**^					
HDL-C (mmol/L)	1.59 (1.03–2.47)	1.67 (1.10–2.55)	1.61 (1.04–2.49)	1.58 (1.02–2.44)	1.50 (0.96–2.34)
Apolipoprotein A1 (g/L)	1.63 (1.19–2.22)	1.66 (1.22–2.26)	1.63 (1.20–2.23)	1.63 (1.19–2.22)	1.58 (1.15–2.17)
LDL-C (mmol/L)	3.64 (2.32–5.71)	3.49 (2.24–5.42)	3.54 (2.25–5.58)	3.73 (2.41–5.78)	3.82 (2.46–5.96)
Apolipoprotein B (g/L)	1.03 (0.65–1.63)	0.98 (0.63–1.53)	1.00 (0.63–1.59)	1.05 (0.67–1.65)	1.09 (0.69–1.72)
Triglycerides (mmol/L)	1.29 (0.51–3.27)	1.11 (0.49–2.53)	1.20 (0.49–2.95)	1.38 (0.56–3.41)	1.55 (0.60–4.04)
HbA1c (mmol/mol)	34.4 (27.6–43.0)	33.9 (27.5–41.8)	34.1 (27.5–42.3)	34.8 (27.8–43.4)	35.1 (27.7–44.3)
Glucose (mmol/L)	4.90 (3.82–6.28)	4.85 (3.82–6.15)	4.88 (3.84–6.19)	4.92 (3.82–6.34)	4.97 (3.82–6.46)
Inflammatory biomarkers^**b**^					
Lymphocytes (10^9^/L)	1.88 (1.05–3.38)	1.82 (1.03–3.23)	1.86 (1.05–3.30)	1.90 (1.05–3.45)	1.96 (1.09–3.52)
Monocytes (10^9^/L)	0.40 (0.19–0.84)	0.40 (0.19–0.82)	0.40 (0.20–0.82)	0.41 (0.20–0.85)	0.41 (0.20–0.86)
Neutrophils (10^9^/L)	3.89 (2.05–7.35)	3.80 (2.03–7.13)	3.82 (2.00–7.29)	3.95 (2.10–7.41)	3.97 (2.09–7.57)
CRP (mg/L)	1.19 (0.15–9.36)	1.00 (0.13–7.55)	1.00 (0.13–7.56)	1.37 (0.17–10.9)	1.45 (0.19–10.9)
Subgroups: n (%)					
NO weight change	73,760 (54.4)	20,292 (56.4)	17,252 (55.6)	21,229 (53.6)	14,987 (51.9)
NO NSAID or paracetamol	84,614 (62.4)	22,724 (63.1)	19,609 (63.1)	24,433 (61.6)	17,848 (61.8)
NSAID use	30,504 (22.5)	8124 (22.6)	6990 (22.5)	8853 (22.3)	6537 (22.6)
Paracetamol use	34,410 (25.4)	8858 (24.6)	7640 (24.6)	10,444 (26.4)	7468 (25.9)

ABSI – a body shape index (cut-offs ≥ 80 for men, ≥ 73 for women); ALP – alkaline phosphatase; ALT – alanine aminotransferase; Apple – large-ABSI-small-HI; AST – aspartate aminotransferase; BMI – body mass index; CRP – C-reactive protein; GGT – gamma-glutamyltransferase; HbA1c – haemoglobin A1c (glycated haemoglobin); HDL-C – high-density lipoprotein cholesterol; HI – hip index (cut-offs ≥ 49 for men, ≥ 64 for women); LDL-C – low-density lipoprotein cholesterol; NSAID – nonsteroidal anti-inflammatory drugs; Pear – small-ABSI-large-HI; Slim – small-ABSI-small-HI; Wide – large-ABSI-large-HI; ^a^ – mean (standard deviation); ^b^ – geometric mean (reference range).

### Associations of biomarkers with body size and body shape

**Figure 1:**
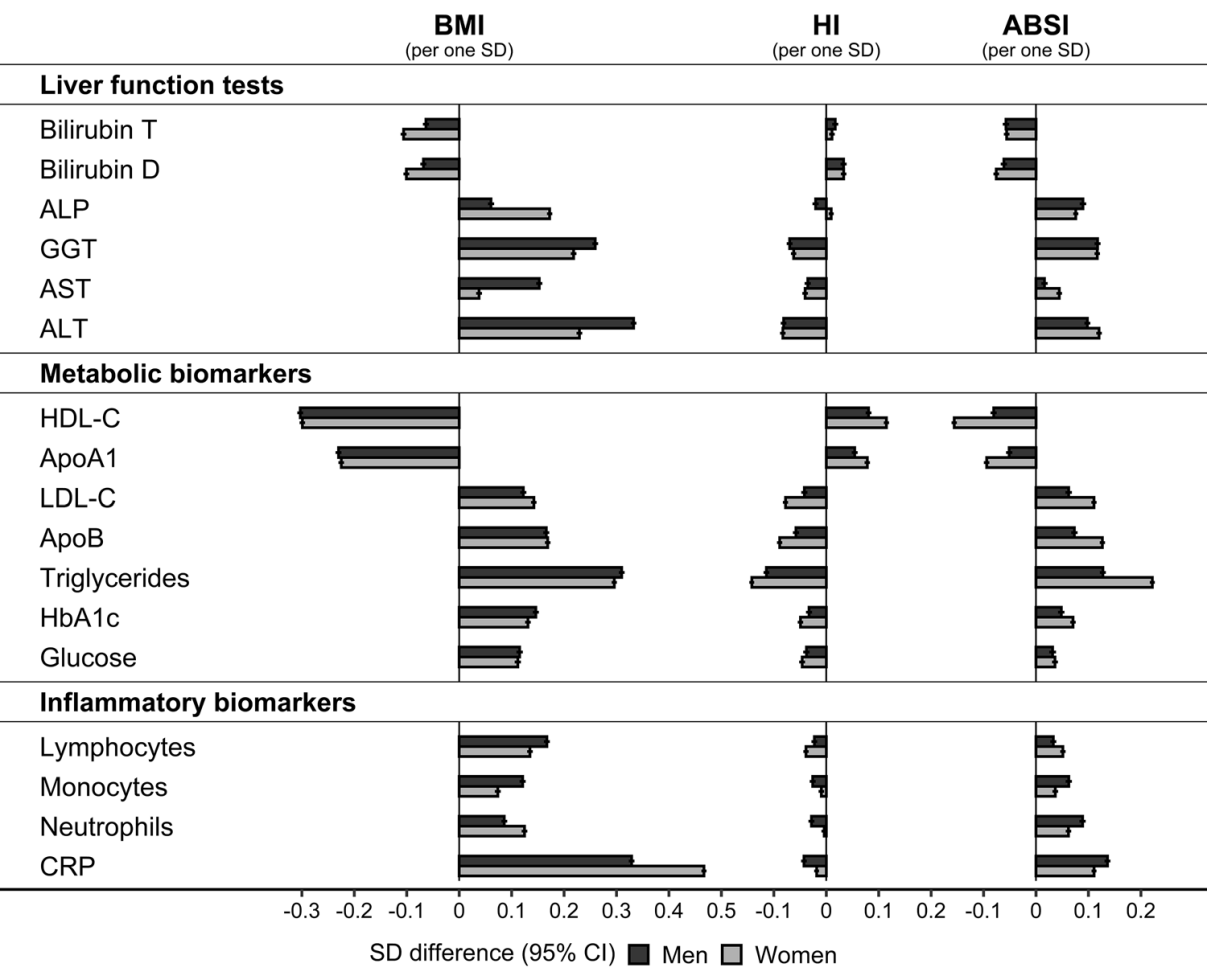
Associations of biomarkers with body size and body shape indices (continuous). ABSI – a body shape index; ALP – alkaline phosphatase; ALT – alanine aminotransferase; ApoA1 – apolipoprotein A1; ApoB – apolipoprotein B; AST – aspartate aminotransferase; Bilirubin D – direct bilirubin; Bilirubin T – total bilirubin; BMI – body mass index; CI – confidence interval; CRP – C-reactive protein; GGT – gamma-glutamyltransferase; HbA1c – haemoglobin A1c (glycated haemoglobin); HDL-C – high-density lipoprotein cholesterol; HI – hip index; LDL-C – low-density lipoprotein cholesterol; SD – standard deviation. SD difference (95% CI) – estimates obtained from multivariable linear regression models including each biomarker on a continuous standard deviation scale (sex-specific z-scores after log-transformation) as an outcome variable and BMI, ABSI, and HI on a continuous standard deviation scale (sex-specific z-scores), and covariates as independent variables. Covariates included height, age at enrolment, weight change within the last year preceding enrolment, smoking status, alcohol consumption, physical activity, Townsend deprivation index, region of the assessment centre, time of blood collection, fasting time, use of nonsteroidal anti-inflammatory drugs, paracetamol use, and in women, menopausal status, hormone replacement therapy use, oral contraceptives use, and age at the last live birth. Covariates are defined in Supplementary Methods. Numbers are shown in Supplementary Table S4.

BMI was associated inversely with bilirubin, HDL-C, and ApoA1 and positively with all other biomarkers. The associations with BMI were more prominent in women compared to men for bilirubin, ALP, neutrophils, and CRP but were more prominent in men compared to women for GGT, AST, ALT, lymphocytes, and monocytes (Fig. 1). ABSI was associated with all biomarkers in the same direction as BMI but more weakly, while HI was associated in the opposite direction and even more weakly than ABSI. Mainly ABSI, in addition to BMI, was associated with bilirubin, ALP, monocytes, neutrophils, and CRP. Both ABSI and HI were associated more strongly in women compared to men with metabolic biomarkers and lymphocytes but were associated more strongly in men compared to women with monocytes, neutrophils, and CRP. Associations with all three anthropometric indices were most prominent for GGT, ALT, HDL-C, ApoA1, triglycerides, and CRP (Fig. 1).

**Figure 2:**
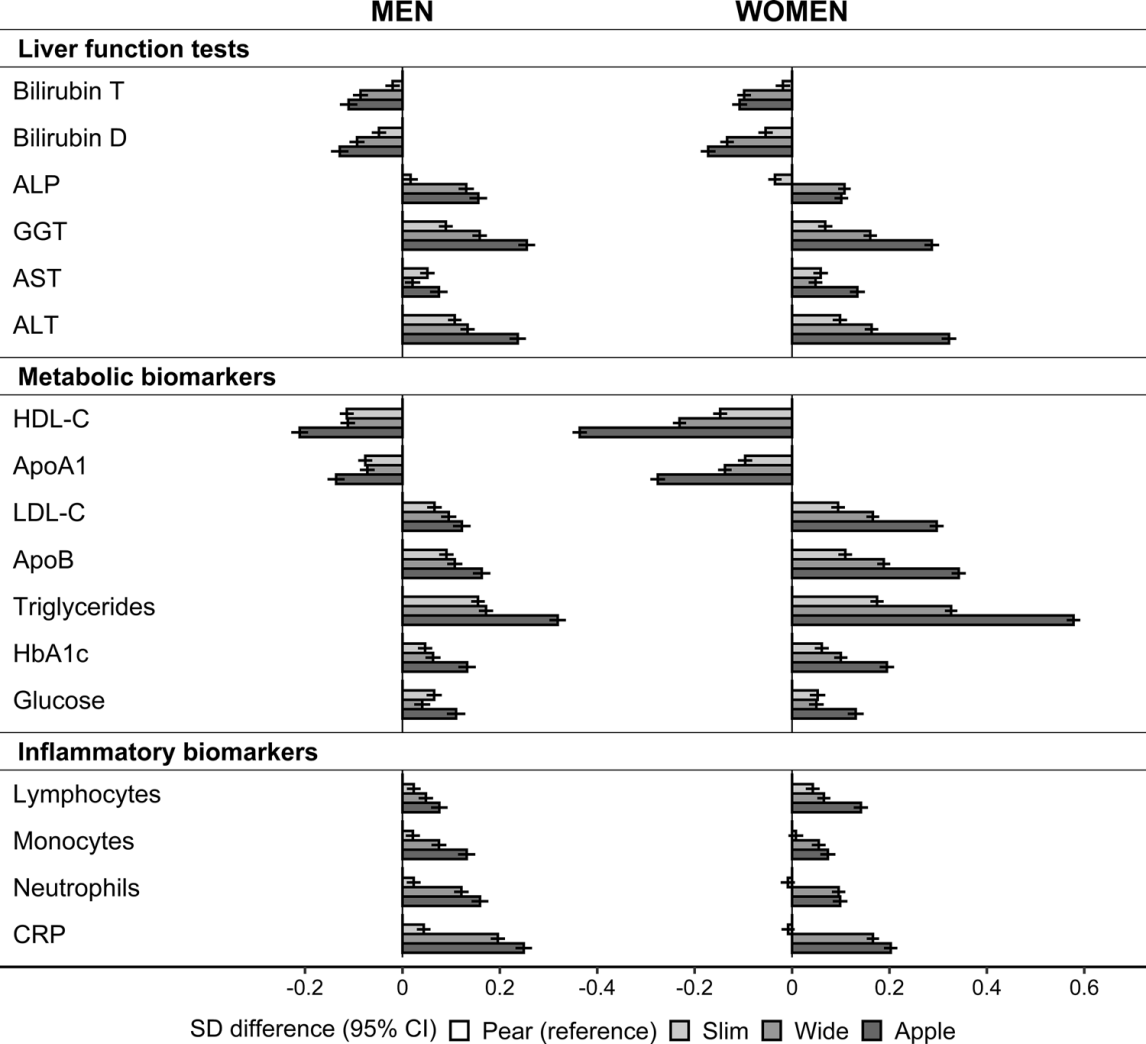
Associations of biomarkers with body shape phenotypes. ABSI – a body shape index (cut-offs ≥ 80 for men, ≥ 73 for women); ALP – alkaline phosphatase; ALT – alanine aminotransferase; ApoA1 – apolipoprotein A1; Apo B – apolipoprotein B; Apple – large-ABSI-small-HI; AST – aspartate aminotransferase; Bilirubin D – direct bilirubin; Bilirubin T – total bilirubin; BMI – body mass index; CI – confidence interval; CRP – C-reactive protein; GGT – gamma-glutamyltransferase; HbA1c – haemoglobin A1c (glycated haemoglobin); HDL-C – high-density lipoprotein cholesterol; HI – hip index (cut-offs ≥ 49 for men, ≥ 64 for women); LDL-C – low-density lipoprotein cholesterol; Ly – lymphocytes; Neu – neutrophils; NW – normal weight (BMI ≥ 18.5 to BMI < 25 kg/m^2^); OB – obese (BMI ≥ 30 to BMI < 45 kg/m^2^); OW – overweight (BMI ≥ 25 to BMI < 30 kg/m^2^); Pear – small-ABSI-large-HI; SD – standard deviation; Slim – small-ABSI-small-HI; Wide – large-ABSI-large-HI. SD difference (95% CI) – estimates obtained from multivariable linear regression models including each biomarker (sex-specific z-scores, following log-transformation) as an outcome variable and, as independent variables, an ABSI-by-HI cross-classification, BMI categories, and covariates. Covariates included height, age at enrolment, weight change within the last year preceding enrolment, smoking status, alcohol consumption, physical activity, Townsend deprivation index, region of the assessment centre, time of blood collection, fasting time, use of nonsteroidal anti-inflammatory drugs, paracetamol use, and in women, menopausal status, hormone replacement therapy use, oral contraceptives use, and age at the last live birth. Numbers are shown in Supplementary Table S5. A likelihood ratio test comparing a model including BMI categories and covariates with a model additionally including the ABSI-by-HI cross-classification (evaluates the overall significance of body shape phenotypes) showed *p* < 1*10^–6^ for all biomarkers (p-values are shown in Supplementary Table S5).

Combining dichotomised ABSI and HI in body shape phenotypes maximised the opposition of ABSI and HI, resulting in the largest difference between phenotypes with discordant waist and hip size (Fig. 2). The lowest levels of GGT, AST, ALT, and all metabolic biomarkers were in “pear” and the highest in “apple” phenotype, except for HDL-C and ApoA1, for which the lowest levels were in “apple” and the highest in “pear” phenotype. The predominant associations with ABSI resulted in the lowest bilirubin levels and the highest ALP and CRP levels for phenotypes with large waist size (“wide” and “apple”). Neutrophil and monocyte counts resembled the pattern of CRP, while lymphocyte counts resembled the pattern of metabolic biomarkers (Fig. 2).

### Heterogeneity of the associations of biomarkers with body shape according to body size

**Figure 3:**
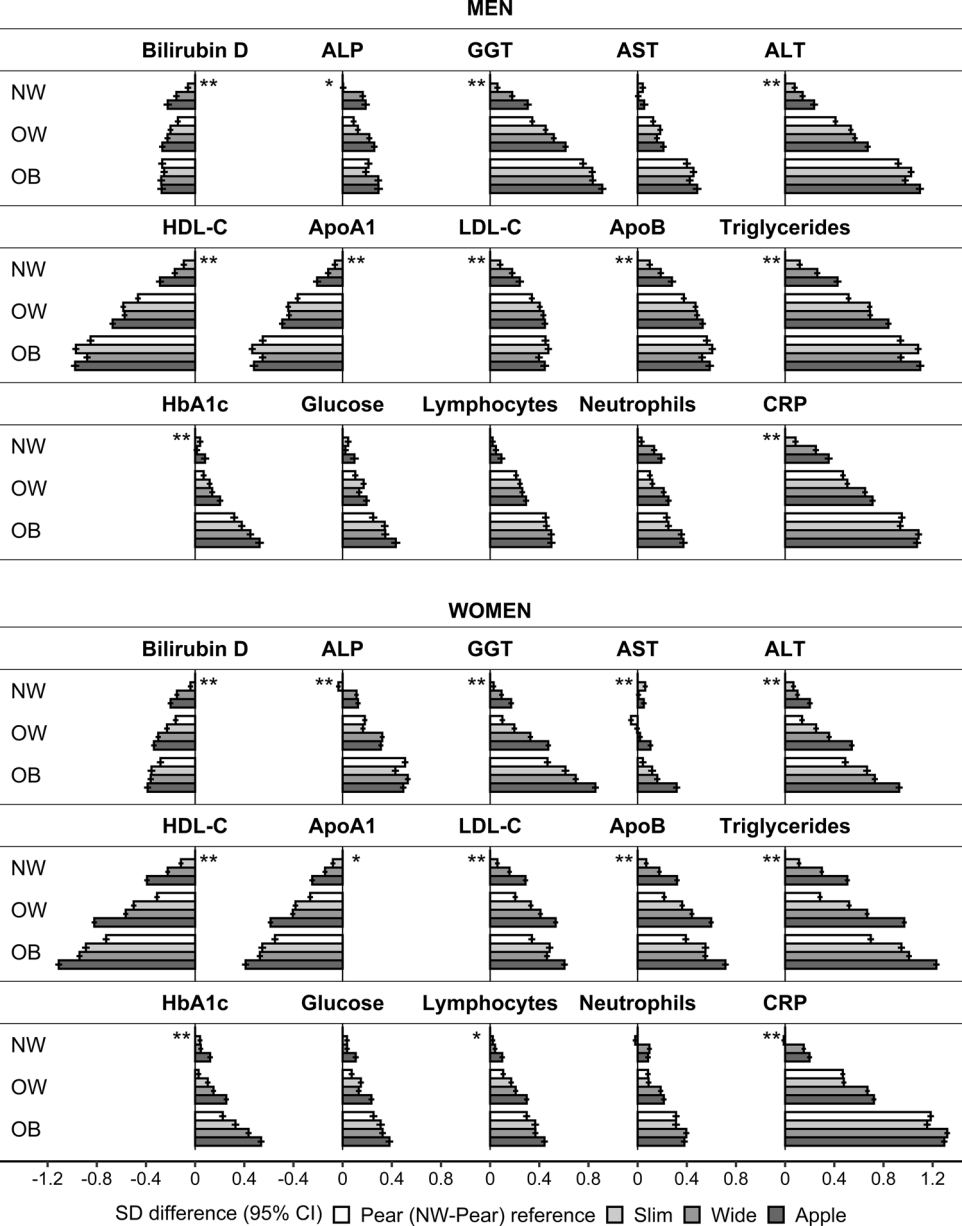
Heterogeneity of the associations of biomarkers with body shape phenotypes according to body size. ABSI – a body shape index (cut-offs ≥ 80 for men, ≥ 73 for women); ALP – alkaline phosphatase; ALT – alanine aminotransferase; ApoA1 – apolipoprotein A1; Apo B – apolipoprotein B; Apple – large-ABSI-small-HI; AST – aspartate aminotransferase; Bilirubin D – direct bilirubin; BMI – body mass index; CI – confidence interval; CRP – C-reactive protein; GGT – gamma-glutamyltransferase; HbA1c – haemoglobin A1c (glycated haemoglobin); HDL-C – high-density lipoprotein cholesterol; HI – hip index (cut-offs ≥ 49 for men, ≥ 64 for women); LDL-C – low-density lipoprotein cholesterol; Ly – lymphocytes; Neu – neutrophils; NW – normal weight (BMI ≥ 18.5 to BMI < 25 kg/m^2^); OB – obese (BMI ≥ 30 to BMI < 45 kg/m^2^); OW – overweight (BMI ≥ 25 to BMI < 30 kg/m^2^); Pear – small-ABSI-large-HI; SD – standard deviation; Slim – small-ABSI-small-HI; Wide – large-ABSI-large-HI. SD difference (95% CI) – estimates obtained from multivariable linear regression models including each biomarker (sex-specific z-scores, following log-transformation) as an outcome variable and, as independent variables, a BMI-by-ABSI-by-HI cross-classification and covariates (a single model for NW, OW, and OB). Covariates included height, age at enrolment, weight change within the last year preceding enrolment, smoking status, alcohol consumption, physical activity, Townsend deprivation index, region of the assessment centre, time of blood collection, fasting time, use of nonsteroidal anti-inflammatory drugs, paracetamol use, and in women, menopausal status, hormone replacement therapy use, oral contraceptives use, and age at the last live birth. Numbers are shown in Supplementary Table S5. p-values (* –* p* < 0.0001; ** – *p* < 1*10^–6^) – derived from a likelihood ratio test comparing an additive model including the ABSI-by-HI cross-classification, BMI categories, and covariates with the interaction model including the BMI-by-ABSI-by-HI cross-classification and covariates (evaluates heterogeneity by BMI) (p-values are shown in Supplementary Table S5).

Overall, the association patterns of biomarkers with body shape phenotypes were retained in all BMI categories (Fig. 3), but there were some exceptions. In obese men, AST, ALT, and lipid-related biomarkers showed almost exclusive associations with HI, such that aminotransferase and triglyceride levels were highest and HDL-C and ApoA1 levels were lowest for phenotypes with small hip size (“slim” and “apple”). Also in obese men, there was little evidence for association of bilirubin, LDL-C, or lymphocytes with body shape. In obese women, there was a preferential positive association of ALP with HI, with the highest levels for phenotypes with large hip size (“pear” and “wide”). All women with “apple” phenotype in a lower BMI category had a worse lipid profile (i.e. higher triglycerides, LDL-C, and ApoB, and lower HDL-C) compared to women with “pear” phenotype in a higher neighbouring BMI category. In both men and women, associations with AST were similar to ALT but weaker. Aminotransferases resembled the pattern of triglycerides, while GGT resembled more closely the pattern of HbA1c (Fig. 3).

### Consistency of the associations of biomarkers with body size and body shape across biomarker levels

**Figure 4:**
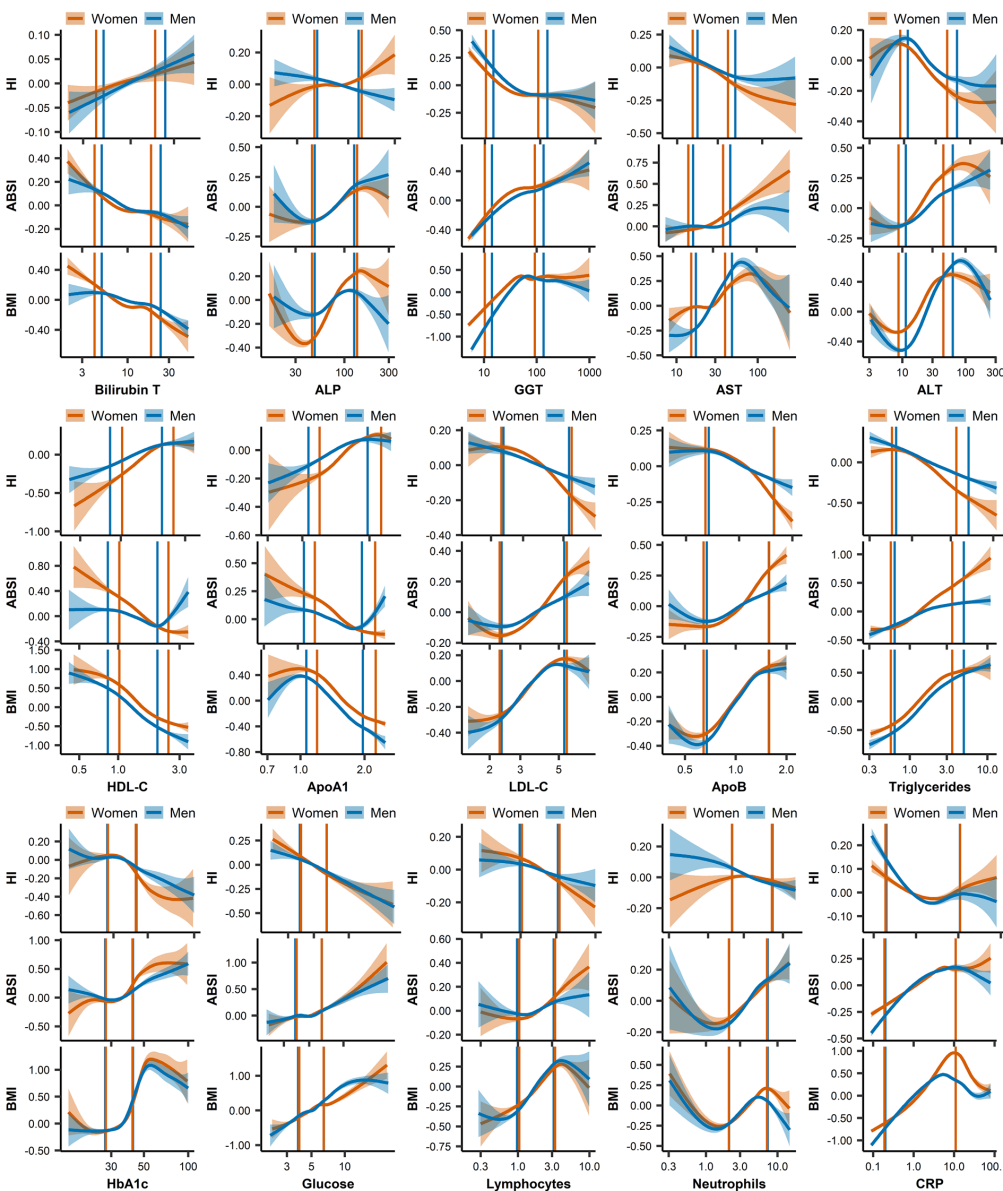
Associations of biomarkers with body size and body shape indices across biomarker levels. ABSI – a body shape index; ALP – alkaline phosphatase (U/L); ALT – alanine aminotransferase (U/L); ApoA1 – apolipoprotein A1 (g/L); ApoB – apolipoprotein B (g/L); AST – aspartate aminotransferase (U/L); Bilirubin T – total bilirubin ($\upmu$mol/L); BMI – body mass index; CRP – C-reactive protein (mg/L); GGT – gamma-glutamyltransferase (U/L); Glucose (mmol/L); HbA1c – haemoglobin A1c (glycated haemoglobin) (mmol/mol); HDL-C – high-density lipoprotein cholesterol (mmol/L); HI – hip index (cut-offs ≥ 49 for men, ≥ 64 for women); LDL-C – low-density lipoprotein cholesterol (mmol/L); Lymphocytes (*10^9^/L); Neutrophils (*10^9^/L); Triglycerides (mmol/L). Plots represent mean of biomarker levels (95% confidence interval), determined with the generalised additive models smoothing function from package mgcv v1.8–39, applied in ggplot2 v.3.3.5 in R v4.1.3., which uses restricted maximum likelihood (REML) estimation. Vertical lines represent 2.5th and 97.5^th^ sex-specific centiles of the dataset. Extreme values were removed, to avoid leverage on the estimates (see details in Supplementary Table S6). For each of the anthropometric indices, we used residuals derived from a multivariable linear regression model including the other two anthropometric indices and covariates. Covariates comprised height, age at enrolment, weight change within the last year preceding enrolment, smoking status, alcohol consumption, physical activity, Townsend deprivation index, region of the assessment centre, time of blood collection, fasting time, use of nonsteroidal anti-inflammatory drugs, paracetamol use, and in women, menopausal status, hormone replacement therapy use, oral contraceptives use, and age at the last live birth.

Across biomarker levels, associations with anthropometric indices were largely consistent with the observations reported for the total dataset within the central part of the biomarker distributions but were weaker or absent towards the tails (Fig. 4). Models with restricted cubic splines provided strong evidence for a plateau in the associations with BMI for high levels of lipid-related biomarkers and CRP, and in men also of GGT and ALT (Supplementary Table S4, Fig. 4). There was also a weaker evidence for a similar pattern of associations with ABSI, while the associations of BMI with HbA1c were J-shaped (Supplementary Table S4, Fig. 4). Subgroup analysis with fully adjusted linear models supported a change of direction for very low or very high biomarker levels (*p* < 0.0001 for the small tail-end group) for the following associations. In men, ALT above 70 U/L and CRP above 8 mg/L were associated inversely with BMI. Also in men, HDL-C above 1.8 mmol/L and ApoA1 above 1.8 g/L were associated positively with ABSI. In women, neutrophils below 2*10^9^/L and CRP above 8 mg/L were associated inversely with BMI (Fig. 4, Supplementary Table S7).

### Associations between biomarkers, independent of body size and body shape

**Figure 5:**
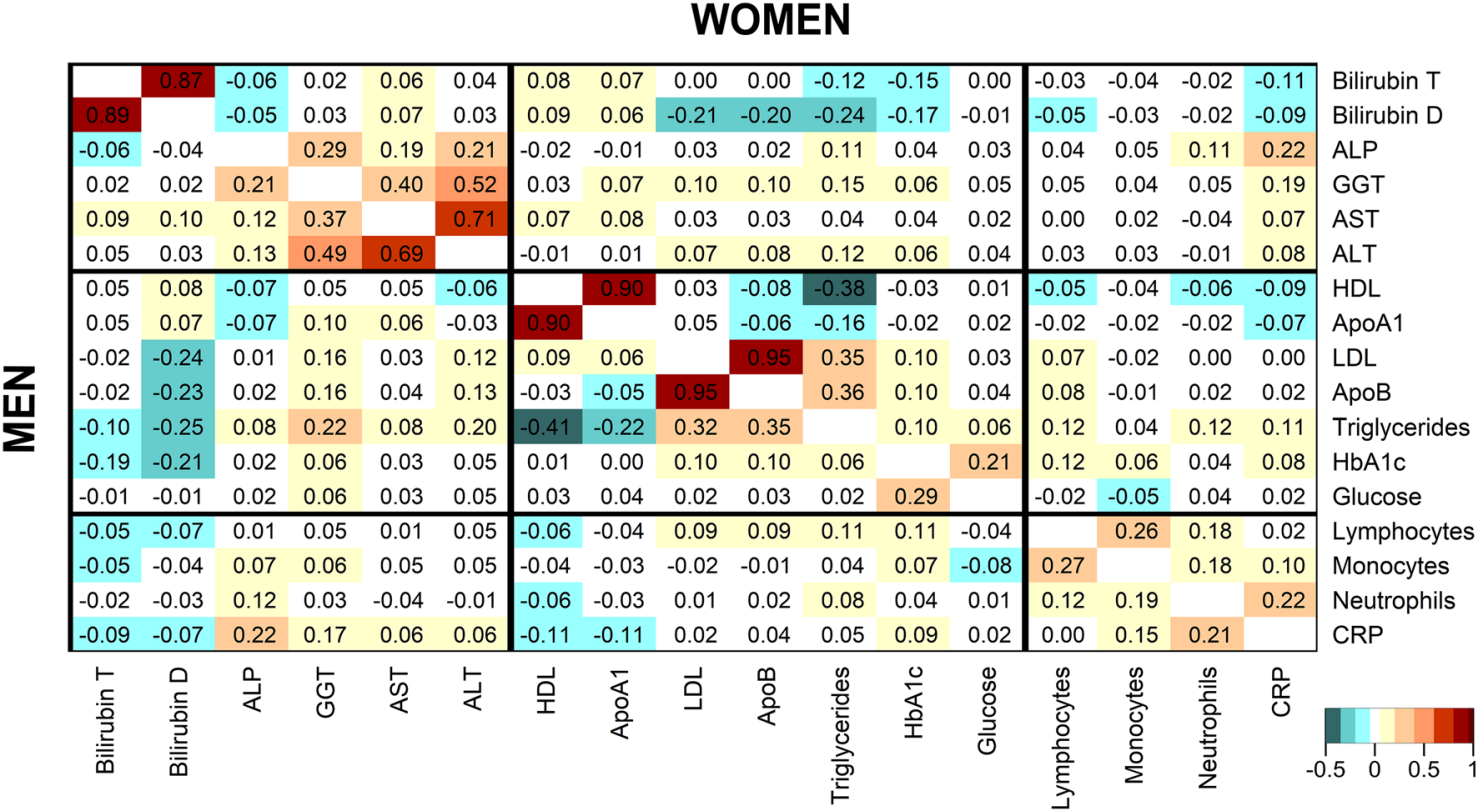
Correlations between biomarkers, independent of body size and body shape. ABSI – a body shape index; ALP – alkaline phosphatase; ALT – alanine aminotransferase; ApoA1 – apolipoprotein A1; ApoB – apolipoprotein B; AST – aspartate aminotransferase; Bilirubin D – direct bilirubin; Bilirubin T – total bilirubin; BMI – body mass index; CRP – C-reactive protein; GGT – gamma-glutamyltransferase; HbA1c – haemoglobin A1c (glycated haemoglobin); HDL-C – high-density lipoprotein cholesterol; HI – hip index; LDL-C – low-density lipoprotein cholesterol; Ly – lymphocytes; Mo – monocytes; Neu – neutrophils. Values represent partial Pearson correlation coefficients (men – bottom-left; women – top-right) with adjustment for BMI, ABSI, HI, height, age at enrolment, Townsend deprivation index, time of blood collection, fasting time (continuous), weight change within the last year preceding enrolment, smoking status, alcohol consumption, physical activity, use of nonsteroidal anti-inflammatory drugs, paracetamol use, and in women, menopausal status, hormone replacement therapy use, and oral contraceptives use.

Independent of anthropometric indices and other covariates, total and direct bilirubin were correlated inversely with triglycerides and HbA1c and direct bilirubin was additionally correlated inversely with LDL-C and ApoB (Fig. 5). ALP and GGT were correlated positively with each other and with CRP. GGT was also correlated positively with ALT and both were correlated positively with LDL-C, ApoB, and triglycerides, more strongly in men than in women. Triglycerides were correlated substantially inversely with HDL-C and more weakly with ApoA1, but were similarly positively correlated with LDL-C and ApoB. HbA1c and glucose were mainly correlated positively with each other but not with lipid-related biomarkers. CRP was correlated positively with neutrophils but not with lymphocytes, while lymphocytes were correlated most strongly positively with monocytes (Fig. 5).

### Sensitivity analyses

Unadjusted models showed that adjustment for covariates had attenuated to some extent the associations with BMI and ABSI for most biomarkers, except for the associations of ABSI with HDL-C and ApoA1, which were stronger after adjustment, especially in women (Supplementary Figure S2). The association patterns with body shape phenotypes combining ABSI and HI were overall retained in unadjusted models, except for larger differences between “pear” and “apple” phenotypes compared to the fully adjusted models for HbA1c (Supplementary Figure S3).

Subgroups with stable weight or without medication use represented the larger part of the dataset (54.4% to 71.7%, Table 1). The fully adjusted subgroup models showed no material difference from the patterns described for the complete dataset, except from marginally stronger associations of BMI with lipid-related biomarkers and CRP, and in men also with GGT and ALT, in the subgroup with stable weight (Supplementary Figure S4). In subgroups with NSAID or paracetamol use, only the associations of BMI with HDL-C, ApoA1, and in men also with ALT, triglycerides, and CRP were marginally weaker (Supplementary Figure S4). The association patterns with body shape phenotypes described for the complete dataset were largely retained, including in the subgroups with NSAID or paracetamol use (Supplementary Figure S5).

In subgroups with higher alcohol consumption, associations of BMI with liver function tests, lipid-related biomarkers, and CRP were stronger than in the subgroup with low alcohol consumption, as were associations of ABSI with GGT, AST, and ALT, while associations of ABSI and HI with HDL-C and ApoA1, and in women with HbA1c, were weaker (Supplementary Figure S6). There was no material difference, however, in the association patterns with body shape phenotypes, except that in obese men with higher alcohol consumption was retained a positive association of ALT with ABSI, in addition to the inverse association with HI (Supplementary Figure S7).

## Discussion

Using the allometric body shape indices ABSI and HI, we have shown positive associations with waist size and inverse associations with hip size, independent of positive associations with BMI, for GGT, ALT, LDL-C, ApoB, triglycerides, HbA1c, glucose, and lymphocytes, with the lowest levels for “pear” and highest for “apple” phenotype, while associations with HDL-C and ApoA1 were inverse with BMI and ABSI but positive with HI, with the lowest levels for “apple” and highest for “pear” phenotype. Inflammatory biomarkers, represented by CRP, neutrophils, and monocytes, as well as ALP, were only associated positively with waist and not with hip size, independent of positive associations with BMI, while bilirubin was associated inversely with BMI and waist size but not with hip size. The patterns were similar for all BMI categories, except that ALT and lipid-related biomarkers were associated only with hip size in obese men and ALP was associated positively with hip size in obese women.

Studies examining associations of allometric body shape indices with metabolic and inflammatory biomarkers remain scarce and focused exclusively on ABSI, without considering HI. Positive associations with ABSI, as well as with BMI, have been reported for triglycerides and fasting glucose, together with inverse associations for HDL-C^20^. Men with large ABSI have additionally shown lower insulin sensitivity^21^, while women with large ABSI have shown a larger proportion of the pro-atherogenic small dense LDL particles^22^. Large ABSI has also been associated with higher CRP^21,23^, and in agreement with our findings, CRP has previously shown stronger positive associations with BMI in women compared to men^23^. Notably, however, several studies have reported an inferior discrimination of ABSI compared to BMI or traditional waist-circumference-based indices for components of the metabolic syndrome^24^, type 2 diabetes^16,25^, or high ALT levels^26^. This should not be surprising, given that BMI is associated with metabolic factors and conditions and any measure of waist size strongly correlated with BMI, such as waist circumference, would carry the same information as BMI, while ABSI is independent of BMI by design and should be interpreted not as an alternative but as a complement to BMI.

Previous studies have also extensively noted positive associations of ALT and GGT but not AST with BMI and waist circumference, unexplained by hepatitis or alcohol consumption, along with positive associations of ALT and GGT with triglycerides, LDL particles, fasting insulin, and insulin resistance, as well as inverse associations with HDL-C^26–32^, but no attention has been paid to hip size. The similarities in the association patterns of ALT and GGT noted in our and previous studies would likely be related to their shared genetic background and the differences between obese men and women reported in our study would likely be related to the sexually dimorphic relative contribution of individual genes^33^. Physiologically, ALT participates in the glucose-alanine cycle, transferring ammonium groups from amino acids to pyruvate released from glycolysis in the muscle, thus producing alanine, which in the liver is converted back to pyruvate and is used to generate glucose in gluconeogenesis^34^. Correspondingly, intervention studies in humans support a more important role of insulin than lipids for ALT regulation, as carbohydrate restriction contributes to a greater reduction in ALT compared to fat restriction, despite similar weight reductions with alternative low-energy diets^35^. Nevertheless, statin administration in humans reduces ALT and GGT levels^36^, and in animal models, cholesterol-depleted but not cholesterol-loaded HDL particles induce ALT release from the liver to the circulation^37^, which is in agreement with the association patterns of ALT matching more closely lipid-related than glucose-related biomarkers in our study. GGT, on the other hand, is a membrane-bound ectoenzyme, hydrolysing gamma-glutamyl bonds of glutathione and its S-conjugates with xenobiotics, and as such represents part of the cellular antioxidant system but can also have a pro-oxidative action and has been implicated in the pathogenesis of atherosclerosis (via oxidation of LDL-C), inflammatory conditions, and cancer^38^.

Notably, the association pattern with body shape phenotypes characteristic of metabolic biomarkers, ALT, and GGT, with lowest levels for “pear” and highest levels for “apple” phenotype, corresponds to the association pattern of visceral adipose tissue (VAT) that we have previously described, while the exclusive association of lipid-related biomarkers and ALT with hip size observed in obese men corresponds to the pattern of gluteofemoral fat mass^14^. This suggests that in addition to factors related to or originating from VAT, there is also an involvement, at least in men, of a factor that either originates from gluteofemoral fat or determines its accumulation. One such factor could be oestradiol originating from peripheral aromatisation in adipose tissue. This would be compatible with the sexual dimorphism of lipids described in our and other studies, with lower levels of triglycerides and ALT and higher HDL-C and ApoA1 in women compared to men^39^.

Chronic low-grade inflammation is characteristic of obesity and can contribute to the generation for dysfunctional HDL particles^40,41^. In our study, however, hip size was associated only with glucose-related and lipid-related factors but not with CRP or neutrophils, highlighting differences in the underlying mechanisms. Low-grade inflammation is mediated by macrophage infiltration of the adipose tissue^42^. Nevertheless, macrophages play a complex role, as their classical activation contributes to a pro-inflammatory phenotype, promoting the development of insulin resistance and type 2 diabetes, while their alternative activation improves insulin sensitivity^43^. The classical activation of macrophages can also be triggered by tissue infiltration with neutrophils, which thus contribute to the maintenance of chronic low-grade inflammation, in addition to their key role in the acute inflammatory response^44^. Correspondingly, mice with neutropenia have reduced liver lipogenesis and steatosis^45^. In humans, neutrophil counts are higher in hyperlipidaemia, hyperglycaemia, and insulin resistance even in healthy individuals^28,44^, and are accompanied with higher lymphocyte and monocyte counts and higher CRP levels in obesity and the metabolic syndrome^46,47^. An upregulation of genes related to neutrophil degranulation has also been reported in patients with cardiovascular diseases^48^. Neutrophil counts, however, are reduced after bariatric surgery proportional to the changes in BMI and insulin resistance, while lymphocyte counts are not affected materially and the response of monocytes varies according to the surgical technique^49^. The latter is in agreement with the similar associations with body shape for CRP, neutrophils, and partially for monocytes but not for lymphocytes observed in our study.

A likely mechanistic factor explaining the positive association of waist size with metabolic and inflammatory biomarkers would be cortisol, as glucocorticoids play a key role in the regulation of the anti-inflammatory response and the hypothalamus–pituitary–adrenal axis is dysfunctional in obesity, favouring VAT accumulation, metabolic alterations, and abdominal obesity^50^. In animal models, neutrophil infiltration of mouse liver and secretion of neutrophil elastase is accompanied with activation of clock genes and follows a circadian rhythm, with the lowest neutrophil counts in liver at lights-off time and the highest at lights-on time, corresponding to the circulating corticosterone levels^45,51^. In humans, glucocorticoids contribute to higher circulating neutrophil counts via increased release of polymorphonuclear cells from the bone marrow and from the marginalised pool (cells attached to the endothelial surface), as well as by delayed apoptosis^52^. It remains unclear, however, what is the contribution of glucocorticoids to associations with hip size and how they interact with other factors related to body shape.

Bilirubin and ALP, similarly to inflammatory biomarkers, were associated only with BMI and waist and not with hip size, but unlike their concomitant increase in obstructions of the biliary tract, their associations with anthropometric indices were discordant. In agreement with our findings, higher levels of the total and liver fraction of ALP in serum have been reported in obesity and it has been shown that tissue ALP is involved in lipid metabolism and adipokine synthesis^53,54^. Obesity is also associated with higher expression of leucocyte ALP (*ALPL*) gene in neutrophils, a marker of neutrophil activation^55^, which is compatible with an involvement of ALP in the inflammatory response, in agreement with the positive correlation of ALP with CRP and neutrophil counts observed in our study. Further in agreement with our findings, lower bilirubin levels have been reported in obesity without metabolic complications^56^, as well as in type 2 diabetes and the metabolic syndrome^57^. Bilirubin also plays a protective role against liver lipid infiltration and the development of NAFLD^58^, and animal models have shown that biliverdin, a bilirubin precursor in the haem catabolic pathway, contributes to smaller adipocyte size and suppresses inflammatory factors, thus reducing insulin resistance^59^.

Our study has shown that associations with body size and body shape related to obesity hold within the clinical reference ranges of biomarker levels, while for lower or higher levels, pathological conditions other than obesity would likely gain leverage. Thus, severe chronic inflammatory conditions and liver damage can contribute to skeletal muscle wasting and cachexia^60^, potentially explaining the inverse associations of BMI with high ALT and CRP observed in our study in men and for high CRP, also in women. Further, the inverse associations of BMI with low neutrophil counts, most prominent in our study for women, could be related to a secondary autoimmune type neutropenia accompanying chronic inflammatory or autoimmune conditions^61^. Although high HDL-C is generally considered beneficial, U-shaped associations have been reported for HDL-C, with a positive association with ALT and AST at high HDL-C^62^. This, together with the positive association of high HDL-C and ApoA1 with ABSI observed in our study in men, suggests un underlying pathological condition for very high HDL-C levels, which merits further investigation.

Our study benefited from a very large sample size, which enabled us to examine in more detail some relatively small subgroups. There was also a detailed information for covariates, which permitted adjustment for major lifestyle and reproductive factors and minimised confounding. The standardised anthropometric measurements, obtained by trained personnel, avoided bias from self-reported values. The standardised approach to biomarker measurements, with a unified and systematic quality control for all samples, minimised measurement errors. Due to limited numbers, however, we could not examine underweight or severe obesity, or ethnic variations, or pre-menopausal women, or younger men, or longitudinal associations, or association of biomarkers with imaging measurement of body composition, which were obtained a few years later for a small part of the UK Biobank cohort. A misclassification of medication use is also possible, as the information was self-reported and was assembled from several questions. Importantly, our study was cross-sectional, and as such could not assess temporality or provide strong insights for potential causality. Although we have removed participants with known underlying conditions potentially influencing body composition or contributing to weight change (prevalent cancer and non-cancer illness or medication use at enrolment, or incident cancer and death within the first two years after enrolment), thus retaining only half of the original UK Biobank dataset, some possibility for reverse causality from subclinical or unreported conditions remains. Nevertheless, body size and body shape and the underlying body composition, as well as biomarker levels, are endogenous factors. As such, they are likely interrelated in complex causal networks, rather than in linear causal pathways, with each other and with other endogenous factors such as sex steroids and glucocorticoids, as well as with exogenous and genetic factors. In this context, our findings suggest that body size and body shape and their determining factors are more likely to be leading a direct association within the central part of the biomarker distributions. Towards the tails of the biomarker distributions, however, biomarkers and their determining factors and associated diseases are more likely to be contributing to reverse causality. Finally, UK Biobank participants are not only relatively older, but have a healthier lifestyle and are not representative of the overall UK population^63^. This discrepancy would be aggravated further by the removal of participants with prevalent illnesses at enrolment or using medications.

In conclusion, glucose-related and lipid-related biomarkers are associated in opposite directions with waist and hip size, independent of overall body size, while inflammatory biomarkers are associated only with waist size, suggesting differences in the underlying mechanisms. Associations with body size and body shape related to obesity remain consistent within the clinical reference ranges of biomarker levels, but are lost or change direction for low or high levels, potentially reflecting the influence of chronic inflammatory or autoimmune conditions.

## Supplementary Information

Supplementary Information.

## Data Availability

The data supporting the findings of the study are available *to bona fide* researchers upon approval of an application to the UK Biobank (https://www.ukbiobank.ac.uk/researchers/) and a material transfer agreement.

## Abbreviations

**ABSI**: A body shape index
**BMI**: Body mass index
**CI**: Confidence interval
**HC**: Hip circumference
**HI**: Hip index
**HR**: Hazard ratio
**NHANES**: National Health and Nutrition Examination Survey
**SD**: Standard deviation
**WC**: Waist circumference
