# Supplementary material for: Associations of body shape index (ABSI) and hip index with liver, metabolic, and inflammatory biomarkers in the UK Biobank cohort
Source: Sci Rep. 2022 May 25;12:8812. doi: 10.1038/s41598-022-12284-4 (PMC9133113; doi:10.1038/s41598-022-12284-4)
Supplement: Supplementary file 1 — Supplementary Information. [file 41598_2022_12284_MOESM1_ESM.pdf]

# Associations of body shape index (ABSI) and hip index with liver, metabolic, and inflammatory biomarkers in the UK Biobank cohort

Sofia Christakoudi, Elio Riboli, Evangelos Evangelou, Konstantinos K. Tsilidis

**Supplementary Methods**

Definition of covariates ……………………………………………………………………………..3

**Supplementary Tables**

Supplementary Table S1 Categories of self-reported medications ............................................ 6

Supplementary Table S2 Biomarker availability........................................................................ 8

Supplementary Table S3 Characteristics of study participants by sex and body shape phenotypes ................................................................ 10

Supplementary Table S4 Linear and non-linear associations of biomarkers with body size and body shape indices (continuous).............................................. 13

Supplementary Table S5 Associations of biomarkers with body shape phenotypes (categorical)........................................................................... 16

Supplementary Table S6 Biomarker boundaries..................................................................... 20

Supplementary Table S7 Associations of biomarkers with body size and body shape indices: subgroups according to biomarker levels........................................ 21

**Supplementary Figures**

Supplementary Figure S1 Flow diagram of UK Biobank participants in the study ..................... 23

Supplementary Figure S2 Associations of biomarkers with body size and body shape indices (continuous, unadjusted) ................................................................ 26

Supplementary Figure S3 Associations of biomarkers with body shape phenotypes (categorical, unadjusted) ................................................................ 28

Supplementary Figure S4A Associations of biomarkers with body size and shape indices: excluding weight loss or medication use....................................... 30

Supplementary Figure S4B Associations of biomarkers with body size and body shape indices: subgroups with medication use .................................................... 31

Supplementary Figure S5A Associations of biomarkers with body shape phenotypes: subgroup without recent weight change ........................................ 33

Supplementary Figure S5B Associations of biomarkers with body shape phenotypes: subgroup without NSAID or paracetamol use ................................ 34

Supplementary Figure S5C Associations of biomarkers with body shape phenotypes: subgroup with NSAID use ............................................................ 35

Supplementary Figure S5D Associations of biomarkers with body shape phenotypes: subgroup with paracetamol use ................................................... 36

Supplementary Figure S6 Associations of biomarkers with body size and body shape indices: subgroups according to alcohol consumption ................................ 38

Supplementary Figure S7A Associations of biomarkers with body shape phenotypes: up to 3 times a month alcohol consumption .................................. 40

Supplementary Figure S7B Associations of biomarkers with body shape phenotypes: up to four times a week alcohol consumption ................................ 41

Supplementary Figure S7C Associations of biomarkers with body shape phenotypes: daily alcohol consumption ........................................................... 42

**References** ............................................................................................ 44

# Supplementary Methods

## Definition of covariates

The following variables were defined as previously described [ref. 10]: age at enrolment (continuous scale, 5 years increment), region of the assessment centre (London, North-West, North-East, Yorkshire and Humber, West Midlands, East Midlands, South-East, South-West, Wales, Scotland), weight change during the last year preceding enrolment (weight loss, stable weight, weight gain), smoking status (never smoked, former occasional smoker, former regular smoker, current smoker), alcohol consumption (≤3 times/month, ≤4 times/week, daily), physical activity (less active, moderately active, very active), prevalent and incident cancers, and deaths (used for exclusions), and in women, hormone replacement therapy (HRT) use (never, past, current), and age at the last live birth (no live births, <30 years, ≥30 years). Missing values were assigned the sex-specific median category for the study dataset as follows: weight change (stable weight), smoking status (former occasional smoker), alcohol consumption (≤4 times/week), physical activity (moderately active), HRT use (never use), age at the last live birth (<30 years).

Fasting time was based on Field [74-0.0] "*Fasting time*" and was used on a continuous scale for the adjustment of partial correlations and categorised for the adjustment of multivariable linear models, with three categories defined as follows: 0-2 hours, 3-4 hours, 5 or more hours. Samples with unknown fasting time were assigned the sex-specific median value (3 hours for both sexes), or category (3-4 hours).

Time of blood collection was extracted from Field [3166-0.0] "*Time blood sample collected*", calculating time in decimal hours as: (Hours * 3600 + Minutes * 60 + Seconds) / 3600. This variable was used on a continuous scale for the adjustment of partial correlations and was categorised for the adjustment of multivariable linear models, with three categories defined as follows: Morning (≥8 to <12 o'clock, first at 8:25), Afternoon (≥12 to <16 o'clock), Evening (≥16 o'clock, last at 21:05). Unknown times of blood collection were assigned the sex-specific median value (14.371 for women, 14.533 for men) or category (Afternoon).

Townsend deprivation index was based on Field [189-0.0] "*Townsend deprivation index at recruitment*" and was used as an indicator of socioeconomic status on a continuous scale for the adjustment of partial correlations and as terciles for the adjustment of multivariable linear models. Missing values were replaced with the sex-specific median value (-2.374 for women, -2.344 for men) and then terciles were defined with the following study-specific boundaries: -3.291 and -1.046 for women; -3.285 and -0.955 for men.

Diabetes status at enrolment was based on Field [2443-0.0] "*Diabetes diagnosed by doctor*", Question: "*Has a doctor ever told you that you have diabetes?*", Answer: 1 "*Yes*" (for category Yes), or answer 0 "No" (for category No), considering Answer -1 "*Do not know*" and -3 "*Prefer not to answer*" as missing information. We further re-defined women with gestational diabetes only, answering 1 "Yes" to Field [4041-0.0] "*Gestational diabetes only*", Question: "*Did you only have diabetes during pregnancy?"* into category No with respect to diabetes status at enrolment. We also re-classified to category Yes participants with self-reported diabetes: codes: 1220 "diabetes", 1222 "type 1 diabetes", or 1223 "type 2 diabetes" in Fields [20002-0.0/33] "*Non-cancer illness code, self-reported*". We additionally re-classified as category Yes participants with self-reported insulin use, in men from Fields [6177-0.0/2] "*Medication for cholesterol, blood pressure or diabetes*", Question "*Do you regularly take any of the following medications? (you can select more than one answer)*", Answer 3 "*Insulin*", and in women from Field [6153-0/3] "*Medication for cholesterol, blood pressure, diabetes, or take exogenous hormones",* Answer 3 *"Insulin".* Last, we re-classified as category Yes participants with self-reported antidiabetic drugs from Fields [20003-0/47] "Treatment/medication code2" (see list of medication codes in Supplementary Table S1). It was not possible to discriminate between type 1 and type 2 diabetes based on the available information. Participants with missing values were assigned the median sex-specific category (No).

Lipid lowering drugs use was based on Fields [6153-0.0/3] in women and Fields [6177-0.0/2]. Participants providing Answer 1 "*Cholesterol lowering medication*" were assigned to category Yes and those providing answer -7 "None of the above", or only answers 2 "Blood pressure medication", or 3 "Insulin", or in women 4 "Hormone replacement therapy", or 5 "Oral contraceptive pill or minipill" were assigned to category No. We then re-classified to category Yes participants with self-reported lipid-lowering drugs from Fields [20003-0/47] "Treatment/medication code2" (see list of medication codes in Supplementary Table S1, including fibrates as well as statins). Participants with missing values were assigned the median sex-specific category (No).

Anti-hypertensive drugs use was based on Fields [6153-0.0/3] in women and Fields [6177-0.0/2]. Participants providing Answer 2 "*Blood pressure medication*" were assigned to category Yes and those providing answer -7 "*None of the above*", or only answers 1 "*Cholesterol lowering medication*", or 3 "*Insulin*", or in women 4 "*Hormone replacement therapy*", or 5 "*Oral contraceptive pill or minipill*" were assigned to category No. For the remaining participants, this information was considered missing and for them we used the sex-specific median category (No).

Nonsteroidal anti-inflammatory drugs (NSAID) use (Aspirin & Ibuprofen) was based, as in [ref. 14], on Fields [6154-0.1/5] "*Medication for pain relief, constipation, heartburn*"; Question: "*Do you regularly take any of the following? (You can select more than one answer)*"; Answer 1: "*Aspirin*" OR Answer 2: "*Ibuprofen*" for category Yes (n=51,941 in the final study dataset). Category No was defined as any of the following Answers: 3 "*Paracetamol*", 4 "*Ranitidine*", 5 "*Omeprazole*", 6 "*Laxatives*" OR -7 "*None of the above*". We then re-classified to category Yes participants with

self-reported NSAID use from Fields [20003-0/47] “Treatment/ medication code2” (see list of medication codes in Supplementary Table S1) (further n=1689). Participants with missing values were assigned the median sex-specific category (No).

Paracetamol use was based on Fields [6154-0.1/5] “*Medication for pain relief, constipation, heartburn*”, Answer 3 “*Paracetamol*” for category Yes (n=53,652 in the final study dataset). Category No was defined as any of the following Answers: 1 “*Aspirin*”, 2 “*Ibuprofen*”, 4 “*Ranitidine*”, 5 “*Omeprazole*”, 6 “*Laxatives*” OR -7 “*None of the above*”. We then re-classified to category Yes participants with self-reported paracetamol use from Fields [20003-0/47] “Treatment/ medication code2” (see list of medication codes in Supplementary Table S1) (further n=767). Participants with missing values were assigned the median sex-specific category (No).

Oophorectomy (bilateral) was used to define menopausal status and was based on Field: [2834-0.0] “*Bilateral oophorectomy (both ovaries removed)*”; Question: "*Have you had BOTH ovaries removed?*"; Answer 1: “*Yes*” OR an answer to Fields [20004-0.0/31] “*Operation code (self-reported operation)*” including code: 1355 “*bilateral oophorectomy*”.

Hysterectomy was used to define menopausal status and was based on Field: [3591-0.0] “*Ever had hysterectomy (womb removed)*”; Question: "*Have you had a hysterectomy (womb removed)?";* Answer 1: “*Yes*” OR an answer to Fields [20004-0.0/31] “*Operation code (self-reported operation)*” including codes: 1357 “*hysterectomy*”, 1358 “*hysterectomy with oophorectomy*”, or 1359 “*hysterectomy with cervical sparing*”.

Menopausal status (MP) was defined in three categories: Post-menopausal were classified women with bilateral oophorectomy OR with self-reported post-menopausal status from Field [2724-0.0] “*Had menopause*”; Question: "*Have you had your menopause (periods stopped)?*"; Answer 1: “Yes”; Pre-menopausal were classified women who had not been defined as post-menopausal above AND had reported pre-menopausal status with Answer 0: “No” to Field [2724-0.0] AND had not reported hysterectomy; Undetermined or missing included the remaining women.

Oral contraceptives use was determined for women based on Field [2784-0.0] “*Ever taken oral contraceptive pill*”; Question: "*Have you ever taken the contraceptive pill? (include the 'mini-pill')*" as follows: Current use – Answer 1: “Yes” AND Answer -11: “*Still taking the pill”* in Field [2804-0.0] “*Age when last used oral contraceptive pill*” Question: *"How old were you when you last used the contraceptive pill?"*; Past use – Answer 1: “Yes” in Field [2784-0.0] with any other answer (not -11) or missing in Field [2804-0.0]; Never use – Answer 0: “*No*” in Field [2784-0.0]. We further re-classified to category Current use women providing Answer 5 “*Oral contraceptive pill or minipill*” to Fields [6153-0.0/3]. Women with missing information for oral contraceptive use following the above assignments were assigned the median category (Past use).

## Supplementary Table S1 Categories of self-reported medications

| Code | Name | Code | Name |
|---|---|---|---|
| **Part A: Included in the study and used for adjustment** | | | |
| **Nonsteroidal anti-inflammatory drugs (NSAID)** | | | |
| 1140856412 | norgesic tablet | 1140882268 | aspirin+codeine 300mg/8mg tablet |
| 1140861806 | aspirin 75mg tablet | 1140882392 | aspirin+codeine |
| 1140864860 | nu-seals aspirin 75mg e/c tablet | 1140910496 | propionic acid-ibuprofen |
| 1140868226 | aspirin | 1141153134 | anadin ibuprofen 200mg tablet |
| 1140868282 | aspirin+methocarbamol 325mg/400mg tablet | 1141157412 | ibuprofen product |
| 1140871310 | ibuprofen | 1141163138 | aspirin+papaveretum 500mg/7.71mg dispersible tablet |
| 1140871462 | naproxen | 1141164044 | isosorbide mononitrate+aspirin |
| 1140872040 | aspirin+metoclopramide 325mg/5mg effervescent tablet | 1141167844 | dipyridamole+aspirin |
| 1140878030 | ibuprofen+codeine phosphate | 1141184546 | ibuprofen+pseudoephedrine hydrochloride |
| 1140881612 | naproxen+misoprostol | 1141187776 | nurofen 200mg tablet |
| 1140882108 | aspirin+cyclizine hydrochloride 500mg/25mg tablet | 1141194296 | lemsip flu 12hr ibuprofen+pseudoephedrine capsule |
| 1140882190 | aspirin+glycine 500mg/133mg dispersible tablet | | |
| **Paracetamol containing medicines** | | | |
| 1140868240 | paracetamol product | 1140928954 | paracetamol+diphenhydramine hydrochloride 500mg/25mg tablet |
| 1140868274 | paracetamol+chlormezanone 450mg/100mg tablet | 1141156856 | paracetamol+domperidone 500mg/10mg tablet |
| 1140868278 | paracetamol+pentazocine 500mg/15mg tablet | 1141163764 | paracetamol+prometh hcl 120mg/1.5mg/5ml colour free s/f susp |
| 1140868322 | paracetamol+methionine 500mg/250mg tablet | 1141169026 | paracetamol+diphenhydramine hcl 120/12.5mg/5ml s/f oral soln |
| 1140872044 | paracetamol+metoclopramide 500mg/5mg tablet | 1141185058 | mandanol infant paracetamol 120mg/5ml oral suspension |
| 1140882266 | paracetamol+sodium salicylate 150mg/100mg tablet | 1141185060 | mandanol 6+ paracetamol 250mg/5ml oral suspension |
| 1140882394 | paracetamol + codeine | 1141188500 | paracetamol+pseudoephedrine hydrochloride |
| 1140882396 | paracetamol+dihydrocodeine tartrate | 1141188516 | anadin paracetamol 500mg tablet |
| 1140884404 | paracetamol+caffeine | 1141188780 | six plus parapaed paracetamol 250mg/5ml s/f oral suspension |
| 1140884408 | paracetamol+dextropropoxyphene | 1141188784 | junior parapaed paracetamol 120mg/5ml s/f oral suspension |
| 1140926016 | paracetamol+methionine 500mg/100mg tablet | 2038460150 | paracetamol |
| **Part B: Excluded from the study** | | | |
| **Antidiabetic drugs** | | | |
| 1140857494 | glibornuride | 1140874724 | daonil 5mg tablet |
| 1140857496 | glutril 25mg tablet | 1140874726 | semi-daonil 2.5mg tablet |
| 1140857500 | glymidine | 1140874728 | euglucon 2.5mg tablet |
| 1140857502 | gondafon 500mg tablet | 1140874732 | malix 2.5mg tablet |
| 1140857506 | pramidex 500mg tablet | 1140874736 | diabetamide 2.5mg tablet |
| 1140874646 | glipizide | 1140874744 | gliclazide |
| 1140874650 | glibenese 5mg tablet | 1140874746 | diamicron 80mg tablet |
| 1140874652 | minodiab 2.5mg tablet | 1140883066 | insulin product |
| 1140874664 | tolazamide | 1140884600 | metformin |
| 1140874666 | tolanase 100mg tablet | 1141152590 | glimepiride |
| 1140874674 | tolbutamide | 1141153254 | troglitazone |
| 1140874678 | glyconon 500mg tablet | 1141153262 | romozin 200mg tablet |
| 1140874680 | rastinon 500mg tablet | 1141156984 | amaryl 1mg tablet |
| 1140874686 | glucophage 500mg tablet | 1141171646 | pioglitazone |
| 1140874690 | orabet 500mg tablet | 1141171652 | actos 15mg tablet |
| 1140874706 | chlorpropamide | 1141177600 | rosiglitazone |
| 1140874712 | diabinese 100mg tablet | 1141177606 | avandia 4mg tablet |
| 1140874716 | glymese 250mg tablet | 1141189090 | rosiglitazone 1mg / metformin 500mg tablet |
| 1140874718 | glibenclamide | 1141189094 | avandamet 1mg / 500mg tablet |

| Code | Name | Code | Name |
|---|---|---|---|
| **Lipid lowering drugs** | | | |
| 1140861848 | colestid 5g/sachet granules | 1140861954 | fenofibrate |
| 1140861856 | gemfibrozil | 1140861958 | simvastatin |
| 1140861858 | lopid 300 capsule | 1140861970 | lipostat 10mg tablet |
| 1140861866 | nicofuranose | 1140862026 | ciprofibrate |
| 1140861868 | nicotinic acid product | 1140862028 | modalim 100mg tablet |
| 1140861876 | probucol | 1140888590 | colestipol |
| 1140861878 | lurselle 250mg tablet | 1140888594 | fluvastatin |
| 1140861892 | acipimox | 1140888648 | pravastatin |
| 1140861894 | olbetam 250mg capsule | 1140910632 | eptastatin |
| 1140861922 | lipid lowering drug | 1140910652 | synvinolin |
| 1140861924 | bezafibrate | 1140910654 | velastatin |
| 1140861926 | bezalip 200mg tablet | 1141146234 | atorvastatin |
| 1140861928 | bezalip-mono 400mg m/r tablet | 1141157260 | bezafibrate product |
| 1140861936 | questran 4g/sachet powder | 1141157262 | gemfibrozil product |
| 1140861942 | cholestyramine+aspartame 4g/sachet powder | 1141192410 | rosuvastatin |
| 1140861944 | clofibrate | 1141192414 | crestor 10mg tablet |
| 1140861946 | atromid-s 500mg capsule | | |
| **Glucocorticoids** | | | |
| 1140857532 | cortelan 25mg tablet | 1140874896 | hydrocortisone |
| 1140857534 | oradexon 500micrograms tablet | 1140874930 | prednisolone |
| 1140865840 | predfoam 20mg enema | 1140874936 | deltacortril enteric 2.5mg e/c tablet |
| 1140868364 | prednisone | 1140874940 | deltastab 1mg tablet |
| 1140868370 | decortisyl 5mg tablet | 1140874944 | precortisyl 1mg tablet |
| 1140868426 | triamcinolone | 1140874950 | prednesol 5mg tablet |
| 1140868434 | ledercort 2mg tablet | 1140874954 | hydrocortistab 20mg tablet |
| 1140874790 | betamethasone | 1140874956 | hydrocortone 10mg tablet |
| 1140874792 | betnelan 500mcg tablet | 1140874976 | methylprednisolone |
| 1140874794 | betnesol 500mcg soluble tablet | 1140874978 | medrone 2mg tablet |
| 1140874810 | cortistab 5mg tablet | 1140884704 | cortisone product |
| 1140874814 | cortisyl 25mg tablet | 1141157402 | prednisolone product |
| 1140874816 | dexamethasone | 1141173346 | cortisone |
| 1140874822 | decadron 500micrograms tablet | | |

Coding 4 in UK Biobank.

### Supplementary Table S2 Biomarker availability

| | Field Value | Field QC | Min | Max | Low | Reportable | High | Available |
|---|---|---|---|---|---|---|---|---|
| **MEN** | | | | | | | | |
| **Liver function tests** | | | | | | | | |
| Bilirubin T | 30840-0.0 | 30846-0.0 | 1.43 | 130.76 | 8 | 115,870 (100) | 0 | 115,878 (95.1) |
| Bilirubin D | 30660-0.0 | 30666-0.0 | 1.00 | 47.56 | 8126 | 107,807 (93.0) | 0 | 115,933 (95.1) |
| ALP | 30610-0.0 | 30616-0.0 | 15.0 | 1039.7 | 12 | 116,386 (100) | 3 | 116,401 (95.5) |
| GGT | 30730-0.0 | 30736-0.0 | 5.80 | 1101.0 | 3 | 116,334 (100) | 16 | 116,353 (95.5) |
| AST | 30650-0.0 | 30656-0.0 | 3.30 | 571.5 | 2 | 115,910 (100) | 1 | 115,913 (95.1) |
| ALT | 30620-0.0 | 30626-0.0 | 3.18 | 416.49 | 7 | 116,300 (100) | 4 | 116,311 (95.4) |
| **Metabolic biomarkers** | | | | | | | | |
| HDL-C | 30760-0.0 | 30766-0.0 | 0.347 | 4.186 | 3 | 107,421 (100) | 0 | 107,424 (88.1) |
| ApoA1 | 30630-0.0 | 30636-0.0 | 0.420 | 2.499 | 4 | 107,275 (99.9) | 98 | 107,377 (88.1) |
| LDL-C | 30780-0.0 | 30786-0.0 | 0.266 | 8.992 | 2 | 116,141 (100) | 0 | 116,143 (95.3) |
| ApoB | 30640-0.0 | 30646-0.0 | 0.401 | 2.00 | 152 | 115,620 (99.8) | 68 | 115,840 (95.0) |
| Triglycerides | 30870-0.0 | 30876-0.0 | 0.233 | 11.278 | 2 | 116,272 (100) | 50 | 116,324 (95.4) |
| HbA1c | 30750-0.0 | 30756-0.0 | 15.40 | 266.3 | 56 | 115,971 (100) | 0 | 116,027 (95.2) |
| Glucose | 30740-0.0 | 30746-0.0 | 1.542 | 36.813 | 2 | 107,344 (100) | 0 | 107,346 (88.1) |
| **Inflammatory biomarkers** | | | | | | | | |
| Lymphocytes | 30120-0.0 | - | 0.05 | 75.5 | 3 | 118,462 (100) | 0 | 118,465 (97.2) |
| Monocytes | 30130-0.0 | - | 0.01 | 10.22 | 16 | 118,449 (100) | 0 | 118,465 (97.2) |
| Neutrophils | 30140-0.0 | - | 0.02 | 25.42 | 1 | 118,464 (100) | 0 | 118,465 (97.2) |
| CRP | 30710-0.0 | 30716-0.0 | 0.08 | 79.96 | 25 | 116,122 (99.9) | 55 | 116,202 (95.3) |
| **WOMEN** | | | | | | | | |
| **Liver function tests** | | | | | | | | |
| Bilirubin T | 30840-0.0 | 30846-0.0 | 1.08 | 77.96 | 1 | 128,818 (100) | 0 | 128,819 (95.0) |
| Bilirubin D | 30660-0.0 | 30666-0.0 | 1.00 | 25.96 | 28071 | 100,866 (78.2) | 0 | 128,937 (95.1) |
| ALP | 30610-0.0 | 30616-0.0 | 10.1 | 1119.9 | 11 | 129,369 (100) | 0 | 129,380 (95.4) |
| GGT | 30730-0.0 | 30736-0.0 | 5.00 | 1132.7 | 6 | 129,311 (100) | 5 | 129,322 (95.4) |
| AST | 30650-0.0 | 30656-0.0 | 4.10 | 947.2 | 2 | 128,902 (100) | 1 | 128,905 (95.1) |
| ALT | 30620-0.0 | 30626-0.0 | 3.01 | 469.94 | 18 | 129,350 (100) | 5 | 129,373 (95.4) |
| **Metabolic biomarkers** | | | | | | | | |
| HDL-C | 30760-0.0 | 30766-0.0 | 0.228 | 4.401 | 1 | 117,436 (100) | 0 | 117,437 (86.6) |
| ApoA1 | 30630-0.0 | 30636-0.0 | 0.672 | 2.5 | 3 | 116,589 (99.5) | 576 | 117,168 (86.4) |
| LDL-C | 30780-0.0 | 30786-0.0 | 0.803 | 9.554 | 1 | 129,157 (100) | 0 | 129,158 (95.3) |
| ApoB | 30640-0.0 | 30646-0.0 | 0.400 | 2.0 | 196 | 128,943 (99.8) | 111 | 129,250 (95.3) |
| Triglycerides | 30870-0.0 | 30876-0.0 | 0.252 | 11.227 | 1 | 129,289 (100) | 4 | 129,294 (95.4) |
| HbA1c | 30750-0.0 | 30756-0.0 | 15.40 | 127.7 | 47 | 128,745 (100) | 0 | 128,792 (95.0) |
| Glucose | 30740-0.0 | 30746-0.0 | 1.798 | 24.045 | 1 | 117,357 (100) | 0 | 117,358 (86.6) |
| **Inflammatory biomarkers** | | | | | | | | |
| Lymphocytes | 30120-0.0 | - | 0.01 | 79.99 | 2 | 131,147 (100) | 0 | 131,149 (96.7) |
| Monocytes | 30130-0.0 | - | 0.01 | 12.26 | 56 | 131,093 (100) | 0 | 131,149 (96.7) |
| Neutrophils | 30140-0.0 | - | 0.01 | 18.30 | 1 | 131,148 (100) | 0 | 131,149 (96.7) |
| CRP | 30710-0.0 | 30716-0.0 | 0.08 | 79.95 | 48 | 129,149 (99.9) | 52 | 129,249 (95.3) |

**ALP** – alkaline phosphatase; **ALT** – alanine aminotransferase; **ApoA1** – apolipoprotein A1; **ApoB** – apolipoprotein B; **AST** – aspartate aminotransferase; **Bilirubin D** – direct bilirubin; **Bilirubin T** – total bilirubin; **CRP** – C-reactive protein; **HbA1c** – glycated haemoglobin; **HDL-C** – high-density-lipoprotein cholesterol; **LDL-C** – low-density-lipoprotein cholesterol.

**Field $_{QC}$** – biomarker reportability field; **Field $_{Value}$** – biomarker value field; **Min** – lowest detected value; **Max** – highest detected value; **Low** – number of participants with attempted measurements but with levels below the limit of detection (percentage from total attempted measurements per

sex); **Reported** – number of participants with reported values (percentage from total attempted measurements per sex); **High** – number of participants with attempted measurements above the upper limit of detection (percentage from total attempted measurements per sex); **Available** – number of participants with attempted measurements, comprising reported values and imputation of missing low and high levels (percentage from total in the study dataset per sex). Note that the study dataset included participants with available measurements (low, reported, or high) for at least one of the examined biomarkers.

Reportability codes:

1: "*Reportable at assay and after aliquot correction, if attempted*" – values used as provided.

2: "*Reportable at assay but not reportable after any corrections (too low)*" – see code 4.

3: "*Reportable at assay but not reportable after any corrections (too high)*" – see code 5.

4: "*Not reportable at assay (too low)*" – values were replaced with half the lowest detected level for all except direct bilirubin, which was imputed with quantile regression imputation of left-censored data (QRILC) (**imputeLCMD** v2.0 package in R), following log-transformation.

5: "*Not reportable at assay (too high)*" – replaced with the highest detected value.

Biomarker values for participants without attempted measurements were considered missing and these participants were excluded from the analysis of the corresponding biomarker.

## Supplementary Table S3 Characteristics of study participants by sex and body shape phenotypes

| | MEN | | | | | WOMEN | | | | |
|---|---|---|---|---|---|---|---|---|---|---|
| | Overall | Pear | Slim | Wide | Apple | Overall | Pear | Slim | Wide | Apple |
| Cohort size: n (% per sex) | 121,879 | 32,736 (26.9) | 37,383 (30.7) | 32,504 (26.7) | 19,256 (15.8) | 135,559 | 35,985 (26.5) | 31,054 (22.9) | 39,632 (29.2) | 28,888 (21.3) |
| Anthropometry: mean (SD) | | | | | | | | | | |
| Height (cm) | 176.5 (6.7) | 176.5 (6.7) | 176.1 (6.6) | 176.9 (6.8) | 176.7 (6.7) | 163.1 (6.2) | 163.0 (6.2) | 163.0 (6.2) | 162.9 (6.3) | 163.5 (6.1) |
| Weight (kg) | 84.6 (13) | 83.5 (13.1) | 84.4 (12.4) | 84.8 (13.6) | 86.2 (12.9) | 69.8 (12.4) | 68.4 (12.3) | 68.6 (11.6) | 70.6 (13.2) | 71.7 (11.8) |
| Waist circumference (cm) | 94.8 (10.2) | 91.3 (9.3) | 91.2 (8.8) | 99.4 (9.9) | 100.1 (9.3) | 82.5 (11.1) | 77.3 (9.1) | 77.0 (8.6) | 87.5 (11.2) | 88.0 (9.8) |
| Hip circumference (cm) | 102.7 (6.8) | 104.3 (6.5) | 99.7 (5.9) | 105.4 (6.8) | 101.0 (5.9) | 102.2 (9.1) | 103.7 (9.0) | 97.9 (7.7) | 105.5 (9.7) | 100.2 (7.5) |
| Waist-to-hip ratio | 0.92 (0.06) | 0.87 (0.05) | 0.91 (0.05) | 0.94 (0.05) | 0.99 (0.05) | 0.81 (0.07) | 0.74 (0.04) | 0.79 (0.04) | 0.83 (0.05) | 0.88 (0.05) |
| BMI category: n (%) | | | | | | | | | | |
| Normal weight: BMI≥18.5 to <25 | 36,310 (29.8) | 11,164 (34.1) | 10,187 (27.3) | 10,316 (31.7) | 4643 (24.1) | 61,755 (45.6) | 18,681 (51.9) | 15,355 (49.4) | 17,127 (43.2) | 10,592 (36.7) |
| Overweight: BMI≥25 to <30 | 62,024 (50.9) | 15,912 (48.6) | 20,029 (53.6) | 15,760 (48.5) | 10,323 (53.6) | 49,574 (36.6) | 11,813 (32.8) | 11,255 (36.2) | 14,007 (35.3) | 12,499 (43.3) |
| Obese: BMI≥30 to <45 | 23,545 (19.3) | 5660 (17.3) | 7167 (19.2) | 6428 (19.8) | 4290 (22.3) | 24,230 (17.9) | 5491 (15.3) | 4444 (14.3) | 8498 (21.4) | 5797 (20.1) |
| Weight change, last year: n (%) | | | | | | | | | | |
| Lost weight | 15,788 (13.0) | 4452 (13.6) | 5483 (14.7) | 3524 (10.8) | 2329 (12.1) | 18,929 (14.0) | 4585 (12.7) | 4845 (15.6) | 5115 (12.9) | 4384 (15.2) |
| Stable weight | 77,851 (63.9) | 21,547 (65.8) | 23,919 (64.0) | 20,671 (63.6) | 11,714 (60.8) | 71,542 (52.8) | 19,726 (54.8) | 16,788 (54.1) | 20,508 (51.7) | 14,520 (50.3) |
| Gained weight | 26,017 (21.3) | 6227 (19.0) | 7358 (19.7) | 7627 (23.5) | 4805 (25.0) | 42,870 (31.6) | 11,108 (30.9) | 8957 (28.8) | 13,288 (33.5) | 9517 (32.9) |
| Missing | 2223 (1.8) | 510 (1.6) | 623 (1.7) | 682 (2.1) | 408 (2.1) | 2218 (1.6) | 566 (1.6) | 464 (1.5) | 721 (1.8) | 467 (1.6) |
| Smoking status: n (%) | | | | | | | | | | |
| Never smoked | 45,279 (37.2) | 13,193 (40.3) | 14,479 (38.7) | 11,328 (34.9) | 6279 (32.6) | 60,518 (44.6) | 17,547 (48.8) | 14,010 (45.1) | 17,404 (43.9) | 11,557 (40.0) |
| Former occasional smoker | 33,394 (27.4) | 9814 (30.0) | 10,163 (27.2) | 8796 (27.1) | 4621 (24.0) | 38,740 (28.6) | 10,549 (29.3) | 8843 (28.5) | 11,433 (28.8) | 7915 (27.4) |
| Former regular smoker | 27,727 (22.7) | 6385 (19.5) | 7936 (21.2) | 8193 (25.2) | 5213 (27.1) | 24,374 (18.0) | 5616 (15.6) | 5376 (17.3) | 7333 (18.5) | 6049 (20.9) |
| Current smoker | 15,161 (12.4) | 3272 (10.0) | 4698 (12.6) | 4096 (12.6) | 3095 (16.1) | 11,567 (8.5) | 2186 (6.1) | 2759 (8.9) | 3350 (8.5) | 3272 (11.3) |
| Missing | 318 (0.3) | 72 (0.2) | 107 (0.3) | 91 (0.3) | 48 (0.2) | 360 (0.3) | 87 (0.2) | 66 (0.2) | 112 (0.3) | 95 (0.3) |
| Alcohol intake: n (%) | | | | | | | | | | |
| Up to three times a month | 23,157 (19.0) | 6095 (18.6) | 7111 (19.0) | 6169 (19.0) | 3782 (19.6) | 43,504 (32.1) | 11,581 (32.2) | 9694 (31.2) | 13,127 (33.1) | 9102 (31.5) |
| Up to four times a week | 67,421 (55.3) | 18,647 (57.0) | 21,733 (58.1) | 17,098 (52.6) | 9943 (51.6) | 68,478 (50.5) | 18,592 (51.7) | 16,223 (52.2) | 19,356 (48.8) | 14,307 (49.5) |
| Daily | 31,228 (25.6) | 7974 (24.4) | 8521 (22.8) | 9217 (28.4) | 5516 (28.6) | 23,507 (17.3) | 5798 (16.1) | 5122 (16.5) | 7125 (18.0) | 5462 (18.9) |
| Missing | 73 (0.1) | 20 (0.1) | 18 (0.0) | 20 (0.1) | 15 (0.1) | 70 (0.1) | 14 (0.0) | 15 (0.0) | 24 (0.1) | 17 (0.1) |
| Physical activity: n (%) | | | | | | | | | | |
| Inactive | 16,443 (13.5) | 3685 (11.3) | 3908 (10.5) | 5692 (17.5) | 3158 (16.4) | 21,081 (15.6) | 5387 (15.0) | 4083 (13.1) | 7009 (17.7) | 4602 (15.9) |
| Moderately active | 52,282 (42.9) | 13,427 (41.0) | 14,470 (38.7) | 15,530 (47.8) | 8855 (46.0) | 69,967 (51.6) | 18,480 (51.4) | 15,145 (48.8) | 21,275 (53.7) | 15,067 (52.2) |
| Active | 52,892 (43.4) | 15,576 (47.6) | 18,932 (50.6) | 11,194 (34.4) | 7190 (37.3) | 44,150 (32.6) | 12,050 (33.5) | 11,769 (37.9) | 11,198 (28.3) | 9133 (31.6) |
| Missing | 262 (0.2) | 48 (0.1) | 73 (0.2) | 88 (0.3) | 53 (0.3) | 361 (0.3) | 68 (0.2) | 57 (0.2) | 150 (0.4) | 86 (0.3) |
| Townsend index | | | | | | | | | | |
| Mean (SD) | -1.55 (2.96) | -1.71 (2.87) | -1.51 (2.98) | -1.55 (2.97) | -1.32 (3.07) | -1.63 (2.86) | -1.80 (2.75) | -1.67 (2.87) | -1.56 (2.88) | -1.47 (2.95) |
| Missing: n (%) | 150 (0.1) | 49 (0.1) | 48 (0.1) | 31 (0.1) | 22 (0.1) | 151 (0.1) | 38 (0.1) | 41 (0.1) | 41 (0.1) | 31 (0.1) |
| Time of sample collection: n (%) | | | | | | | | | | |
| Morning: ≥8 am to <12 am | 33,871 (27.8) | 9716 (29.7) | 10,763 (28.8) | 8430 (25.9) | 4962 (25.8) | 34,783 (25.7) | 10,012 (27.8) | 8326 (26.8) | 9375 (23.7) | 7070 (24.5) |
| Afternoon: ≥12 am to <4 pm | 44,251 (36.3) | 11,495 (35.1) | 13,055 (34.9) | 12,512 (38.5) | 7189 (37.3) | 56,067 (41.4) | 14,681 (40.8) | 12,163 (39.2) | 17,216 (43.4) | 12,007 (41.6) |
| Evening: ≥4 pm to ≤8:15 pm | 43,656 (35.8) | 11,488 (35.1) | 13,547 (36.2) | 11,531 (35.5) | 7090 (36.8) | 44,558 (32.9) | 11,254 (31.3) | 10,545 (34.0) | 12,978 (32.7) | 9781 (33.9) |
| Missing | 101 (0.1) | 37 (0.1) | 18 (<0.0) | 31 (0.1) | 15 (0.1) | 151 (0.1) | 38 (0.1) | 20 (0.1) | 63 (0.2) | 30 (0.1) |

| | MEN | | | | | WOMEN | | | | |
|---|---|---|---|---|---|---|---|---|---|---|
| | **Overall** | **Pear** | **Slim** | **Wide** | **Apple** | **Overall** | **Pear** | **Slim** | **Wide** | **Apple** |
| Fasting time: n (%) | | | | | | | | | | |
| 0-2 hours | 33,771 (27.7) | 9319 (28.5) | 10,750 (28.8) | 8512 (26.2) | 5190 (27.0) | 36,840 (27.2) | 9918 (27.6) | 8795 (28.3) | 10320 (26.0) | 7807 (27.0) |
| 3-4 hours | 59,352 (48.7) | 15,801 (48.3) | 17,945 (48.0) | 16,222 (49.9) | 9384 (48.7) | 70,446 (52.0) | 18,721 (52.0) | 15,904 (51.2) | 20,822 (52.5) | 14,999 (51.9) |
| ≥5 hours | 28,754 (23.6) | 7615 (23.3) | 8687 (23.2) | 7770 (23.9) | 4682 (24.3) | 28,268 (20.9) | 7343 (20.4) | 6354 (20.5) | 8490 (21.4) | 6081 (21.1) |
| Missing | 2 | 1 | 1 | - | - | 5 | 3 | 1 | - | 1 |
| NSAID: n (%) | | | | | | | | | | |
| No | 97,571 (80.1) | 26,599 (81.3) | 29,804 (79.7) | 25,957 (79.9) | 15,211 (79.0) | 104,066 (76.8) | 27,618 (76.7) | 23,843 (76.8) | 30,458 (76.9) | 22,147 (76.7) |
| Yes | 23,126 (19.0) | 5878 (18.0) | 7218 (19.3) | 6220 (19.1) | 3810 (19.8) | 30,504 (22.5) | 8124 (22.6) | 6990 (22.5) | 8853 (22.3) | 6537 (22.6) |
| Missing | 1182 (1.0) | 259 (0.8) | 361 (1.0) | 327 (1.0) | 235 (1.2) | 989 (0.7) | 243 (0.7) | 221 (0.7) | 321 (0.8) | 204 (0.7) |
| Paracetamol: n (%) | | | | | | | | | | |
| No | 100,676 (82.6) | 27,541 (84.1) | 31,028 (83) | 26,570 (81.7) | 15,537 (80.7) | 100,142 (73.9) | 26,878 (74.7) | 23,193 (74.7) | 28,859 (72.8) | 21,212 (73.4) |
| Yes | 20,009 (16.4) | 4935 (15.1) | 5992 (16) | 5604 (17.2) | 3478 (18.1) | 34,410 (25.4) | 8858 (24.6) | 7640 (24.6) | 10444 (26.4) | 7468 (25.9) |
| Missing | 1194 (1.0) | 260 (0.8) | 363 (1.0) | 330 (1.0) | 241 (1.3) | 1007 (0.7) | 249 (0.7) | 221 (0.7) | 329 (0.8) | 208 (0.7) |
| Assessment region: n (%) | | | | | | | | | | |
| London | 13,950 (11.4) | 3706 (11.3) | 4277 (11.4) | 3612 (11.1) | 2355 (12.2) | 16,469 (12.1) | 4172 (11.6) | 3650 (11.8) | 4833 (12.2) | 3814 (13.2) |
| North-West | 18,273 (15.0) | 4579 (14.0) | 7105 (19.0) | 3660 (11.3) | 2929 (15.2) | 18,974 (14.0) | 4580 (12.7) | 5578 (18.0) | 4498 (11.3) | 4318 (14.9) |
| North-East | 14,557 (11.9) | 3714 (11.3) | 5368 (14.4) | 3387 (10.4) | 2088 (10.8) | 16,136 (11.9) | 4343 (12.1) | 3934 (12.7) | 4307 (10.9) | 3552 (12.3) |
| Yorkshire and Humber | 18,609 (15.3) | 4804 (14.7) | 5180 (13.9) | 5337 (16.4) | 3288 (17.1) | 21,084 (15.6) | 5990 (16.6) | 4339 (14.0) | 6340 (16.0) | 4415 (15.3) |
| West Midlands | 11,114 (9.1) | 2343 (7.2) | 3763 (10.1) | 3111 (9.6) | 1897 (9.9) | 10,928 (8.1) | 2351 (6.5) | 3094 (10.0) | 3168 (8.0) | 2315 (8.0) |
| East Midlands | 8549 (7.0) | 2598 (7.9) | 1841 (4.9) | 2711 (8.3) | 1399 (7.3) | 9738 (7.2) | 2911 (8.1) | 1188 (3.8) | 3397 (8.6) | 2242 (7.8) |
| South-East | 11,411 (9.4) | 3616 (11.0) | 1682 (4.5) | 4474 (13.8) | 1639 (8.5) | 12,840 (9.5) | 3823 (10.6) | 1469 (4.7) | 5189 (13.1) | 2359 (8.2) |
| South-West | 11,254 (9.2) | 3417 (10.4) | 3235 (8.7) | 2876 (8.8) | 1726 (9.0) | 13,050 (9.6) | 3582 (10.0) | 3168 (10.2) | 3602 (9.1) | 2698 (9.3) |
| Wales | 5122 (4.2) | 1394 (4.3) | 1449 (3.9) | 1492 (4.6) | 787 (4.1) | 5651 (4.2) | 1352 (3.8) | 1271 (4.1) | 1872 (4.7) | 1156 (4.0) |
| Scotland | 9040 (7.4) | 2565 (7.8) | 3483 (9.3) | 1844 (5.7) | 1148 (6.0) | 10,689 (7.9) | 2881 (8.0) | 3363 (10.8) | 2426 (6.1) | 2019 (7.0) |
| Menopausal status: n (%) | | | | | | | | | | |
| Pre-menopausal | | | | | | 40,814 (30.1) | 12,269 (34.1) | 11,380 (36.6) | 9381 (23.7) | 7784 (26.9) |
| Post-menopausal | | | | | | 81,551 (60.2) | 20,234 (56.2) | 16,659 (53.6) | 26,458 (66.8) | 18,200 (63.0) |
| Undetermined or missing | | | | | | 13,194 (9.7) | 3482 (9.7) | 3015 (9.7) | 3793 (9.6) | 2904 (10.1) |
| HRT use: n (%) | | | | | | | | | | |
| Never | | | | | | 96,512 (71.2) | 26,684 (74.2) | 23,453 (75.5) | 26,508 (66.9) | 19,867 (68.8) |
| Past | | | | | | 38,714 (28.6) | 9224 (25.6) | 7528 (24.2) | 13,019 (32.8) | 8943 (31.0) |
| Missing | | | | | | 333 (0.2) | 77 (0.2) | 73 (0.2) | 105 (0.3) | 78 (0.3) |
| OC use: n (%) | | | | | | | | | | |
| Never | | | | | | 21,519 (15.9) | 5491 (15.3) | 4459 (14.4) | 7003 (17.7) | 4566 (15.8) |
| Past | | | | | | 113,782 (83.9) | 30,422 (84.5) | 26,545 (85.5) | 32,544 (82.1) | 24,271 (84.0) |
| Missing | | | | | | 258 (0.2) | 72 (0.2) | 50 (0.2) | 85 (0.2) | 51 (0.2) |
| Age at the last live birth: n (%) | | | | | | | | | | |
| No live births | | | | | | 25,650 (18.9) | 7504 (20.9) | 6336 (20.4) | 7047 (17.8) | 4763 (16.5) |
| < 30 years | | | | | | 48,308 (35.6) | 12,479 (34.7) | 10,710 (34.5) | 14,442 (36.4) | 10,677 (37.0) |
| ≥ 30 years | | | | | | 61,349 (45.3) | 15,960 (44.4) | 13,961 (45.0) | 18,047 (45.5) | 13,381 (46.3) |
| Missing | | | | | | 252 (0.2) | 42 (0.1) | 47 (0.2) | 96 (0.2) | 67 (0.2) |

**ABSI** – a body shape index (cut-offs ≥80 for men, ≥73 for women); **Apple** – large-ABSI-small-HI; **BMI** – body mass index; **HRT** – hormone replacement therapy; **HI** – hip index (cut-offs ≥49 for men, ≥64 for women); **NSAID** – nonsteroidal anti-inflammatory drugs; **OC** – oral contraceptives; **Pear** – small-ABSI-large-HI; **SD** – standard deviation; **Slim** – small-ABSI-small-HI; **Wide** – large-ABSI-large-HI; **n (%)** – number (percentage from total per column).

**Supplementary Table S4 Linear and non-linear associations of biomarkers with body size and body shape indices (continuous)**

| | | MEN | | | | WOMEN | | | |
|---|---|---|---|---|---|---|---|---|---|
| | | **Count** | **BMI** | **ABSI** | **HI** | **Count** | **BMI** | **ABSI** | **HI** |
| | | | (per one SD) | (per one SD) | (per one SD) | | (per one SD) | (per one SD) | (per one SD) |
| **Liver function tests** | | | | | | | | | |
| Bilirubin total | LIN | 115,878 | -0.06 (-0.07 to -0.06)** | -0.06 (-0.06 to -0.05)** | 0.02 (0.01 to 0.02)** | 128,819 | -0.11 (-0.11 to -0.10)** | -0.06 (-0.06 to -0.05)** | 0.01 (0.01 to 0.02)* |
| | SB1 | | -0.05 (-0.07 to -0.03)** | -0.06 (-0.08 to -0.05)** | 0.03 (0.02 to 0.04)* | | -0.19 (-0.21 to -0.17)** | -0.09 (-0.10 to -0.08)** | 0.01 (0.00 to 0.02) |
| | SB2 | | -0.01 (-0.03 to 0.00) | 0.01 (-0.01 to 0.02) | -0.02 (-0.03 to 0.00) | | 0.09 (0.07 to 0.10)** | 0.04 (0.03 to 0.06)** | 0.00 (-0.01 to 0.02) |
| p non-linearity | | | 0.106 | 0.464 | 0.020 | | **$1*10^{-21}$** | **$2*10^{-7}$** | 0.547 |
| Bilirubin direct | LIN | 115,933 | -0.07 (-0.07 to -0.06)** | -0.06 (-0.07 to -0.06)** | 0.03 (0.03 to 0.04)** | 128,937 | -0.10 (-0.11 to -0.10)** | -0.08 (-0.08 to -0.07)** | 0.03 (0.03 to 0.04)** |
| | SB1 | | -0.14 (-0.15 to -0.12)** | -0.09 (-0.11 to -0.08)** | 0.03 (0.02 to 0.05)** | | -0.24 (-0.26 to -0.22)** | -0.13 (-0.14 to -0.11)** | 0.02 (0.01 to 0.04) |
| | SB2 | | 0.08 (0.06 to 0.09)** | 0.04 (0.03 to 0.06)** | 0.00 (-0.02 to 0.01) | | 0.15 (0.13 to 0.16)** | 0.06 (0.05 to 0.08)** | 0.01 (0.00 to 0.03) |
| p non-linearity | | | **$3*10^{-19}$** | **$4*10^{-7}$** | 0.901 | | **$2*10^{-60}$** | **$4*10^{-15}$** | 0.070 |
| ALP | LIN | 116,401 | 0.06 (0.05 to 0.07)** | 0.09 (0.08 to 0.10)** | -0.02 (-0.03 to -0.01)** | 129,380 | 0.17 (0.17 to 0.18)** | 0.08 (0.07 to 0.08)** | 0.01 (0.00 to 0.01) |
| | SB1 | | 0.07 (0.05 to 0.08)** | 0.07 (0.06 to 0.09)** | -0.04 (-0.05 to -0.02)** | | 0.25 (0.24 to 0.27)** | 0.09 (0.07 to 0.10)** | 0.00 (-0.01 to 0.01) |
| | SB2 | | -0.01 (-0.02 to 0.01) | 0.02 (0.01 to 0.04) | 0.02 (0.01 to 0.04) | | -0.08 (-0.10 to -0.07)** | -0.01 (-0.03 to 0.00) | 0.01 (0.00 to 0.02) |
| p non-linearity | | | 0.382 | 0.006 | 0.003 | | **$5*10^{-24}$** | 0.053 | 0.155 |
| GGT | LIN | 116,353 | 0.26 (0.25 to 0.26)** | 0.12 (0.11 to 0.12)** | -0.07 (-0.08 to -0.06)** | 129,322 | 0.22 (0.21 to 0.22)** | 0.12 (0.11 to 0.12)** | -0.06 (-0.07 to -0.06)** |
| | SB1 | | 0.38 (0.36 to 0.40)** | 0.13 (0.11 to 0.14)** | -0.06 (-0.07 to -0.04)** | | 0.13 (0.11 to 0.15)** | 0.14 (0.13 to 0.15)** | -0.09 (-0.10 to -0.07)** |
| | SB2 | | -0.14 (-0.15 to -0.12)** | -0.01 (-0.03 to 0.00) | -0.02 (-0.03 to 0.00) | | 0.09 (0.08 to 0.11)** | -0.03 (-0.04 to -0.01) | 0.03 (0.02 to 0.05)* |
| p non-linearity | | | **$2*10^{-60}$** | 0.168 | 0.021 | | **$4*10^{-27}$** | $4*10^{-4}$ | **$9*10^{-6}$** |
| AST | LIN | 115,913 | 0.15 (0.15 to 0.16)** | 0.02 (0.01 to 0.02)** | -0.04 (-0.04 to -0.03)** | 128,905 | 0.04 (0.03 to 0.04)** | 0.04 (0.04 to 0.05)** | -0.04 (-0.05 to -0.03)** |
| | SB1 | | 0.10 (0.08 to 0.12)** | -0.02 (-0.03 to -0.01) | -0.03 (-0.04 to -0.02)* | | -0.12 (-0.14 to -0.10)** | 0.02 (0.01 to 0.04) | -0.06 (-0.07 to -0.05)** |
| | SB2 | | 0.06 (0.04 to 0.08)** | 0.05 (0.03 to 0.06)** | -0.01 (-0.02 to 0.01) | | 0.17 (0.15 to 0.18)** | 0.02 (0.01 to 0.04) | 0.03 (0.01 to 0.04) |
| p non-linearity | | | **$2*10^{-11}$** | **$2*10^{-8}$** | 0.318 | | **$5*10^{-75}$** | 0.002 | $1*10^{-4}$ |
| ALT | LIN | 116,311 | 0.33 (0.33 to 0.34)** | 0.10 (0.09 to 0.10)** | -0.08 (-0.09 to -0.08)** | 129,373 | 0.23 (0.22 to 0.23)** | 0.12 (0.11 to 0.13)** | -0.08 (-0.09 to -0.08)** |
| | SB1 | | 0.46 (0.44 to 0.47)** | 0.13 (0.12 to 0.14)** | -0.06 (-0.07 to -0.04)** | | 0.18 (0.16 to 0.20)** | 0.15 (0.14 to 0.17)** | -0.10 (-0.11 to -0.09)** |
| | SB2 | | -0.14 (-0.16 to -0.13)** | -0.04 (-0.06 to -0.03)** | -0.03 (-0.05 to -0.02)* | | 0.05 (0.03 to 0.07)** | -0.04 (-0.05 to -0.02)** | 0.02 (0.01 to 0.03) |
| p non-linearity | | | **$7*10^{-70}$** | **$1*10^{-7}$** | **$2*10^{-6}$** | | **$1*10^{-8}$** | **$6*10^{-7}$** | 0.007 |
| **Metabolic biomarkers** | | | | | | | | | |
| HDL-C | LIN | 107,424 | -0.30 (-0.31 to -0.30)** | -0.08 (-0.09 to -0.07)** | 0.08 (0.08 to 0.09)** | 117,437 | -0.30 (-0.30 to -0.29)** | -0.16 (-0.16 to -0.15)** | 0.11 (0.11 to 0.12)** |
| | SB1 | | -0.53 (-0.55 to -0.52)** | -0.14 (-0.16 to -0.13)** | 0.06 (0.05 to 0.07)** | | -0.43 (-0.45 to -0.41)** | -0.23 (-0.24 to -0.21)** | 0.11 (0.10 to 0.12)** |
| | SB2 | | 0.26 (0.24 to 0.28)** | 0.09 (0.07 to 0.10)** | 0.03 (0.02 to 0.05)* | | 0.14 (0.12 to 0.15)** | 0.09 (0.07 to 0.10)** | 0.01 (0.00 to 0.02) |
| p non-linearity | | | **$3*10^{-216}$** | **$4*10^{-26}$** | **$3*10^{-5}$** | | **$4*10^{-58}$** | **$9*10^{-31}$** | 0.118 |

| | | MEN | | | | WOMEN | | | |
|---|---|---|---|---|---|---|---|---|---|
| | | **Count** | **BMI** | **ABSI** | **HI** | **Count** | **BMI** | **ABSI** | **HI** |
| | | | (per one SD) | (per one SD) | (per one SD) | | (per one SD) | (per one SD) | (per one SD) |
| **Metabolic biomarkers** *(continued)* | | | | | | | | | |
| ApoA1 | LIN | 107,377 | -0.23 (-0.24 to -0.22)** | -0.05 (-0.06 to -0.05)** | 0.05 (0.05 to 0.06)** | 117,168 | -0.22 (-0.23 to -0.22)** | -0.09 (-0.10 to -0.09)** | 0.08 (0.07 to 0.08)** |
| | SB1 | | -0.42 (-0.44 to -0.41)** | -0.10 (-0.11 to -0.09)** | 0.03 (0.02 to 0.05)** | | -0.36 (-0.38 to -0.34)** | -0.14 (-0.15 to -0.12)** | 0.07 (0.06 to 0.08)** |
| | SB2 | | 0.21 (0.20 to 0.23)** | 0.07 (0.05 to 0.08)** | 0.03 (0.01 to 0.04) | | 0.14 (0.12 to 0.16)** | 0.05 (0.04 to 0.07)** | 0.01 (0.00 to 0.03) |
| p non-linearity | | | **$3*10^{-142}$** | **$5*10^{-16}$** | $3*10^{-4}$ | | **$2*10^{-58}$** | **$2*10^{-11}$** | 0.057 |
| LDL-C | LIN | 116,143 | 0.12 (0.12 to 0.13)** | 0.06 (0.06 to 0.07)** | -0.04 (-0.05 to -0.04)** | 129,158 | 0.14 (0.14 to 0.15)** | 0.11 (0.11 to 0.12)** | -0.08 (-0.08 to -0.07)** |
| | SB1 | | 0.42 (0.41 to 0.44)** | 0.13 (0.12 to 0.14)** | 0.00 (-0.01 to 0.01) | | 0.39 (0.38 to 0.41)** | 0.18 (0.17 to 0.19)** | -0.05 (-0.06 to -0.04)** |
| | SB2 | | -0.34 (-0.35 to -0.32)** | -0.09 (-0.11 to -0.07)** | -0.06 (-0.07 to -0.04)** | | -0.26 (-0.28 to -0.25)** | -0.09 (-0.10 to -0.07)** | -0.04 (-0.05 to -0.02)** |
| p non-linearity | | | **$<1*10^{-317}$** | **$3*10^{-26}$** | **$3*10^{-13}$** | | **$2*10^{-216}$** | **$3*10^{-31}$** | **$2*10^{-7}$** |
| ApoB | LIN | 115,840 | 0.17 (0.16 to 0.17)** | 0.07 (0.07 to 0.08)** | -0.06 (-0.06 to -0.05)** | 129,250 | 0.17 (0.16 to 0.17)** | 0.13 (0.12 to 0.13)** | -0.09 (-0.09 to -0.08)** |
| | SB1 | | 0.46 (0.44 to 0.48)** | 0.14 (0.13 to 0.15)** | -0.01 (-0.03 to 0.00) | | 0.40 (0.38 to 0.41)** | 0.20 (0.18 to 0.21)** | -0.07 (-0.08 to -0.06)** |
| | SB2 | | -0.33 (-0.35 to -0.31)** | -0.09 (-0.11 to -0.07)** | -0.06 (-0.07 to -0.04)** | | -0.24 (-0.26 to -0.22)** | -0.09 (-0.10 to -0.07)** | -0.03 (-0.04 to -0.01)* |
| p non-linearity | | | **$1*10^{-317}$** | **$2*10^{-26}$** | **$6*10^{-14}$** | | **$1*10^{-178}$** | **$2*10^{-29}$** | **$5*10^{-5}$** |
| Triglycerides | LIN | 116,324 | 0.31 (0.30 to 0.32)** | 0.13 (0.12 to 0.13)** | -0.11 (-0.12 to -0.11)** | 129,294 | 0.30 (0.29 to 0.30)** | 0.22 (0.22 to 0.23)** | -0.14 (-0.15 to -0.14)** |
| | SB1 | | 0.60 (0.59 to 0.62)** | 0.20 (0.19 to 0.21)** | -0.08 (-0.09 to -0.07)** | | 0.47 (0.46 to 0.49)** | 0.32 (0.31 to 0.33)** | -0.14 (-0.15 to -0.13)** |
| | SB2 | | -0.33 (-0.35 to -0.31)** | -0.10 (-0.12 to -0.08)** | -0.05 (-0.06 to -0.03)** | | -0.19 (-0.20 to -0.17)** | -0.12 (-0.13 to -0.11)** | 0.00 (-0.02 to 0.01) |
| p non-linearity | | | **$1*10^{-317}$** | **$3*10^{-36}$** | **$2*10^{-10}$** | | **$4*10^{-122}$** | **$6*10^{-64}$** | 0.672 |
| HbA1c | LIN | 116,027 | 0.15 (0.14 to 0.15)** | 0.05 (0.04 to 0.05)** | -0.03 (-0.04 to -0.03)** | 128,792 | 0.13 (0.13 to 0.14)** | 0.07 (0.07 to 0.08)** | -0.05 (-0.05 to -0.04)** |
| | SB1 | | 0.01 (0.00 to 0.03) | 0.03 (0.01 to 0.04)* | -0.05 (-0.07 to -0.04)** | | -0.02 (-0.04 to 0.00) | 0.10 (0.09 to 0.12)** | -0.05 (-0.06 to -0.04)** |
| | SB2 | | 0.15 (0.13 to 0.17)** | 0.03 (0.01 to 0.04) | 0.03 (0.01 to 0.04) | | 0.16 (0.14 to 0.17)** | -0.04 (-0.05 to -0.02)** | 0.00 (-0.01 to 0.02) |
| p non-linearity | | | **$4*10^{-66}$** | 0.001 | $2*10^{-4}$ | | **$1*10^{-74}$** | **$3*10^{-7}$** | 0.687 |
| Glucose | LIN | 107,346 | 0.12 (0.11 to 0.12)** | 0.03 (0.03 to 0.04)** | -0.04 (-0.04 to -0.03)** | 117,358 | 0.11 (0.11 to 0.12)** | 0.04 (0.03 to 0.04)** | -0.05 (-0.05 to -0.04)** |
| | SB1 | | 0.07 (0.06 to 0.09)** | 0.01 (-0.01 to 0.02) | -0.04 (-0.05 to -0.03)** | | 0.08 (0.06 to 0.10)** | 0.03 (0.02 to 0.05)* | -0.05 (-0.06 to -0.04)** |
| | SB2 | | 0.05 (0.03 to 0.06)** | 0.03 (0.01 to 0.05) | 0.00 (-0.01 to 0.02) | | 0.03 (0.01 to 0.05) | 0.00 (-0.01 to 0.02) | 0.00 (-0.01 to 0.02) |
| p non-linearity | | | **$3*10^{-7}$** | $7*10^{-4}$ | 0.849 | | $5*10^{-4}$ | 0.782 | 0.580 |
| **Inflammatory biomarkers** | | | | | | | | | |
| Lymphocytes | LIN | 118,465 | 0.17 (0.16 to 0.17)** | 0.03 (0.03 to 0.04)** | -0.02 (-0.03 to -0.02)** | 131,149 | 0.13 (0.13 to 0.14)** | 0.05 (0.05 to 0.06)** | -0.04 (-0.04 to -0.03)** |
| | SB1 | | 0.24 (0.22 to 0.25)** | 0.05 (0.04 to 0.06)** | -0.01 (-0.02 to 0.00) | | 0.20 (0.19 to 0.22)** | 0.07 (0.06 to 0.09)** | -0.04 (-0.05 to -0.02)** |
| | SB2 | | -0.08 (-0.10 to -0.06)** | -0.02 (-0.04 to -0.01) | -0.02 (-0.03 to 0.00) | | -0.07 (-0.09 to -0.06)** | -0.03 (-0.04 to -0.01) | 0.00 (-0.02 to 0.01) |
| p non-linearity | | | **$7*10^{-22}$** | 0.006 | 0.039 | | **$1*10^{-17}$** | $2*10^{-4}$ | 0.566 |

| | | MEN | | | | WOMEN | | | |
|---|---|---|---|---|---|---|---|---|---|
| | | Count | BMI (per one SD) | ABSI (per one SD) | HI (per one SD) | Count | BMI (per one SD) | ABSI (per one SD) | HI (per one SD) |
| **Inflammatory biomarkers** *(continued)* | | | | | | | | | |
| Monocytes | LIN | 118,465 | 0.12 (0.12 to 0.13)** | 0.06 (0.06 to 0.07)** | -0.03 (-0.03 to -0.02)** | 131,149 | 0.07 (0.07 to 0.08)** | 0.04 (0.03 to 0.04)** | -0.01 (-0.01 to 0.00) |
| | SB1 | | 0.11 (0.09 to 0.13)** | 0.06 (0.05 to 0.08)** | -0.02 (-0.03 to -0.01) | | 0.07 (0.05 to 0.09)** | 0.04 (0.02 to 0.05)** | -0.02 (-0.03 to -0.01) |
| | SB2 | | 0.01 (-0.01 to 0.03) | 0.00 (-0.02 to 0.01) | -0.01 (-0.02 to 0.01) | | 0.00 (-0.01 to 0.02) | 0.00 (-0.01 to 0.02) | 0.01 (0.00 to 0.03) |
| $p_{\text{non-linearity}}$ | | | 0.170 | 0.874 | 0.336 | | 0.586 | 0.912 | 0.109 |
| Neutrophils | LIN | 118,465 | 0.09 (0.08 to 0.09)** | 0.09 (0.08 to 0.09)** | -0.03 (-0.03 to -0.02)** | 131,149 | 0.12 (0.12 to 0.13)** | 0.06 (0.06 to 0.07)** | 0.00 (-0.01 to 0.00) |
| | SB1 | | 0.05 (0.03 to 0.06)** | 0.08 (0.06 to 0.09)** | -0.03 (-0.05 to -0.02)** | | 0.08 (0.06 to 0.09)** | 0.08 (0.06 to 0.09)** | -0.02 (-0.03 to -0.01) |
| | SB2 | | 0.04 (0.03 to 0.06)** | 0.02 (0.00 to 0.03) | 0.01 (-0.01 to 0.02) | | 0.05 (0.03 to 0.07)** | -0.02 (-0.03 to 0.00) | 0.02 (0.00 to 0.03) |
| $p_{\text{non-linearity}}$ | | | **$4*10^{-7}$** | 0.057 | 0.250 | | **$5*10^{-9}$** | 0.022 | 0.013 |
| CRP | LIN | 116,202 | 0.33 (0.32 to 0.33)** | 0.14 (0.13 to 0.14)** | -0.04 (-0.05 to -0.04)** | 129,249 | 0.47 (0.46 to 0.47)** | 0.11 (0.11 to 0.12)** | -0.02 (-0.02 to -0.01)** |
| | SB1 | | 0.43 (0.42 to 0.45)** | 0.13 (0.12 to 0.14)** | -0.06 (-0.07 to -0.05)** | | 0.57 (0.55 to 0.58)** | 0.14 (0.12 to 0.15)** | -0.03 (-0.04 to -0.02)** |
| | SB2 | | -0.12 (-0.13 to -0.10)** | 0.01 (-0.01 to 0.02) | 0.02 (0.01 to 0.04) | | -0.10 (-0.12 to -0.09)** | -0.03 (-0.05 to -0.02)* | 0.02 (0.00 to 0.03) |
| $p_{\text{non-linearity}}$ | | | **$5*10^{-48}$** | 0.417 | 0.001 | | **$1*10^{-40}$** | **$9*10^{-6}$** | 0.013 |

**ABSI** – a body shape index; **ALP** – alkaline phosphatase; **ALT** – alanine aminotransferase; **ApoA1** – apolipoprotein A1; **ApoB** – apolipoprotein B; **AST** – aspartate aminotransferase; **BMI** – body mass index; **Count** – number of participants with available biomarker measurements; **CRP** – C-reactive protein; **GGT** – gamma-glutamyltransferase; **HbA1c** – haemoglobin A1c (glycated haemoglobin); **HDL-C** – high-density lipoprotein cholesterol; **HI** – hip index; **LDL-C** – low-density lipoprotein cholesterol; **SD** – standard deviation; **SB1, SB2** – first and second spline base.

**LIN** – estimates for SD difference (95% confidence interval) were obtained from multivariable linear regression models with each biomarker on a continuous scale (sex-specific z-scores, following log-transformation) as an outcome variable and BMI+ABSI+HI on a continuous scale (sex-specific z-scores) as independent variables (Figure 1); **SB1+SB2** – estimates for SD difference (95% confidence interval) were obtained from multivariable linear regression models as in LIN, but replacing one of BMI, ABSI, or HI with restricted cubic splines (knots at -2, 0, and 2) and retaining the other two anthropometric indices on a linear scale. All models were additionally adjusted for height, age at enrolment, weight change within the last year preceding enrolment, smoking status, alcohol consumption, physical activity, Townsend deprivation index, region of the assessment centre, time of blood collection, fasting time, use of nonsteroidal anti-inflammatory drugs, paracetamol use, and in women also menopausal status, oral contraceptives use, hormone replacement therapy use, and age at the last live birth. Covariates are defined in Supplementary Methods.

* – $p<0.0001$ from Wald test for the individual term; ** – $p<1*10^{-6}$

**$p_{\text{non-linearity}}$** – p-value from a likelihood ratio test comparing a fully adjusted linear model (LIN) nested within the corresponding fully adjusted model including restricted cubic splines for one of the anthropometric indices (SB1+SB2) ($p_{\text{non-linearity}}$ <0.0001 are shown in bold).

## Supplementary Table S5 Associations of biomarkers with body shape phenotypes (categorical)

| | | Bilirubin direct | | ALP | | GGT | | AST | | ALT | |
|---|---|---|---|---|---|---|---|---|---|---|---|
| | | Count | $SD_{diff}$ (95% CI) | Count | $SD_{diff}$ (95% CI) | Count | $SD_{diff}$ (95% CI) | Count | $SD_{diff}$ (95% CI) | Count | $SD_{diff}$ (95% CI) |
| **MEN** | | | | | | | | | | | |
| ALL | Pear | 31,181 | reference | 31,285 | reference | 31,272 | reference | 31,175 | reference | 31,275 | reference |
| | Slim | 35,575 | -0.05 (-0.06 to -0.03)** | 35,738 | 0.02 ( 0.00 to 0.03) | 35,722 | 0.09 (0.07 to 0.10)** | 35,571 | 0.05 (0.04 to 0.07)** | 35,693 | 0.11 (0.09 to 0.12)** |
| | Wide | 30,911 | -0.09 (-0.11 to -0.08)** | 31,035 | 0.13 (0.12 to 0.15)** | 31,022 | 0.16 (0.14 to 0.17)** | 30,909 | 0.02 (0.01 to 0.04) | 31,017 | 0.13 (0.12 to 0.15)** |
| | Apple | 18,266 | -0.13 (-0.15 to -0.11)** | 18,343 | 0.16 (0.14 to 0.17)** | 18,337 | 0.26 (0.24 to 0.27)** | 18,258 | 0.07 (0.06 to 0.09)** | 18,326 | 0.24 (0.22 to 0.25)** |
| $p_{\text{body shape}}$ | | | **$4*10^{-51}$** | | **$2*10^{-103}$** | | **$1*10^{-200}$** | | **$4*10^{-18}$** | | **$4*10^{-171}$** |
| NW | Pear | 10,654 | reference | 10,677 | reference | 10,674 | reference | 10,651 | reference | 10,681 | reference |
| | Slim | 9,658 | -0.06 (-0.09 to -0.03)* | 9,708 | 0.00 (-0.02 to 0.03) | 9,703 | 0.06 (0.03 to 0.09)* | 9,663 | 0.04 (0.01 to 0.07) | 9,703 | 0.08 (0.05 to 0.10)** |
| | Wide | 9,815 | -0.15 (-0.18 to -0.13)** | 9,850 | 0.16 (0.14 to 0.19)** | 9,848 | 0.18 (0.15 to 0.21)** | 9,820 | 0.01 (-0.02 to 0.03) | 9,852 | 0.14 (0.12 to 0.17)** |
| | Apple | 4,390 | -0.22 (-0.26 to -0.19)** | 4,406 | 0.19 (0.15 to 0.22)** | 4,404 | 0.31 (0.27 to 0.34)** | 4,389 | 0.05 (0.02 to 0.09) | 4,403 | 0.24 (0.20 to 0.27)** |
| OW | Pear | 15,156 | -0.14 (-0.16 to -0.12)** | 15,209 | 0.09 (0.06 to 0.11)** | 15,200 | 0.34 (0.32 to 0.37)** | 15,157 | 0.13 (0.10 to 0.15)** | 15,200 | 0.41 (0.39 to 0.43)** |
| | Slim | 19,107 | -0.20 (-0.22 to -0.18)** | 19,169 | 0.12 (0.10 to 0.15)** | 19,162 | 0.45 (0.43 to 0.47)** | 19,102 | 0.18 (0.16 to 0.21)** | 19,150 | 0.53 (0.51 to 0.56)** |
| | Wide | 15,005 | -0.22 (-0.25 to -0.20)** | 15,062 | 0.22 (0.19 to 0.24)** | 15,050 | 0.52 (0.49 to 0.54)** | 14,996 | 0.16 (0.13 to 0.18)** | 15,050 | 0.57 (0.54 to 0.59)** |
| | Apple | 9,818 | -0.27 (-0.29 to -0.24)** | 9,854 | 0.26 (0.23 to 0.28)** | 9,853 | 0.61 (0.59 to 0.64)** | 9,813 | 0.21 (0.18 to 0.24)** | 9,848 | 0.67 (0.65 to 0.70)** |
| OB | Pear | 5,371 | -0.27 (-0.30 to -0.24)** | 5,399 | 0.21 (0.18 to 0.24)** | 5,398 | 0.76 (0.73 to 0.79)** | 5,367 | 0.40 (0.37 to 0.43)** | 5,394 | 0.92 (0.89 to 0.95)** |
| | Slim | 6,810 | -0.25 (-0.28 to -0.22)** | 6,861 | 0.19 (0.16 to 0.22)** | 6,857 | 0.83 (0.80 to 0.86)** | 6,806 | 0.45 (0.42 to 0.48)** | 6,840 | 1.02 (1.00 to 1.05)** |
| | Wide | 6,091 | -0.28 (-0.31 to -0.24)** | 6,123 | 0.29 (0.26 to 0.32)** | 6,124 | 0.83 (0.80 to 0.86)** | 6,093 | 0.42 (0.39 to 0.46)** | 6,115 | 0.98 (0.95 to 1.01)** |
| | Apple | 4,058 | -0.27 (-0.31 to -0.24)** | 4,083 | 0.29 (0.26 to 0.33)** | 4,080 | 0.91 (0.88 to 0.95)** | 4,056 | 0.48 (0.45 to 0.52)** | 4,075 | 1.10 (1.06 to 1.13)** |
| $p_{\text{interaction}}$ | | | **$8*10^{-17}$** | | **$1*10^{-5}$** | | **$8*10^{-14}$** | | 0.785 | | **$7*10^{-9}$** |
| **WOMEN** | | | | | | | | | | | |
| ALL | Pear | 34,257 | reference | 34,390 | reference | 34,369 | reference | 34,260 | reference | 34,389 | reference |
| | Slim | 29,522 | -0.05 (-0.07 to -0.04)** | 29,635 | -0.04 (-0.05 to -0.02)** | 29,622 | 0.07 (0.05 to 0.08)** | 29,515 | 0.06 (0.04 to 0.07)** | 29,636 | 0.10 (0.08 to 0.11)** |
| | Wide | 37,685 | -0.13 (-0.15 to -0.12)** | 37,794 | 0.11 (0.09 to 0.12)** | 37,778 | 0.16 (0.15 to 0.17)** | 37,680 | 0.05 (0.03 to 0.06)** | 37,797 | 0.16 (0.15 to 0.18)** |
| | Apple | 27,473 | -0.17 (-0.19 to -0.16)** | 27,561 | 0.10 (0.09 to 0.12)** | 27,553 | 0.29 (0.27 to 0.30)** | 27,450 | 0.13 (0.12 to 0.15)** | 27,551 | 0.32 (0.31 to 0.34)** |
| $p_{\text{body shape}}$ | | | **$8*10^{-131}$** | | **$3*10^{-131}$** | | **$<1*10^{-317}$** | | **$5*10^{-63}$** | | **$<1*10^{-317}$** |
| NW | Pear | 17,825 | reference | 17,895 | reference | 17,885 | reference | 17,824 | reference | 17,894 | reference |
| | Slim | 14,592 | -0.04 (-0.06 to -0.02) | 14,648 | -0.03 (-0.05 to -0.02) | 14,638 | 0.03 (0.01 to 0.05) | 14,592 | 0.06 (0.04 to 0.08)** | 14,648 | 0.07 (0.05 to 0.09)** |
| | Wide | 16,268 | -0.15 (-0.17 to -0.13)** | 16,320 | 0.11 (0.09 to 0.13)** | 16,314 | 0.09 (0.07 to 0.11)** | 16,275 | 0.01 (-0.01 to 0.03) | 16,324 | 0.10 (0.08 to 0.12)** |
| | Apple | 10,085 | -0.20 (-0.22 to -0.18)** | 10,119 | 0.13 (0.10 to 0.15)** | 10,114 | 0.17 (0.15 to 0.19)** | 10,080 | 0.05 (0.03 to 0.07)* | 10,115 | 0.20 (0.18 to 0.22)** |
| OW | Pear | 11,222 | -0.16 (-0.18 to -0.14)** | 11,266 | 0.18 (0.16 to 0.20)** | 11,258 | 0.10 (0.08 to 0.12)** | 11,227 | -0.05 (-0.08 to -0.03)* | 11,266 | 0.14 (0.12 to 0.16)** |
| | Slim | 10,706 | -0.23 (-0.25 to -0.21)** | 10,747 | 0.17 (0.14 to 0.19)** | 10,746 | 0.20 (0.17 to 0.22)** | 10,701 | 0.00 (-0.03 to 0.02) | 10,749 | 0.25 (0.23 to 0.27)** |
| | Wide | 13,328 | -0.30 (-0.32 to -0.28)** | 13,372 | 0.32 (0.30 to 0.34)** | 13,364 | 0.33 (0.31 to 0.35)** | 13,326 | 0.02 (0.00 to 0.04) | 13,369 | 0.36 (0.34 to 0.38)** |
| | Apple | 11,891 | -0.33 (-0.36 to -0.31)** | 11,920 | 0.31 (0.29 to 0.33)** | 11,919 | 0.47 (0.45 to 0.49)** | 11,879 | 0.10 (0.08 to 0.13)** | 11,918 | 0.54 (0.52 to 0.56)** |
| OB | Pear | 5,210 | -0.28 (-0.31 to -0.25)** | 5,229 | 0.51 (0.48 to 0.54)** | 5,226 | 0.47 (0.44 to 0.50)** | 5,209 | 0.04 (0.01 to 0.07) | 5,229 | 0.49 (0.46 to 0.52)** |
| | Slim | 4,224 | -0.35 (-0.39 to -0.32)** | 4,240 | 0.43 (0.40 to 0.46)** | 4,238 | 0.61 (0.58 to 0.64)** | 4,222 | 0.12 (0.09 to 0.15)** | 4,239 | 0.67 (0.63 to 0.70)** |
| | Wide | 8,089 | -0.36 (-0.39 to -0.34)** | 8,102 | 0.53 (0.51 to 0.55)** | 8,100 | 0.70 (0.67 to 0.72)** | 8,079 | 0.16 (0.13 to 0.19)** | 8,104 | 0.73 (0.70 to 0.75)** |
| | Apple | 5,497 | -0.39 (-0.42 to -0.36)** | 5,522 | 0.49 (0.47 to 0.52)** | 5,520 | 0.86 (0.83 to 0.88)** | 5,491 | 0.32 (0.29 to 0.35)** | 5,518 | 0.93 (0.90 to 0.96)** |
| $p_{\text{interaction}}$ | | | **$2*10^{-8}$** | | **$3*10^{-13}$** | | **$5*10^{-41}$** | | **$4*10^{-29}$** | | **$2*10^{-43}$** |

| | | HDL-C | | ApoA1 | | LDL-C | | ApoB | | Triglycerides | |
|---|---|---|---|---|---|---|---|---|---|---|---|
| | | Count | $SD_{diff}$ (95% CI) | Count | $SD_{diff}$ (95% CI) | Count | $SD_{diff}$ (95% CI) | Count | $SD_{diff}$ (95% CI) | Count | $SD_{diff}$ (95% CI) |
| **MEN** | | | | | | | | | | | |
| ALL | Pear | 28,891 | reference | 28,870 | reference | 31,236 | reference | 31,177 | reference | 31,264 | reference |
| | Slim | 32,954 | -0.11 (-0.13 to -0.10)** | 32,945 | -0.08 (-0.09 to -0.06)** | 35,645 | 0.07 (0.05 to 0.08)** | 35,536 | 0.09 (0.08 to 0.11)** | 35,710 | 0.16 (0.14 to 0.17)** |
| | Wide | 28,627 | -0.11 (-0.13 to -0.10)** | 28,615 | -0.07 (-0.09 to -0.06)** | 30,967 | 0.09 (0.08 to 0.11)** | 30,900 | 0.11 (0.09 to 0.12)** | 31,018 | 0.17 (0.16 to 0.19)** |
| | Apple | 16,952 | -0.21 (-0.23 to -0.19)** | 16,947 | -0.14 (-0.15 to -0.12)** | 18,295 | 0.12 (0.10 to 0.14)** | 18,227 | 0.16 (0.14 to 0.18)** | 18,332 | 0.32 (0.30 to 0.34)** |
| $p_{body\ shape}$ | | | **$8*10^{-131}$** | | **$3*10^{-53}$** | | **$6*10^{-47}$** | | **$4*10^{-77}$** | | **$1*10^{-303}$** |
| NW | Pear | 9,809 | reference | 9,799 | reference | 10,663 | reference | 10,665 | reference | 10,672 | reference |
| | Slim | 8,933 | -0.09 (-0.12 to -0.07)** | 8,924 | -0.06 (-0.09 to -0.04)* | 9,685 | 0.08 (0.05 to 0.11)** | 9,689 | 0.10 (0.07 to 0.12)** | 9,701 | 0.12 (0.09 to 0.14)** |
| | Wide | 9,012 | -0.17 (-0.19 to -0.14)** | 9,000 | -0.12 (-0.14 to -0.09)** | 9,830 | 0.18 (0.15 to 0.21)** | 9,835 | 0.19 (0.16 to 0.21)** | 9,844 | 0.26 (0.24 to 0.29)** |
| | Apple | 4,061 | -0.29 (-0.32 to -0.25)** | 4,061 | -0.21 (-0.24 to -0.17)** | 4,398 | 0.24 (0.21 to 0.28)** | 4,396 | 0.28 (0.25 to 0.31)** | 4,403 | 0.43 (0.39 to 0.46)** |
| OW | Pear | 14,066 | -0.46 (-0.49 to -0.44)** | 14,055 | -0.37 (-0.39 to -0.34)** | 15,184 | 0.34 (0.32 to 0.36)** | 15,157 | 0.38 (0.35 to 0.40)** | 15,194 | 0.52 (0.49 to 0.54)** |
| | Slim | 17,647 | -0.58 (-0.61 to -0.56)** | 17,643 | -0.44 (-0.47 to -0.42)** | 19,125 | 0.40 (0.38 to 0.43)** | 19,070 | 0.47 (0.45 to 0.49)** | 19,154 | 0.69 (0.67 to 0.71)** |
| | Wide | 13,880 | -0.57 (-0.60 to -0.55)** | 13,879 | -0.44 (-0.46 to -0.41)** | 15,026 | 0.43 (0.41 to 0.46)** | 14,990 | 0.48 (0.46 to 0.51)** | 15,055 | 0.69 (0.67 to 0.72)** |
| | Apple | 9,097 | -0.67 (-0.70 to -0.64)** | 9,085 | -0.49 (-0.52 to -0.46)** | 9,833 | 0.45 (0.42 to 0.47)** | 9,791 | 0.53 (0.50 to 0.56)** | 9,850 | 0.84 (0.81 to 0.87)** |
| OB | Pear | 5,016 | -0.85 (-0.88 to -0.82)** | 5,016 | -0.65 (-0.68 to -0.62)** | 5,389 | 0.45 (0.42 to 0.48)** | 5,355 | 0.56 (0.53 to 0.60)** | 5,398 | 0.94 (0.91 to 0.97)** |
| | Slim | 6,374 | -0.97 (-1.00 to -0.94)** | 6,378 | -0.74 (-0.76 to -0.71)** | 6,835 | 0.47 (0.44 to 0.50)** | 6,777 | 0.61 (0.58 to 0.64)** | 6,855 | 1.08 (1.05 to 1.11)** |
| | Wide | 5,735 | -0.88 (-0.91 to -0.85)** | 5,736 | -0.65 (-0.68 to -0.62)** | 6,111 | 0.40 (0.37 to 0.43)** | 6,075 | 0.52 (0.49 to 0.56)** | 6,119 | 0.94 (0.91 to 0.97)** |
| | Apple | 3,794 | -0.98 (-1.01 to -0.94)** | 3,801 | -0.72 (-0.76 to -0.69)** | 4,064 | 0.44 (0.41 to 0.48)** | 4,040 | 0.59 (0.55 to 0.62)** | 4,079 | 1.10 (1.07 to 1.13)** |
| $p_{interaction}$ | | | **$1*10^{-19}$** | | **$2*10^{-13}$** | | **$7*10^{-32}$** | | **$2*10^{-32}$** | | **$2*10^{-62}$** |
| **WOMEN** | | | | | | | | | | | |
| ALL | Pear | 31,245 | reference | 31,166 | reference | 34,340 | reference | 34,372 | reference | 34,364 | reference |
| | Slim | 26,893 | -0.15 (-0.16 to -0.13)** | 26,830 | -0.10 (-0.11 to -0.08)** | 29,581 | 0.09 (0.08 to 0.11)** | 29,617 | 0.11 (0.09 to 0.12)** | 29,621 | 0.17 (0.16 to 0.19)** |
| | Wide | 34,269 | -0.23 (-0.24 to -0.22)** | 34,187 | -0.14 (-0.15 to -0.12)** | 37,720 | 0.17 (0.15 to 0.18)** | 37,755 | 0.19 (0.17 to 0.20)** | 37,763 | 0.33 (0.31 to 0.34)** |
| | Apple | 25,030 | -0.44 (-0.45 to -0.42)** | 24,985 | -0.28 (-0.29 to -0.26)** | 27,517 | 0.30 (0.28 to 0.31)** | 27,506 | 0.34 (0.33 to 0.36)** | 27,546 | 0.58 (0.56 to 0.59)** |
| $p_{body\ shape}$ | | | **$<1*10^{-317}$** | | **$2*10^{-278}$** | | **$<1*10^{-317}$** | | **$<1*10^{-317}$** | | **$<1*10^{-317}$** |
| NW | Pear | 16,273 | reference | 16,215 | reference | 17,867 | reference | 17,888 | reference | 17,874 | reference |
| | Slim | 13,259 | -0.12 (-0.14 to -0.09)** | 13,214 | -0.08 (-0.10 to -0.06)** | 14,615 | 0.06 (0.04 to 0.08)** | 14,644 | 0.07 (0.05 to 0.09)** | 14,640 | 0.11 (0.09 to 0.13)** |
| | Wide | 14,763 | -0.22 (-0.24 to -0.20)** | 14,711 | -0.14 (-0.16 to -0.12)** | 16,289 | 0.16 (0.14 to 0.18)** | 16,310 | 0.18 (0.16 to 0.20)** | 16,312 | 0.30 (0.28 to 0.32)** |
| | Apple | 9,166 | -0.39 (-0.41 to -0.37)** | 9,142 | -0.25 (-0.27 to -0.22)** | 10,097 | 0.29 (0.27 to 0.31)** | 10,104 | 0.32 (0.30 to 0.34)** | 10,116 | 0.51 (0.48 to 0.53)** |
| OW | Pear | 10,200 | -0.31 (-0.33 to -0.29)** | 10,183 | -0.26 (-0.29 to -0.24)** | 11,249 | 0.20 (0.18 to 0.23)** | 11,258 | 0.22 (0.19 to 0.24)** | 11,262 | 0.28 (0.26 to 0.30)** |
| | Slim | 9,763 | -0.50 (-0.52 to -0.48)** | 9,749 | -0.38 (-0.41 to -0.36)** | 10,737 | 0.33 (0.31 to 0.35)** | 10,741 | 0.36 (0.34 to 0.38)** | 10,743 | 0.52 (0.50 to 0.54)** |
| | Wide | 12,157 | -0.56 (-0.59 to -0.54)** | 12,135 | -0.41 (-0.43 to -0.38)** | 13,342 | 0.41 (0.39 to 0.43)** | 13,355 | 0.44 (0.42 to 0.46)** | 13,353 | 0.67 (0.65 to 0.69)** |
| | Apple | 10,824 | -0.82 (-0.84 to -0.80)** | 10,804 | -0.59 (-0.61 to -0.56)** | 11,910 | 0.53 (0.51 to 0.55)** | 11,891 | 0.60 (0.58 to 0.62)** | 11,913 | 0.97 (0.95 to 0.99)** |
| OB | Pear | 4,772 | -0.72 (-0.75 to -0.69)** | 4,768 | -0.55 (-0.58 to -0.52)** | 5,224 | 0.34 (0.31 to 0.37)** | 5,226 | 0.39 (0.36 to 0.42)** | 5,228 | 0.70 (0.67 to 0.72)** |
| | Slim | 3,871 | -0.89 (-0.92 to -0.86)** | 3,867 | -0.65 (-0.69 to -0.62)** | 4,229 | 0.48 (0.45 to 0.51)** | 4,232 | 0.55 (0.52 to 0.58)** | 4,238 | 0.95 (0.92 to 0.98)** |
| | Wide | 7,349 | -0.94 (-0.97 to -0.92)** | 7,341 | -0.67 (-0.70 to -0.65)** | 8,089 | 0.46 (0.44 to 0.49)** | 8,090 | 0.55 (0.53 to 0.57)** | 8,098 | 1.01 (0.98 to 1.03)** |
| | Apple | 5,040 | -1.11 (-1.14 to -1.08)** | 5,039 | -0.79 (-0.82 to -0.76)** | 5,510 | 0.61 (0.58 to 0.63)** | 5,511 | 0.71 (0.69 to 0.74)** | 5,517 | 1.23 (1.20 to 1.26)** |
| $p_{interaction}$ | | | **$2*10^{-14}$** | | **$3*10^{-6}$** | | **$4*10^{-11}$** | | **$5*10^{-10}$** | | **$2*10^{-40}$** |

| | | HbA1c | | Glucose | | Lymphocytes | | Neutrophils | | CRP | |
|---|---|---|---|---|---|---|---|---|---|---|---|
| | | Count | $SD_{diff}$ (95% CI) | Count | $SD_{diff}$ (95% CI) | Count | $SD_{diff}$ (95% CI) | Count | $SD_{diff}$ (95% CI) | Count | $SD_{diff}$ (95% CI) |
| **MEN** | | | | | | | | | | | |
| ALL | Pear | 31,151 | reference | 28,864 | reference | 31,875 | reference | 31,875 | reference | 31,250 | reference |
| | Slim | 35,677 | 0.05 (0.03 to 0.06)** | 32,931 | 0.07 (0.05 to 0.08)** | 36,365 | 0.02 (0.01 to 0.04) | 36,365 | 0.02 (0.01 to 0.04) | 35,653 | 0.04 (0.03 to 0.06)** |
| | Wide | 30,914 | 0.06 (0.05 to 0.08)** | 28,608 | 0.04 (0.02 to 0.06)* | 31,530 | 0.05 (0.03 to 0.06)** | 31,530 | 0.12 (0.11 to 0.14)** | 30,987 | 0.20 (0.18 to 0.21)** |
| | Apple | 18,285 | 0.13 (0.11 to 0.15)** | 16,943 | 0.11 (0.09 to 0.13)** | 18,695 | 0.08 (0.06 to 0.09)** | 18,695 | 0.16 (0.14 to 0.18)** | 18,312 | 0.25 (0.23 to 0.27)** |
| p body shape | | | **$4*10^{-46}$** | | **$5*10^{-31}$** | | **$2*10^{-18}$** | | **$2*10^{-103}$** | | **$6*10^{-264}$** |
| NW | Pear | 10,641 | reference | 9,800 | reference | 10,866 | reference | 10,866 | reference | 10,671 | reference |
| | Slim | 9,708 | 0.04 (0.01 to 0.07) | 8,923 | 0.04 (0.02 to 0.07) | 9,900 | 0.02 (-0.01 to 0.05) | 9,900 | 0.03 (0.01 to 0.06) | 9,694 | 0.08 (0.06 to 0.11)** |
| | Wide | 9,790 | 0.01 (-0.01 to 0.04) | 9,004 | 0.02 (-0.01 to 0.05) | 10,013 | 0.05 (0.02 to 0.07) | 10,013 | 0.13 (0.11 to 0.16)** | 9,836 | 0.25 (0.22 to 0.28)** |
| | Apple | 4,428 | 0.08 (0.05 to 0.11)* | 4,058 | 0.10 (0.06 to 0.13)** | 4,508 | 0.09 (0.06 to 0.12)** | 4,508 | 0.19 (0.16 to 0.23)** | 4,400 | 0.35 (0.32 to 0.39)** |
| OW | Pear | 15,141 | 0.07 (0.05 to 0.09)** | 14,050 | 0.10 (0.08 to 0.13)** | 15,526 | 0.21 (0.19 to 0.23)** | 15,526 | 0.10 (0.08 to 0.12)** | 15,188 | 0.47 (0.45 to 0.49)** |
| | Slim | 19,135 | 0.12 (0.09 to 0.14)** | 17,640 | 0.17 (0.15 to 0.20)** | 19,512 | 0.24 (0.22 to 0.26)** | 19,512 | 0.12 (0.10 to 0.14)** | 19,126 | 0.51 (0.48 to 0.53)** |
| | Wide | 15,024 | 0.14 (0.11 to 0.16)** | 13,870 | 0.13 (0.11 to 0.16)** | 15,286 | 0.26 (0.24 to 0.28)** | 15,286 | 0.21 (0.19 to 0.24)** | 15,043 | 0.65 (0.63 to 0.67)** |
| | Apple | 9,804 | 0.20 (0.18 to 0.23)** | 9,087 | 0.19 (0.17 to 0.22)** | 10,032 | 0.29 (0.27 to 0.32)** | 10,032 | 0.25 (0.22 to 0.28)** | 9,841 | 0.71 (0.69 to 0.74)** |
| OB | Pear | 5,369 | 0.32 (0.29 to 0.35)** | 5,014 | 0.25 (0.21 to 0.28)** | 5,483 | 0.45 (0.42 to 0.48)** | 5,483 | 0.24 (0.21 to 0.27)** | 5,391 | 0.95 (0.92 to 0.98)** |
| | Slim | 6,834 | 0.38 (0.35 to 0.41)** | 6,368 | 0.34 (0.31 to 0.37)** | 6,953 | 0.46 (0.43 to 0.49)** | 6,953 | 0.25 (0.22 to 0.28)** | 6,833 | 0.94 (0.91 to 0.96)** |
| | Wide | 6,100 | 0.45 (0.42 to 0.48)** | 5,734 | 0.35 (0.31 to 0.38)** | 6,231 | 0.50 (0.47 to 0.53)** | 6,231 | 0.36 (0.33 to 0.39)** | 6,108 | 1.09 (1.06 to 1.11)** |
| | Apple | 4,053 | 0.52 (0.49 to 0.56)** | 3,798 | 0.43 (0.40 to 0.47)** | 4,155 | 0.50 (0.46 to 0.53)** | 4,155 | 0.37 (0.34 to 0.41)** | 4,071 | 1.07 (1.04 to 1.11)** |
| p interaction | | | **$7*10^{-8}$** | | 0.002 | | 0.499 | | 0.337 | | **$2*10^{-17}$** |
| **WOMEN** | | | | | | | | | | | |
| ALL | Pear | 34,173 | reference | 31,227 | reference | 34,850 | reference | 34,850 | reference | 34,355 | reference |
| | Slim | 29,549 | 0.06 (0.05 to 0.08)** | 26,871 | 0.05 (0.04 to 0.07)** | 30,083 | 0.04 (0.03 to 0.06)** | 30,083 | -0.01 (-0.02 to 0.01) | 29,612 | -0.01 (-0.02 to 0.01) |
| | Wide | 37,607 | 0.10 (0.09 to 0.11)** | 34,244 | 0.05 (0.03 to 0.06)** | 38,267 | 0.07 (0.05 to 0.08)** | 38,267 | 0.10 (0.08 to 0.11)** | 37,761 | 0.17 (0.15 to 0.18)** |
| | Apple | 27,463 | 0.19 (0.18 to 0.21)** | 25,016 | 0.13 (0.11 to 0.15)** | 27,949 | 0.14 (0.13 to 0.16)** | 27,949 | 0.10 (0.08 to 0.11)** | 27,521 | 0.20 (0.19 to 0.22)** |
| p body shape | | | **$2*10^{-147}$** | | **$4*10^{-53}$** | | **$7*10^{-79}$** | | **$3*10^{-77}$** | | **$1*10^{-313}$** |
| NW | Pear | 17,695 | reference | 16,262 | reference | 18,091 | reference | 18,091 | reference | 17,873 | reference |
| | Slim | 14,576 | 0.04 (0.02 to 0.06) | 13,253 | 0.04 (0.01 to 0.06) | 14,897 | 0.02 (0.00 to 0.04) | 14,897 | -0.02 (-0.04 to 0.00) | 14,638 | -0.01 (-0.03 to 0.01) |
| | Wide | 16,276 | 0.05 (0.03 to 0.07)* | 14,751 | 0.04 (0.01 to 0.06) | 16,574 | 0.04 (0.02 to 0.06)* | 16,574 | 0.10 (0.08 to 0.12)** | 16,306 | 0.15 (0.13 to 0.17)** |
| | Apple | 10,088 | 0.12 (0.10 to 0.14)** | 9,163 | 0.11 (0.08 to 0.13)** | 10,277 | 0.10 (0.08 to 0.12)** | 10,277 | 0.08 (0.06 to 0.11)** | 10,105 | 0.20 (0.18 to 0.22)** |
| OW | Pear | 11,271 | 0.03 (0.01 to 0.05) | 10,194 | 0.07 (0.05 to 0.10)** | 11,457 | 0.11 (0.08 to 0.13)** | 11,457 | 0.08 (0.06 to 0.11)** | 11,257 | 0.47 (0.45 to 0.49)** |
| | Slim | 10,729 | 0.10 (0.08 to 0.12)** | 9,748 | 0.15 (0.12 to 0.17)** | 10,895 | 0.17 (0.15 to 0.19)** | 10,895 | 0.09 (0.07 to 0.11)** | 10,742 | 0.48 (0.46 to 0.50)** |
| | Wide | 13,273 | 0.15 (0.13 to 0.17)** | 12,152 | 0.13 (0.11 to 0.15)** | 13,536 | 0.21 (0.19 to 0.23)** | 13,536 | 0.19 (0.17 to 0.21)** | 13,361 | 0.67 (0.65 to 0.69)** |
| | Apple | 11,875 | 0.25 (0.23 to 0.28)** | 10,816 | 0.23 (0.21 to 0.26)** | 12,085 | 0.30 (0.28 to 0.32)** | 12,085 | 0.21 (0.19 to 0.23)** | 11,904 | 0.72 (0.70 to 0.74)** |
| OB | Pear | 5,207 | 0.22 (0.20 to 0.25)** | 4,771 | 0.25 (0.22 to 0.28)** | 5,302 | 0.30 (0.27 to 0.33)** | 5,302 | 0.31 (0.28 to 0.34)** | 5,225 | 1.18 (1.16 to 1.21)** |
| | Slim | 4,244 | 0.33 (0.30 to 0.36)** | 3,870 | 0.31 (0.27 to 0.34)** | 4,291 | 0.37 (0.34 to 0.40)** | 4,291 | 0.31 (0.28 to 0.34)** | 4,232 | 1.16 (1.13 to 1.18)** |
| | Wide | 8,058 | 0.43 (0.41 to 0.46)** | 7,341 | 0.32 (0.30 to 0.35)** | 8,157 | 0.37 (0.34 to 0.39)** | 8,157 | 0.40 (0.37 to 0.42)** | 8,094 | 1.32 (1.30 to 1.34)** |
| | Apple | 5,500 | 0.54 (0.51 to 0.56)** | 5,037 | 0.38 (0.35 to 0.41)** | 5,587 | 0.44 (0.41 to 0.47)** | 5,587 | 0.38 (0.35 to 0.41)** | 5,512 | 1.29 (1.27 to 1.32)** |
| p interaction | | | **$6*10^{-24}$** | | 0.035 | | **$2*10^{-6}$** | | 0.025 | | **$5*10^{-11}$** |

**ABSI** – a body shape index (cut-offs ≥80 for men, ≥73 for women); **ALP** – alkaline phosphatase; **ALT** – alanine aminotransferase; **ApoA1** – apolipoprotein A1; **ApoB** – apolipoprotein B; **Apple** – large-ABSI-small-HI; **AST** – aspartate aminotransferase; **BMI** – body mass index; **Count** – number of participants with available biomarker measurements per category; **CRP** – C-reactive protein; **GGT** – gamma-glutamyltransferase; **HbA1c** – haemoglobin A1c (glycated haemoglobin); **HDL-C** – high-density lipoprotein cholesterol; **HI** – hip index (cut-offs ≥49 for men, ≥64 for women); **LDL-C** – low-density lipoprotein cholesterol; **NW** – normal weight (BMI≥18.5 to <25 kg/m$^2$); **OB** – obese (BMI≥30 to <45 kg/m$^2$); **OW** – overweight (BMI≥25 to <30 kg/m$^2$); **Pear** – small-ABSI-large-HI; **Slim** – small-ABSI-small-HI; **Wide** – large-ABSI-large-HI.

**$SD_{diff}$ (95% CI)** – estimates for standard deviation difference (95% confidence interval) were obtained from multivariable linear regression models including each biomarker on a continuous scale (sex-specific z-scores, following log-transformation) as an outcome variable and the following two combinations as independent variables: an ABSI-by-HI cross-classification and BMI categories (for ALL, reference “pear”), or an BMI-by-ABSI-by-HI cross-classification (for NW, OW, and OB, reference “pear”-NW). All models were adjusted for height, age at enrolment, weight change within the last year preceding enrolment, smoking status, alcohol consumption, physical activity, Townsend deprivation index, region of the assessment centre, time of blood collection, fasting time, use of nonsteroidal anti-inflammatory drugs, paracetamol use, and in women also menopausal status, oral contraceptives use, hormone replacement therapy use, and age at the last live birth. Covariates are defined in Supplementary Methods.

**$p_{\text{body shape}}$** (for ALL) – was obtained from a likelihood ratio test comparing a model including only BMI categories and covariates with a model additionally including an ABSI-by-HI cross-classification (evaluates the significance of body-shape overall) ($p_{\text{body shape}}$ <0.0001 are shown in bold).

**$p_{\text{interaction}}$** (for NW, OW, and OB) – was obtained from a likelihood ratio test comparing the additive model including the ABSI-by-HI cross-classification, BMI categories, and covariates with the interaction model including the BMI-by-ABSI-by-HI cross-classification and covariates (evaluates heterogeneity by BMI) ($p_{\text{interaction}}$ <0.0001 are shown in bold).

* – p<0.0001 from Wald test for the individual term; ** – $p<1*10^{-6}$

**Supplementary Table S6 Biomarker boundaries**

| | Unit | LL count | LL cut-off | 2.5th centile | Median | 97.5th centile | UL cut-off | UL count |
|---|---|---|---|---|---|---|---|---|
| **MEN** | | | | | | | | |
| | | | | | | | | |
| Bilirubin total | μmol/L | 12 | 2 | 5.03 | 9.15 | 24.0 | 50 | 58 |
| ALP | U/L | 12 | 15 | 48.1 | 77.8 | 127.7 | 300 | 45 |
| GGT | U/L | 3 | 5 | 14.2 | 31.0 | 135.1 | 1000 | 24 |
| AST | U/L | 7 | 8 | 17.3 | 25.8 | 48.2 | 250 | 23 |
| ALT | U/L | 7 | 3 | 11.4 | 23.2 | 62.9 | 250 | 19 |
| HDL-C | mmol/L | 4 | 0.4 | 0.83 | 1.27 | 2.02 | 3.5 | 10 |
| ApoA1 | g/L | 17 | 0.7 | 1.07 | 1.42 | 1.96 | 2.6 | 0 |
| LDL-C | mmol/L | 121 | 1.5 | 2.34 | 3.71 | 5.37 | 7.5 | 12 |
| ApoB | g/L | 152 | 0.4 | 0.67 | 1.07 | 1.57 | 2.1 | 0 |
| Triglycerides | mmol/L | 16 | 0.3 | 0.64 | 1.66 | 4.96 | 11 | 56 |
| HbA1c | mmol/mol | 56 | 15 | 27.3 | 34.4 | 42.2 | 100 | 42 |
| Glucose | mmol/L | 3 | 2 | 3.78 | 4.89 | 6.46 | 25 | 9 |
| Lymphocytes | $*10^9$/L | 14 | 0.2 | 0.97 | 1.80 | 3.15 | 10 | 28 |
| Neutrophils | $*10^9$/L | 44 | 0.3 | 2.1 | 3.90 | 7.29 | 15 | 9 |
| CRP | mg/L | 25 | 0.08 | 0.2 | 1.15 | 10.9 | 80 | 0 |
| **WOMEN** | | | | | | | | |
| Bilirubin total | μmol/L | 4 | 2 | 4.17 | 7.34 | 18.6 | 50 | 9 |
| ALP | U/L | 13 | 15 | 45.1 | 80.2 | 137.3 | 300 | 49 |
| GGT | U/L | 6 | 5 | 10.4 | 19.7 | 92.3 | 1000 | 7 |
| AST | U/L | 14 | 8 | 15.2 | 22.5 | 39.6 | 250 | 16 |
| ALT | U/L | 18 | 3 | 8.72 | 16.8 | 44.7 | 250 | 24 |
| HDL-C | mmol/L | 2 | 0.4 | 1.02 | 1.60 | 2.46 | 3.5 | 19 |
| ApoA1 | g/L | 5 | 0.7 | 1.20 | 1.63 | 2.26 | 2.6 | 0 |
| LDL-C | mmol/L | 95 | 1.5 | 2.28 | 3.68 | 5.55 | 7.5 | 21 |
| ApoB | g/L | 196 | 0.4 | 0.64 | 1.04 | 1.58 | 2.1 | 0 |
| Triglycerides | mmol/L | 7 | 0.3 | 0.57 | 1.25 | 3.52 | 11 | 6 |
| HbA1c | mmol/mol | 47 | 15 | 27.6 | 34.6 | 41.8 | 100 | 25 |
| Glucose | mmol/L | 2 | 2 | 3.88 | 4.88 | 6.45 | 25 | 0 |
| Lymphocytes | $*10^9$/L | 18 | 0.2 | 1.04 | 1.90 | 3.30 | 10 | 21 |
| Neutrophils | $*10^9$/L | 51 | 0.3 | 2.09 | 3.90 | 7.14 | 15 | 6 |
| CRP | mg/L | 48 | 0.08 | 0.19 | 1.13 | 10.9 | 80 | 0 |

**ALP** – alkaline phosphatase; **ALT** – alanine aminotransferase; **ApoA1** – apolipoprotein A1; **ApoB** – apolipoprotein B; **AST** – aspartate aminotransferase; **CRP** – C-reactive protein; **GGT** – gamma-glutamyltransferase; **HbA1c** – haemoglobin A1c (glycated haemoglobin); **HDL-C** – high-density lipoprotein cholesterol; **LDL-C** – low-density lipoprotein cholesterol.

Values shown were used for exclusion of participants with extreme biomarker measurements in Figure 4 as follows:

**LL $_{\text{cut-off}}$** – lower limit cut-off, used for exclusion of extremely low values; **LL $_{\text{count}}$** – number of excluded participants with extremely low values; **UL $_{\text{cut-off}}$** – upper limit cut-off, used for exclusion of extremely high values; **UL $_{\text{count}}$** – number of excluded participants with extremely high values.

**2.5$^{th}$ centile** & **97.5$^{th}$ centile** are marked in Figure 4.

**Supplementary Table S7 Associations of biomarkers with body size and body shape indices: subgroups according to biomarker levels**

| | | | | BMI | | ABSI | | HI | |
|---|---|---|---|---|---|---|---|---|---|
| | Cut-off | | Count | $SD_{diff}$ (95% CI) | p-value | $SD_{diff}$ (95% CI) | p-value | $SD_{diff}$ (95% CI) | p-value |
| **MEN** | | | | | | | | | |
| **AST** | 70 | Below | 115,201 | 0.146 (0.141 to 0.152)** | $<1*10^{-317}$ | 0.008 (0.002 to 0.013) | 0.009 | -0.032 (-0.038 to -0.027)** | $5*10^{-31}$ |
| | | Above | 712 | **-0.180 (-0.274 to -0.086)** | **$2*10^{-4}$** | 0.021 (-0.087 to 0.129) | 0.707 | -0.002 (-0.105 to 0.100) | 0.963 |
| **ALT** | 10 | Below | 1421 | -0.021 (-0.053 to 0.012) | 0.207 | 0.001 (-0.028 to 0.029) | 0.966 | 0.010 (-0.017 to 0.038) | 0.464 |
| | | Above | 114,890 | 0.322 (0.317 to 0.327)** | $<1*10^{-317}$ | 0.095 (0.090 to 0.101)** | $3*10^{-248}$ | -0.080 (-0.085 to -0.075)** | $5*10^{-193}$ |
| **ALT** | 70 | Below | 114,354 | 0.307 (0.302 to 0.312)** | $<1*10^{-317}$ | 0.088 (0.082 to 0.093)** | $1*10^{-226}$ | -0.074 (-0.079 to -0.069)** | $7*10^{-180}$ |
| | | Above | 1957 | **-0.057 (-0.083 to -0.031)*** | **$2*10^{-5}$** | -0.005 (-0.036 to 0.025) | 0.738 | -0.002 (-0.030 to 0.026) | 0.900 |
| **HDL-C** | 1.8 | Below | 100,063 | -0.237 (-0.242 to -0.232)** | $<1*10^{-317}$ | -0.066 (-0.072 to -0.061)** | $3*10^{-127}$ | 0.067 (0.062 to 0.072)** | $6*10^{-145}$ |
| | | Above | 7361 | -0.080 (-0.093 to -0.066)** | $2*10^{-30}$ | **0.021 (0.010 to 0.031)*** | **$9*10^{-5}$** | -0.003 (-0.013 to 0.008) | 0.629 |
| **ApoA1** | 1.8 | Below | 100,397 | -0.177 (-0.182 to -0.172)** | $<1*10^{-317}$ | -0.049 (-0.055 to -0.044)** | $3*10^{-70}$ | 0.048 (0.042 to 0.053)** | $2*10^{-72}$ |
| | | Above | 6980 | -0.061 (-0.075 to -0.046)** | $1*10^{-16}$ | **0.025 (0.014 to 0.037)*** | **$2*10^{-5}$** | -0.005 (-0.016 to 0.007) | 0.421 |
| **HbA1c** | 30 | Below | 11,114 | 0.006 (-0.015 to 0.026) | 0.591 | **-0.039 (-0.059 to -0.019)** | **$1*10^{-4}$** | 0.012 (-0.007 to 0.031) | 0.222 |
| | | Above | 104,913 | 0.133 (0.128 to 0.137)** | $<1*10^{-317}$ | 0.050 (0.045 to 0.055)** | $7*10^{-80}$ | -0.032 (-0.037 to -0.027)** | $4*10^{-36}$ |
| **HbA1c** | 48 | Below | 115,188 | 0.114 (0.109 to 0.120)** | $<1*10^{-317}$ | 0.032 (0.026 to 0.037)** | $2*10^{-29}$ | -0.024 (-0.029 to -0.018)** | $2*10^{-18}$ |
| | | Above | 839 | -0.153 (-0.267 to -0.038) | 0.009 | 0.202 (0.057 to 0.348) | 0.006 | -0.099 (-0.218 to 0.019) | 0.101 |
| **Neutrophils** | 2.0 | Below | 2125 | -0.070 (-0.144 to 0.003) | 0.061 | -0.110 (-0.176 to -0.043) | 0.001 | 0.034 (-0.030 to 0.098) | 0.294 |
| | | Above | 116,340 | 0.074 (0.069 to 0.079)** | $1*10^{-173}$ | 0.084 (0.079 to 0.089)** | $8*10^{-203}$ | -0.027 (-0.032 to -0.022)** | $2*10^{-24}$ |
| **Neutrophils** | 7.5 | Below | 115,981 | 0.089 (0.084 to 0.095)** | $1*10^{-226}$ | 0.081 (0.076 to 0.087)** | $3*10^{-172}$ | -0.026 (-0.031 to -0.021)** | $8*10^{-21}$ |
| | | Above | 2484 | -0.012 (-0.027 to 0.002) | 0.095 | 0.005 (-0.011 to 0.021) | 0.558 | 0.006 (-0.010 to 0.022) | 0.455 |
| **CRP** | 8.0 | Below | 111,698 | 0.322 (0.317 to 0.327)** | $<1*10^{-317}$ | 0.124 (0.119 to 0.129)** | $<1*10^{-317}$ | -0.042 (-0.047 to -0.037)** | $1*10^{-65}$ |
| | | Above | 4504 | **-0.051 (-0.064 to -0.038)**** | **$1*10^{-14}$** | -0.023 (-0.039 to -0.007) | 0.005 | -0.008 (-0.023 to 0.007) | 0.280 |
| **WOMEN** | | | | | | | | | |
| **ALP** | 35 | Below | 476 | -0.040 (-0.172 to 0.091) | 0.545 | 0.008 (-0.104 to 0.120) | 0.891 | 0.010 (-0.103 to 0.124) | 0.859 |
| | | Above | 128,904 | 0.169 (0.164 to 0.174)** | $<1*10^{-317}$ | 0.075 (0.070 to 0.080)** | $6*10^{-203}$ | 0.008 (0.004 to 0.013) | $6*10^{-4}$ |
| **AST** | 70 | Below | 128,476 | 0.032 (0.027 to 0.038)** | $3*10^{-33}$ | 0.037 (0.032 to 0.042)** | $1*10^{-45}$ | -0.036 (-0.041 to -0.031)** | $8*10^{-45}$ |
| | | Above | 429 | -0.095 (-0.239 to 0.049) | 0.195 | 0.073 (-0.084 to 0.229) | 0.361 | 0.053 (-0.095 to 0.202) | 0.482 |
| **ALT** | 10 | Below | 7314 | -0.009 (-0.022 to 0.005) | 0.206 | 0.002 (-0.009 to 0.014) | 0.690 | 0.009 (-0.003 to 0.020) | 0.146 |
| | | Above | 122,059 | 0.209 (0.204 to 0.214)** | $<1*10^{-317}$ | 0.109 (0.104 to 0.115)** | $<1*10^{-317}$ | -0.077 (-0.082 to -0.072)** | $5*10^{-204}$ |
| **ALT** | 70 | Below | 128,587 | 0.219 (0.214 to 0.224)** | $<1*10^{-317}$ | 0.112 (0.107 to 0.117)** | $<1*10^{-317}$ | -0.077 (-0.082 to -0.072)** | $4*10^{-204}$ |
| | | Above | 786 | -0.020 (-0.083 to 0.042) | 0.520 | -0.067 (-0.132 to -0.002) | 0.042 | -0.003 (-0.063 to 0.056) | 0.914 |

| | Cut-off | | Count | BMI | | ABSI | | HI | |
|---|---|---|---|---|---|---|---|---|---|
| | | | | $SD_{diff}$ (95% CI) | p-value | $SD_{diff}$ (95% CI) | p-value | $SD_{diff}$ (95% CI) | p-value |
| **WOMEN** *(continued)* | | | | | | | | | |
| **HbA1c** | 48 | Below | 128,377 | 0.114 (0.109 to 0.119)** | <1*$10^{-317}$ | 0.062 (0.057 to 0.067)** | 7*$10^{-131}$ | -0.044 (-0.049 to -0.039)** | 4*$10^{-68}$ |
| | | Above | 415 | -0.171 (-0.345 to 0.003) | 0.054 | 0.185 (-0.031 to 0.400) | 0.093 | -0.005 (-0.182 to 0.172) | 0.954 |
| **Neutrophils** | 2.0 | Below | 2398 | **-0.190 (-0.268 to -0.112)*** | **2*$10^{-6}$** | -0.092 (-0.153 to -0.031) | 0.003 | 0.034 (-0.027 to 0.096) | 0.268 |
| | | Above | 128,751 | 0.115 (0.110 to 0.120)** | <1*$10^{-317}$ | 0.057 (0.053 to 0.062)** | 7*$10^{-121}$ | -0.007 (-0.012 to -0.002) | 0.003 |
| **Neutrophils** | 7.5 | Below | 128,809 | 0.121 (0.115 to 0.126)** | <1*$10^{-317}$ | 0.057 (0.052 to 0.062)** | 1*$10^{-105}$ | -0.002 (-0.007 to 0.003) | 0.463 |
| | | Above | 2340 | -0.013 (-0.027 to 0.001) | 0.064 | 0.016 (0.001 to 0.031) | 0.035 | -0.013 (-0.027 to 0.001) | 0.068 |
| **CRP** | 8.0 | Below | 123,801 | 0.428 (0.424 to 0.433)** | <1*$10^{-317}$ | 0.099 (0.095 to 0.103)** | <1*$10^{-317}$ | -0.017 (-0.022 to -0.013)** | 1*$10^{-15}$ |
| | | Above | 5448 | **-0.060 (-0.070 to -0.050)**** | **2*$10^{-32}$** | 0.003 (-0.009 to 0.015) | 0.649 | 0.011 (0.000 to 0.021) | 0.056 |

**ABSI** – a body shape index; **ALP** – alkaline phosphatase (U/L); **ALT** – alanine aminotransferase (U/L); **ApoA1** – apolipoprotein A1 (g/L); **AST** – aspartate aminotransferase (U/L); **BMI** – body mass index; **Count** – number of participants with available biomarker measurements per subgroup; **CRP** – C-reactive protein (mg/L); **HbA1c** – haemoglobin A1c (glycated haemoglobin, mmol/mol); **HDL-C** – high-density lipoprotein cholesterol (mmol/L); **HI** – hip index; **Neutrophils** (*$10^9$/L).

**Cut-off** – see units above; **Below** – biomarker levels < cut-off; **Above** – biomarker levels ≥ cut-off.

**$SD_{diff}$ (95% CI)** – estimates for standard deviation difference (95% confidence interval) for each biomarker, per one SD increment of the corresponding anthropometric index, were obtained from multivariable linear regression models with each biomarker on a continuous scale (sex-specific z-scores, following log-transformation) as an outcome variable and BMI, ABSI, and HI on a continuous scale (sex-specific z-scores) as independent variables. All models were adjusted for height, age at enrolment, weight change within the last year preceding enrolment, smoking status, alcohol consumption, physical activity, Townsend deprivation index, region of the assessment centre, time of blood collection, fasting time, use of nonsteroidal anti-inflammatory drugs, paracetamol use, and in women also menopausal status, oral contraceptives use, hormone replacement therapy use, and age at the last live birth. Covariates are defined in Supplementary Methods.

* – p<0.0001 from Wald test for the individual term; ** – p<1*$10^{-6}$ (biomarkers with opposite direction of the association in the two groups and p<0.0002 for the small tail-end group are shown in bold).

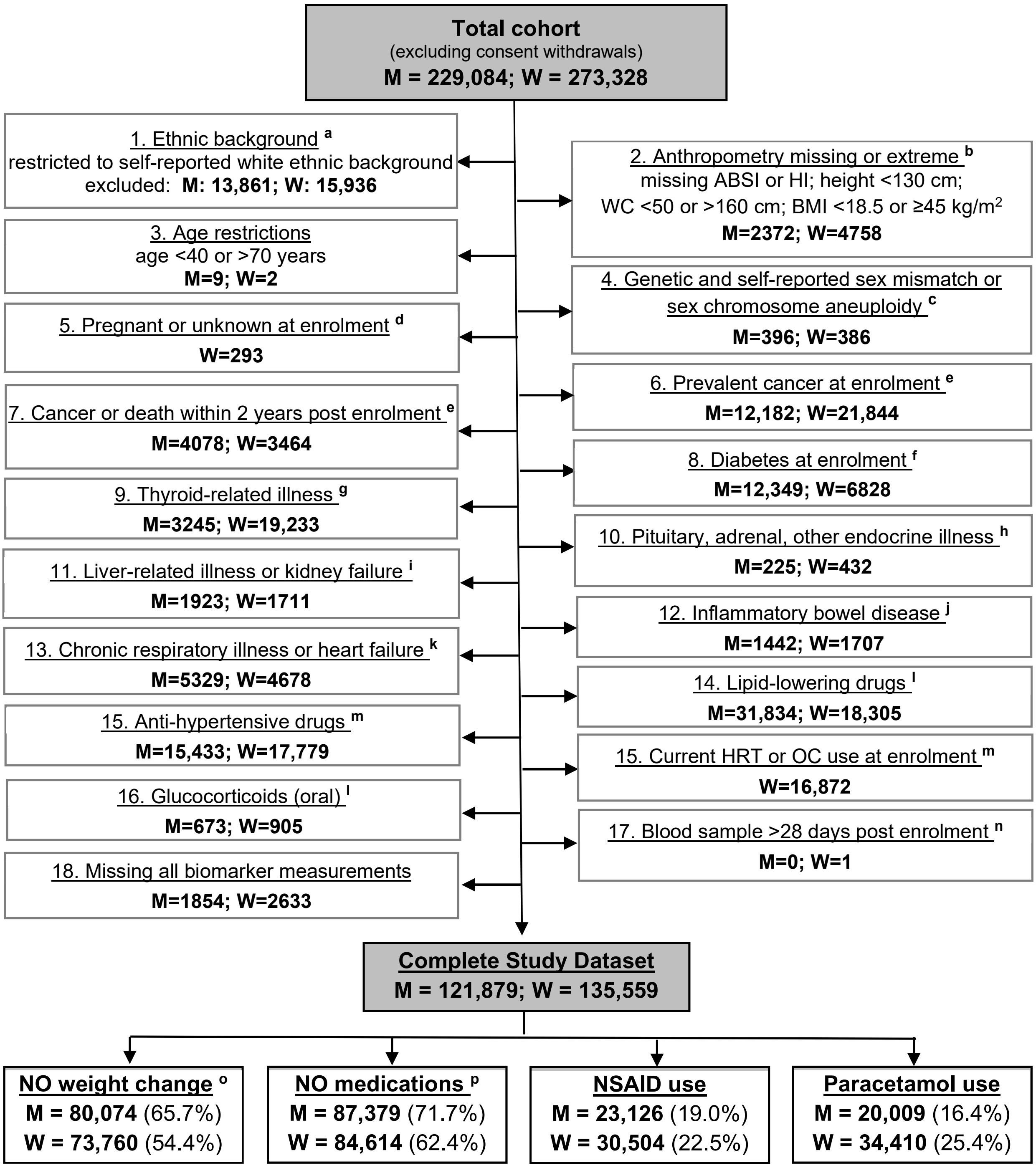


**Supplementary Figure S1 Flow diagram of UK Biobank participants in the study**

**ABSI** – a body shape index; **BMI** – body mass index; **HI** – hip index; **HRT** – hormone replacement therapy; **M** – number of men; **NSAID** – nonsteroidal anti-inflammatory drugs; **OC** – oral contraceptives; **W** – number of women; **WC** – waist circumference.

The exclusion criteria were applied sequentially in the displayed order, such that each excluded individual was counted only once. Specific fields used to define the exclusions are listed below:

[a] – Field [21000-0.0] "*Ethnic background*"; in the study were retained participants with codes: 1 "*White*", 1001 "*British*", 1002 "*Irish*", 1003 "*Any other white background*".

[b] – anthropometric measurements were obtained from Fields [48-0.0] "*Waist circumference*", Field [49-0.0] "*Hip circumference*", Field [50-0.0] "*Standing height*" and Field [21002-0.0] "*Weight*".

[c] – excluded were participants with code 1 for Field [22019-0.0] "*Sex chromosome aneuploidy*" OR with a mismatch between Field [22001-0.0] "*Genetic sex*" and Field [34-0.0] "*Sex (self-reported)*".

[d] – Field [3140-0.0] "*Pregnant"*; Answer: 1 "Yes", OR Answer 2: "Unsure", OR Missing.

[e] – prevalent and incident cancer cases and deaths were ascertained according to our previous publication [ref. 10].

[f] – definition based on a combination of self-reported diabetes status (see Supplementary Methods) and antidiabetic drug use (see list in Supplementary Table S1).

[g] – exclusions comprised thyroid-related non-cancer illness self-reported at enrolment from Fields [20002-0.0…33] "*Non-cancer illness code, self-reported*", including the following codes (as listed in UK Biobank Coding 6):

| | |
|---|---|
| 1224 | thyroid problem (not cancer) |
| 1225 | hyperthyroidism/thyrotoxicosis |
| 1226 | hypothyroidism/myxoedema |
| 1228 | thyroid radioablation therapy |
| 1428 | thyroiditis |
| 1522 | grave's disease |
| 1610 | thyroid goitre |

Note from UK Biobank: *If the participant was uncertain of the type of illness they had had, then they described it to the interviewer (a trained nurse) who attempted to place it within the coding tree. If the illness could not be located in the coding tree, then the interviewer entered a free-text description of it. These free-text descriptions were subsequently examined by a doctor and, where possible, matched to entries in the coding tree. Free-text descriptions which could not be matched with very high probability have been marked as "unclassifiable"*.

[h] – exclusions comprised pituitary, adrenal, and other endocrine non-cancer illness self-reported at enrolment (as in [g]) with codes:

| | |
|---|---|
| 1232 | disorder of adrenal gland |
| 1233 | adrenal tumour |
| 1234 | adrenocortical insufficiency/addison's disease |
| 1235 | hyperaldosteronism/conn's syndrome |
| 1236 | phaeochromocytoma |
| 1237 | disorder or pituitary gland |
| 1238 | pituitary adenoma/tumour |
| 1239 | cushings syndrome |
| 1429 | acromegaly |
| 1430 | hypopituitarism |
| 1431 | hyperprolactinaemia |
| 1432 | carcinoid syndrome/tumour |
| 1682 | benign insulinoma |

[i] – exclusions comprised liver-related non-cancer illness and kidney failure self-reported at enrolment (as in [g]) with codes:

1136 liver/biliary/pancreas problem
1155 hepatitis
1156 infective/viral hepatitis
1157 non-infective hepatitis
1158 liver failure/cirrhosis
1159 bile duct disease
1160 bile duct obstruction/ascending cholangitis
1192 renal/kidney failure
1193 renal failure requiring dialysis
1194 renal failure not requiring dialysis
1475 sclerosing cholangitis
1506 primary biliary cirrhosis
1507 haemochromatosis
1508 jaundice (unknown cause)
1578 hepatitis a, 1579 hepatitis b, 1580 hepatitis c, 1581 hepatitis d, 1582 hepatitis e
1604 alcoholic liver disease / alcoholic cirrhosis

[j] – exclusions comprised inflammatory bowel disease (non-cancer illness) self-reported at enrolment (as in [g]) with codes:

1461 inflammatory bowel disease
1462 crohns disease
1463 ulcerative colitis

[k] – exclusions comprised chronic respiratory non-cancer illness or heart failure self-reported at enrolment (as in [g]) with codes:

1076 heart failure/pulmonary odema
1112 chronic obstructive airways disease/copd
1113 emphysema/chronic bronchitis
1114 bronchiectasis
1115 interstitial lung disease
1120 asbestosis
1121 pulmonary fibrosis
1122 fibrosing alveolitis/unspecified alveolitis
1124 respiratory failure
1126 spontaneous pneumothorax/recurrent pneumothorax
1412 bronchitis
1472 emphysema
1496 alpha-1 antitrypsin deficiency

[l] – see list of lipid-lowering drugs or glucocorticoids for exclusion in Supplementary Table S1.

[m] – see definition of HRT, OC, and anti-hypertensive drugs use in Supplementary Methods.

[n] – calculated as the difference between the date of blood sample collection extracted from Field [3166-0.0] "*Time blood sample collected*" and the date of the baseline visit (at enrolment) Field [53-0.0] "*Date of attending assessment centre*".

[o] – excluding participants with weight loss or weight gain within the last year preceding enrolment (self-reported in Field [2306-0.0] "*Weight change compared with 1 year ago*").

[p] – excluding participants with NSAID or paracetamol use.

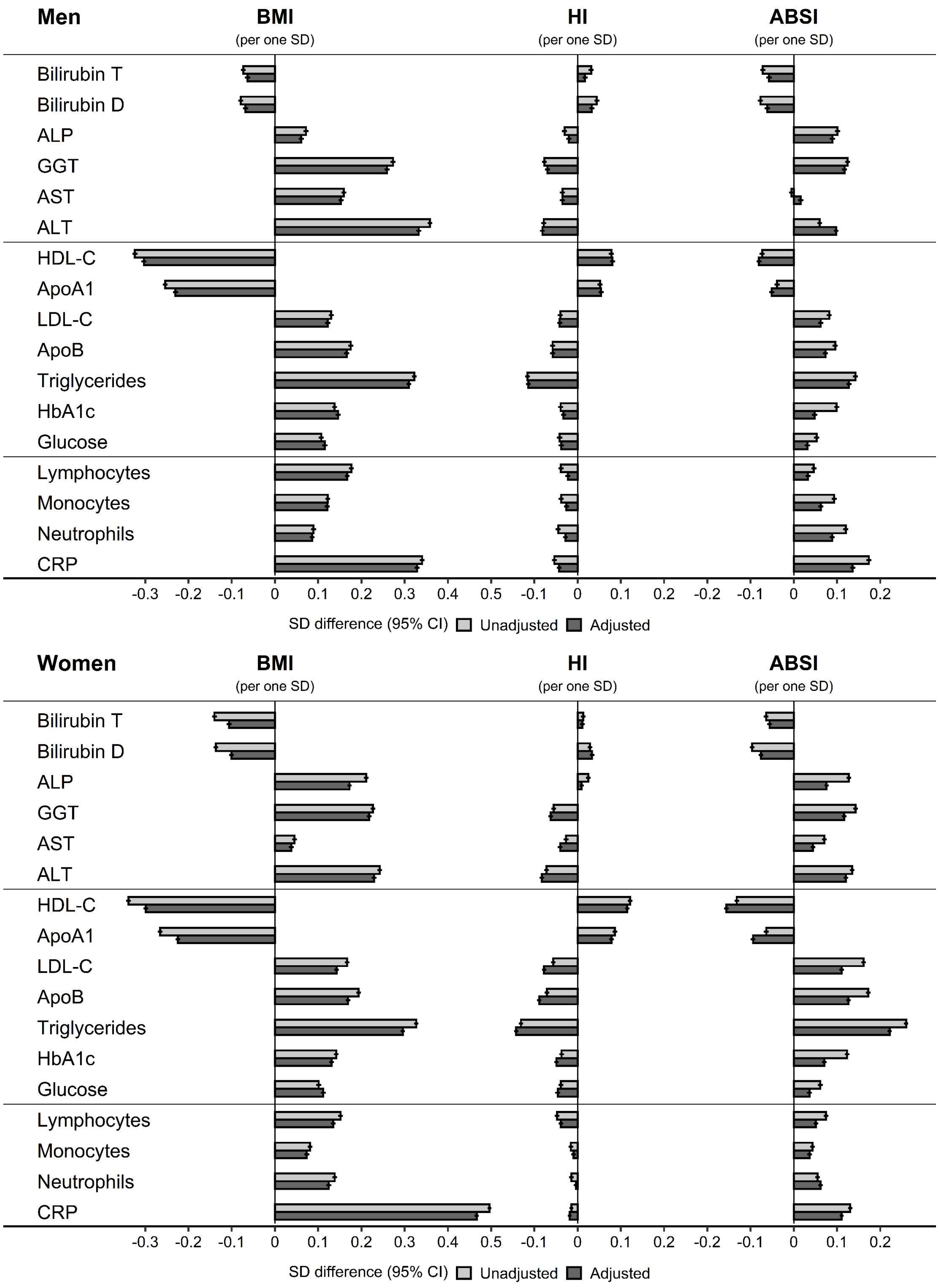


**Supplementary Figure S2 Associations of biomarkers with body size and body shape indices (continuous, unadjusted)**

**ABSI** – a body shape index; **ALP** – alkaline phosphatase; **ALT** – alanine aminotransferase; **ApoA1** – apolipoprotein A1; **ApoB** – apolipoprotein B; **AST** – aspartate aminotransferase; **Bilirubin D** – direct bilirubin; **Bilirubin T** – total bilirubin; **BMI** – body mass index; **CRP** – C-reactive protein; **GGT** – gamma-glutamyltransferase; **HbA1c** – haemoglobin A1c (glycated haemoglobin); **HDL-C** – high-density lipoprotein cholesterol; **HI** – hip index; **LDL-C** – low-density lipoprotein cholesterol; **SD** – standard deviation.

**SD difference (95% CI)** – unadjusted estimates for standard deviation difference (95% confidence interval) were obtained from multivariable linear regression models including each biomarker on a continuous scale (sex-specific z-scores, following log-transformation) as an outcome variable and BMI, ABSI, and HI on a continuous scale (sex-specific z-scores) as independent variables without any covariates. Adjusted models are included for comparison (estimates and covariates correspond to Figure 1). Adjustment variables included height, age at enrolment, weight change within the last year preceding enrolment, smoking status, alcohol consumption, physical activity, Townsend deprivation index, region of the assessment centre, time of blood collection, fasting time, nonsteroidal anti-inflammatory drug use, paracetamol use, and in women also menopausal status, oral contraceptives use, hormone replacement therapy use, and age at the last live birth.

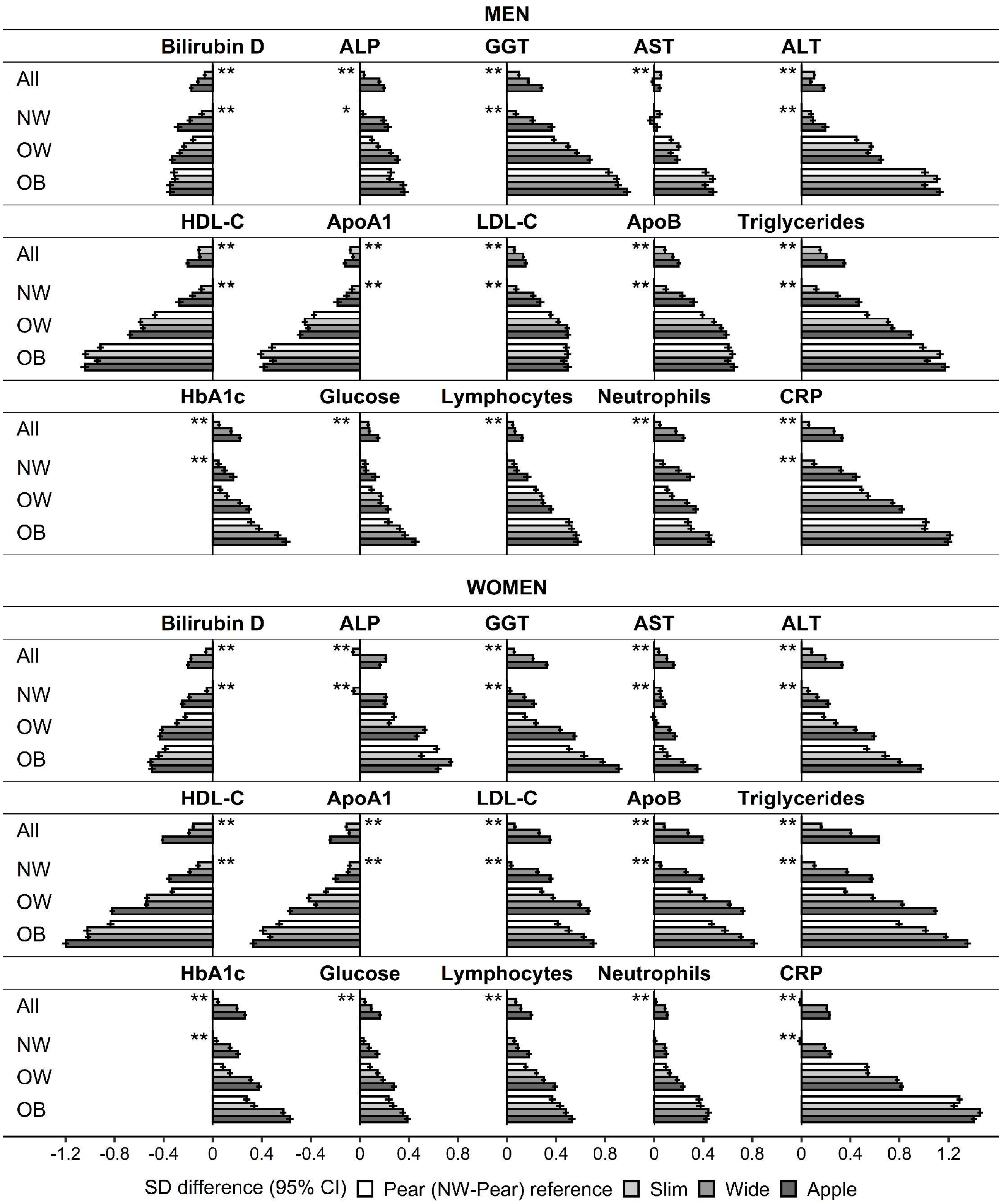


**Supplementary Figure S3 Associations of biomarkers with body shape phenotypes (categorical, unadjusted)**

**ABSI** – a body shape index (cut-offs ≥80 for men, ≥73 for women); **ALP** – alkaline phosphatase; **ALT** – alanine aminotransferase; **ApoA1** – apolipoprotein A1; **ApoB** – apolipoprotein B; **Apple** – large-ABSI-small-HI; **AST** – aspartate aminotransferase; **Bilirubin D** – direct bilirubin; **BMI** – body mass index; **CRP** – C-reactive protein; **GGT** – gamma-glutamyltransferase; **HbA1c** – haemoglobin

A1c (glycated haemoglobin); **HDL-C** – high-density lipoprotein cholesterol; **HI** – hip index (cut-offs ≥49 for men, ≥64 for women); **LDL-C** – low-density lipoprotein cholesterol; **NW** – normal weight (BMI≥18.5 to <25 kg/m$^2$); **OB** – obese (BMI≥30 to <45 kg/m$^2$); **OW** – overweight (BMI≥25 to <30 kg/m$^2$); **Pear** – small-ABSI-large-HI; **Slim** – small-ABSI-small-HI; **Wide** – large-ABSI-large-HI.

**SD difference (95% CI)** – estimates for standard deviation difference (95% confidence interval) were obtained from multivariable linear regression models including each biomarker on a continuous scale (sex-specific z-scores, following log-transformation) as an outcome variable and the following two combinations as independent variables: an ABSI-by-HI cross-classification and BMI categories (for ALL, reference "pear"), or an BMI-by-ABSI-by-HI cross-classification (for NW, OW, and OB, reference "pear"-NW). There was no further adjustment for covariates.

$\mathbf{p}_{\textbf{body shape}}$ (for ALL) – was obtained from a likelihood ratio test comparing a model including only BMI categories with a model additionally including an ABSI-by-HI cross-classification (evaluates the significance of body-shape overall).

$\mathbf{p}_{\textbf{interaction}}$ (for NW, OW, and OB) – was obtained from a likelihood ratio test comparing the additive model including the ABSI-by-HI cross-classification, BMI categories with the interaction model including the BMI-by-ABSI-by-HI cross-classification (evaluates heterogeneity by BMI).

* – $p<0.0001$; ** – $p<1*10^{-6}$

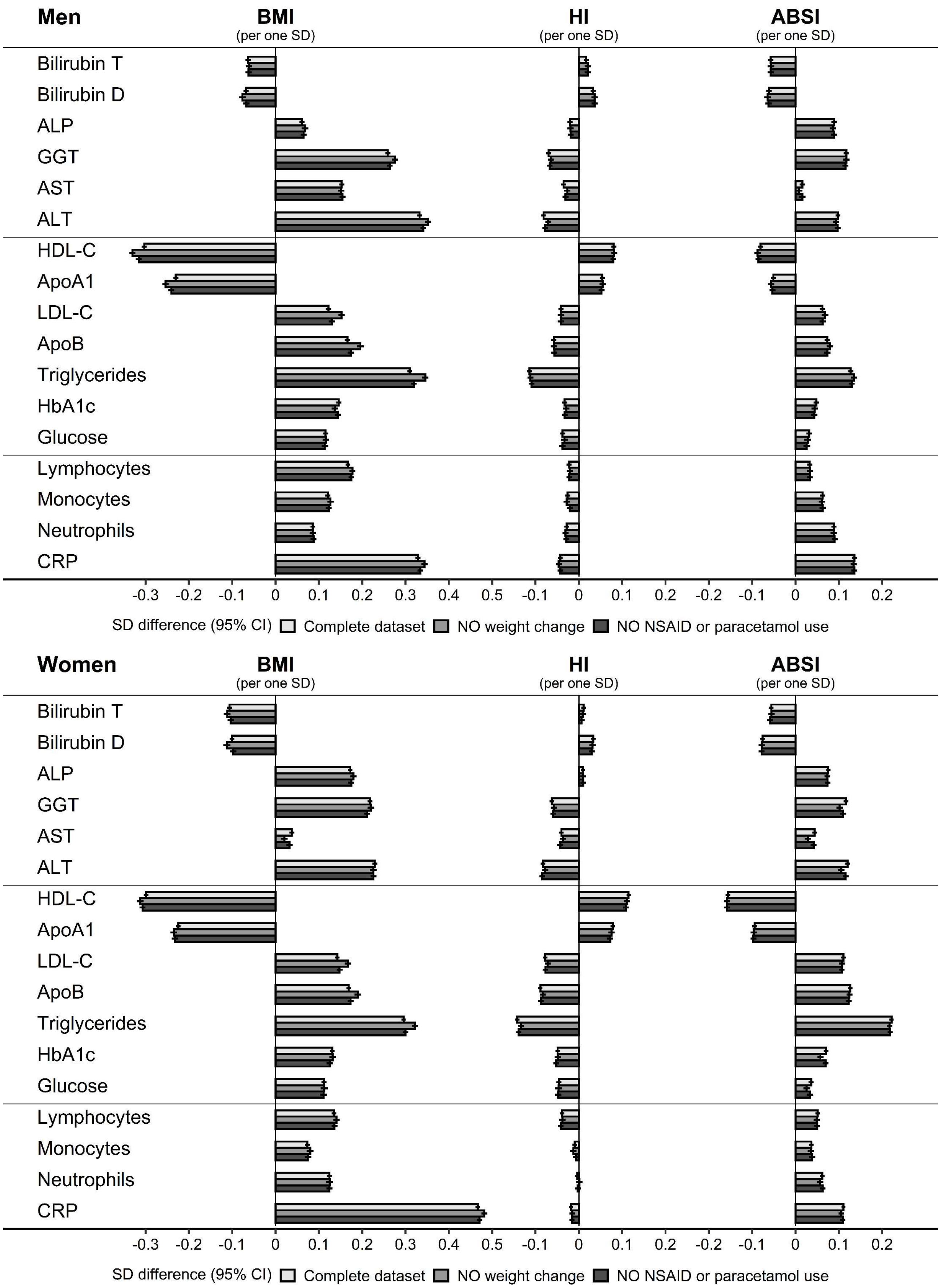


**Supplementary Figure S4A Associations of biomarkers with body size and body shape indices: excluding weight loss or medication use**

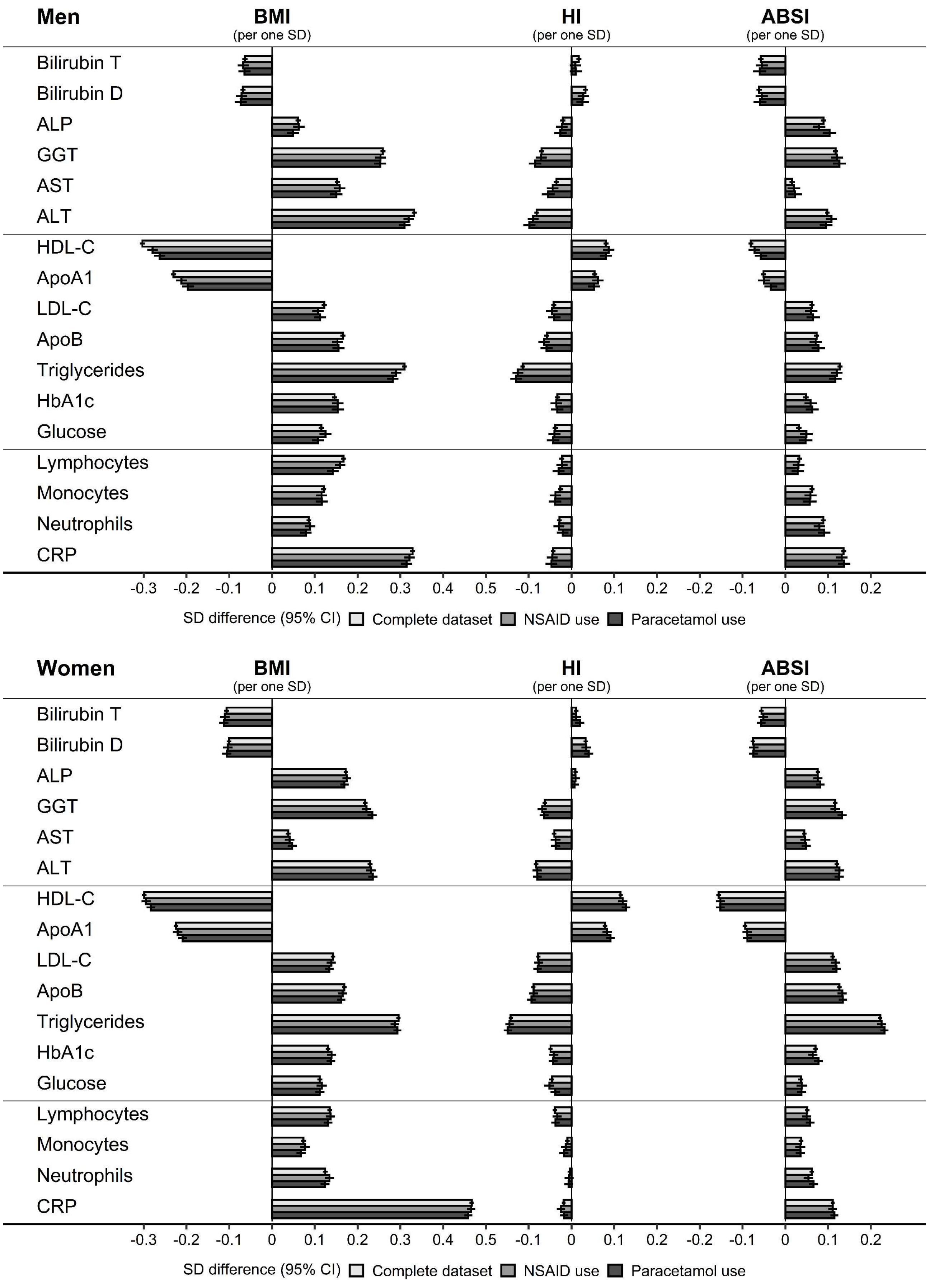


**Supplementary Figure S4B Associations of biomarkers with body size and body shape indices: subgroups with medication use**

**ABSI** – a body shape index; **ALP** – alkaline phosphatase; **ALT** – alanine aminotransferase; **ApoA1** – apolipoprotein A1; **ApoB** – apolipoprotein B; **AST** – aspartate aminotransferase; **Bilirubin D** – direct bilirubin; **Bilirubin T** – total bilirubin; **BMI** – body mass index; **CRP** – C-reactive protein; **GGT** – gamma-glutamyltransferase; **HbA1c** – haemoglobin A1c (glycated haemoglobin); **HDL-C** – high-density lipoprotein cholesterol; **HI** – hip index; **LDL-C** – low-density lipoprotein cholesterol; **NSAID** – nonsteroidal anti-inflammatory drugs; **SD** – standard deviation; **NO weight change** – no weight loss or weight gain within the last year preceding enrolment (self-reported).

**SD difference (95% CI)** – estimates for standard deviation difference (95% confidence interval) were obtained from multivariable linear regression models including each biomarker on a continuous scale (sex-specific z-scores, following log-transformation) as an outcome variable and BMI, ABSI, and HI on a continuous scale (sex-specific z-scores) as independent variables. All models were adjusted for height, age at enrolment, weight change within the last year preceding enrolment (except for the subgroup with no weight change), smoking status, alcohol consumption, physical activity, Townsend deprivation index, region of the assessment centre, time of blood collection, fasting time, NSAID use (for the subgroups with no weight change and paracetamol use), paracetamol use (for the subgroups with no weight change and NSAID use), and in women also menopausal status, oral contraceptives use, hormone replacement therapy use, and age at the last live birth. Covariates are defined in Supplementary Methods. Participant counts per subgroup are shown in Supplementary Figure S1 and by subgroup and body shape phenotype in Table 1.

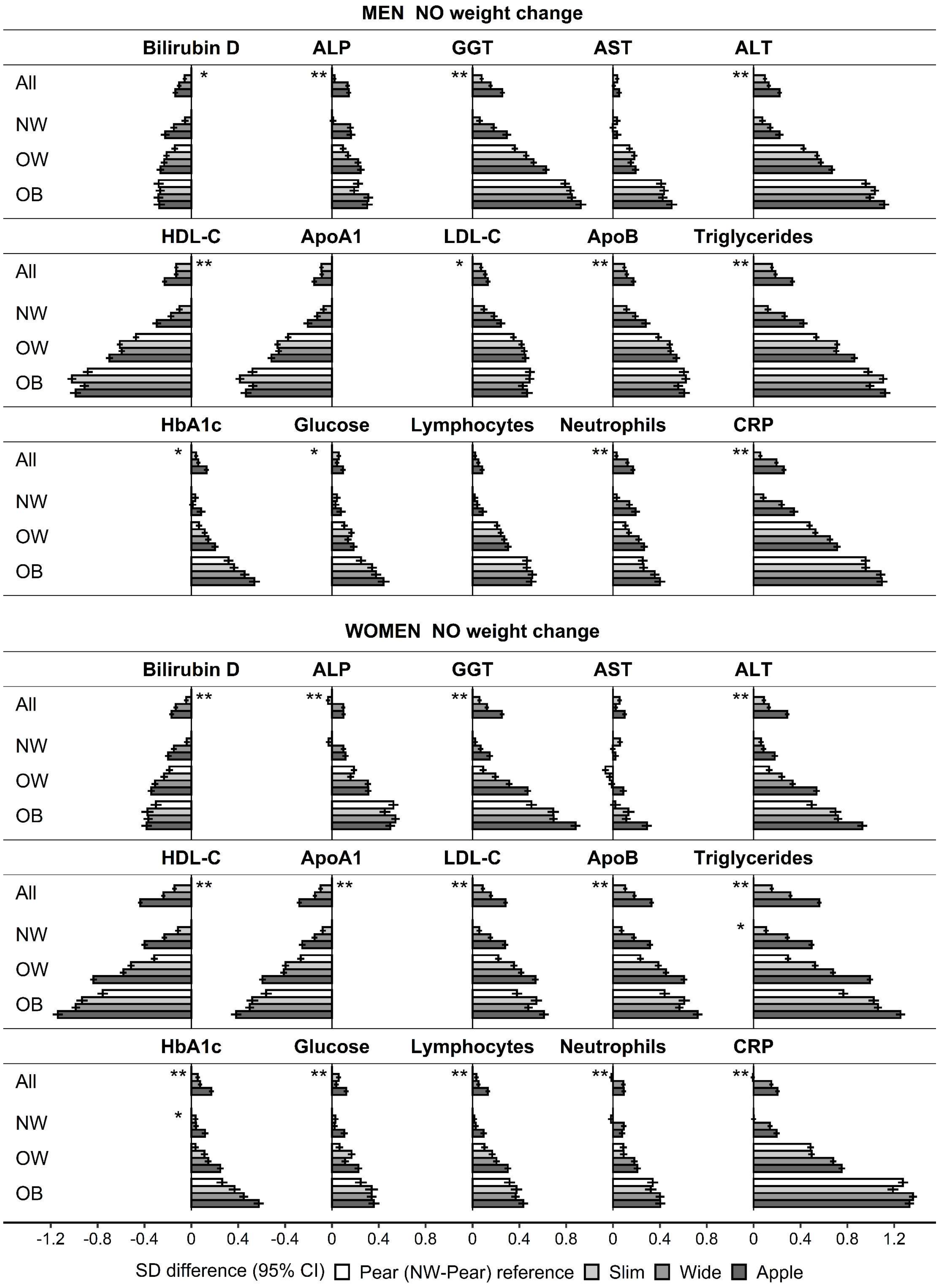


**Supplementary Figure S5A Associations of biomarkers with body shape phenotypes: subgroup without recent weight change**

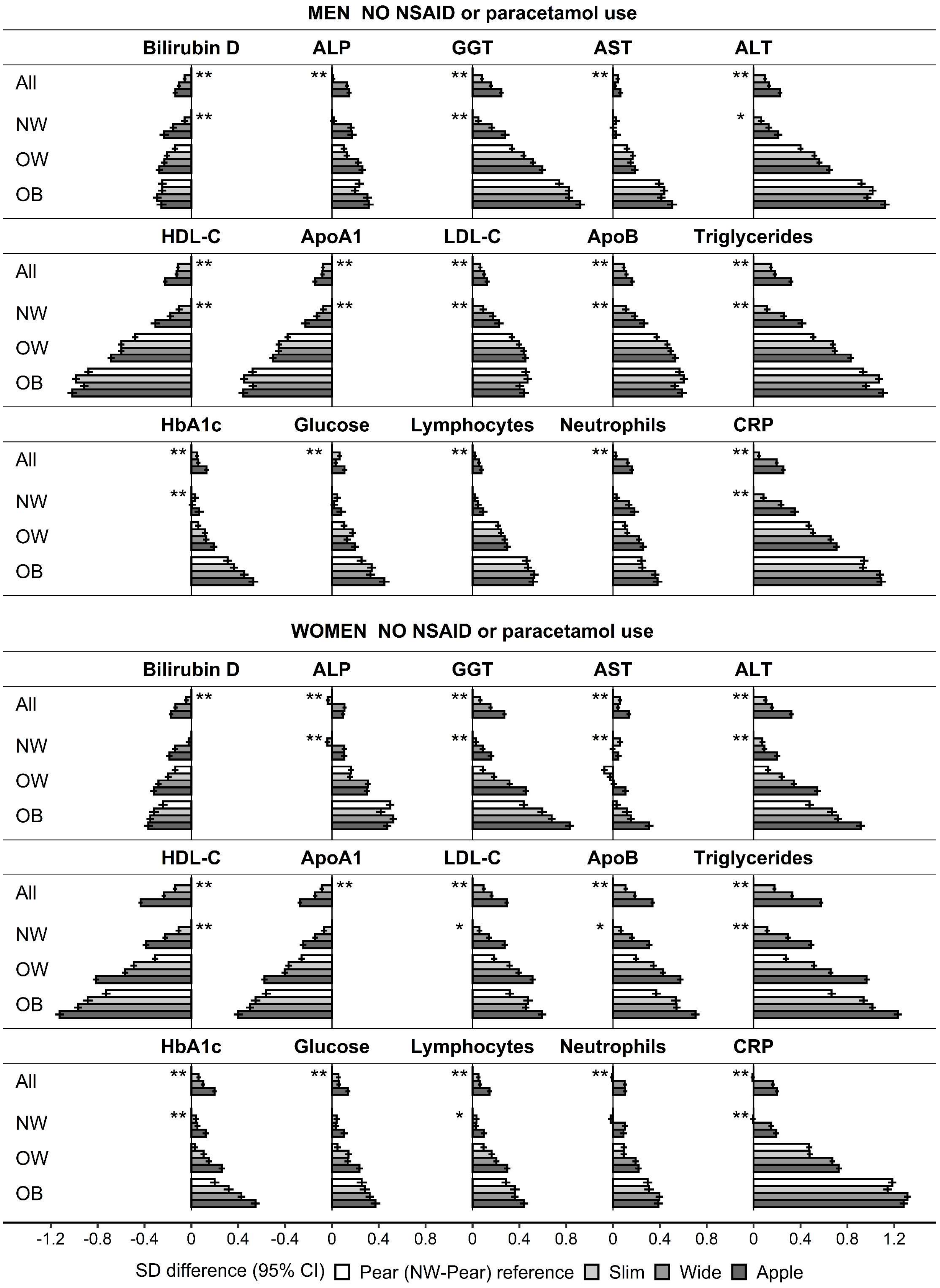


**Supplementary Figure S5B Associations of biomarkers with body shape phenotypes: subgroup without NSAID or paracetamol use**

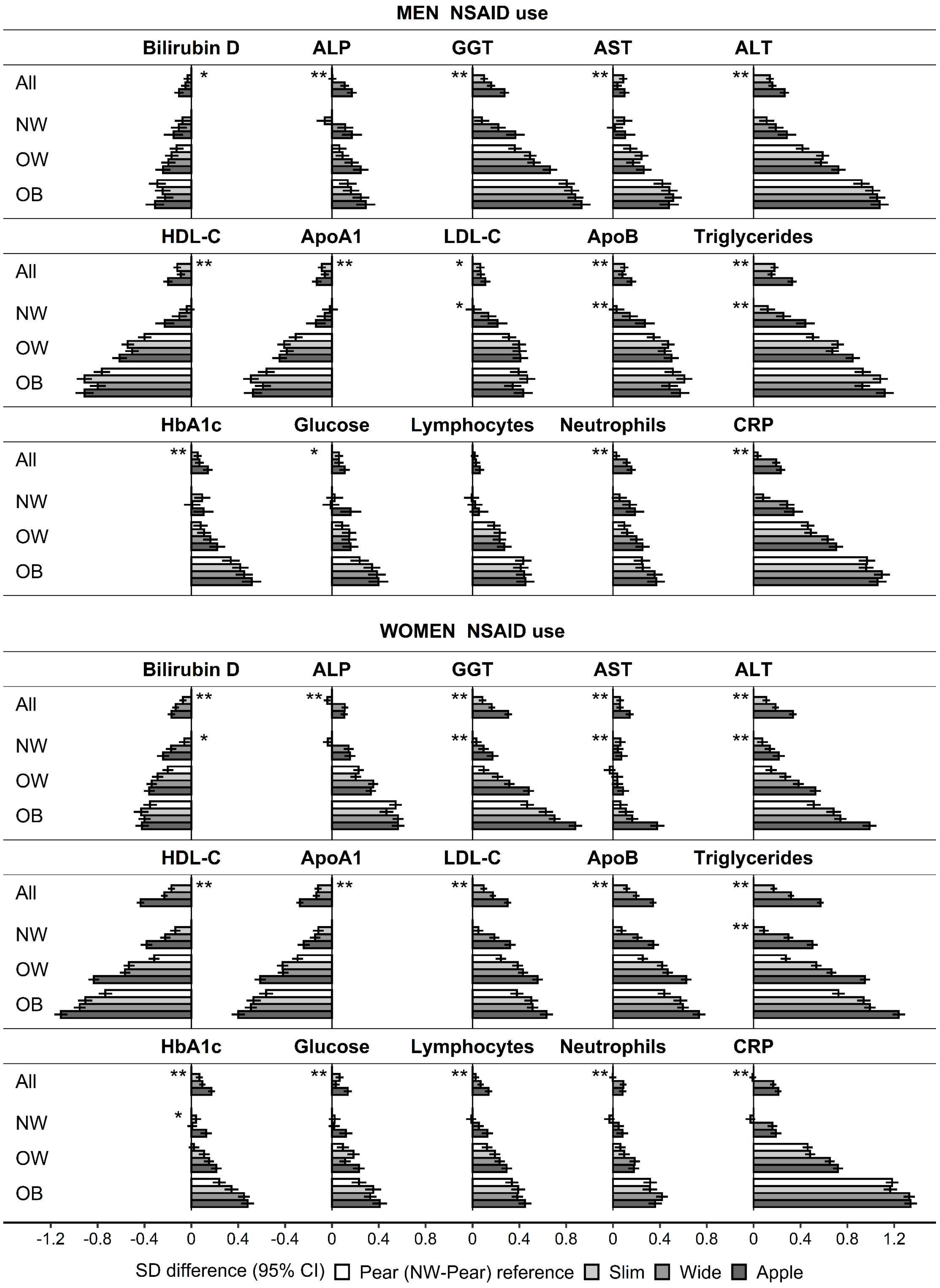


**Supplementary Figure S5C Associations of biomarkers with body shape phenotypes: subgroup with NSAID use**

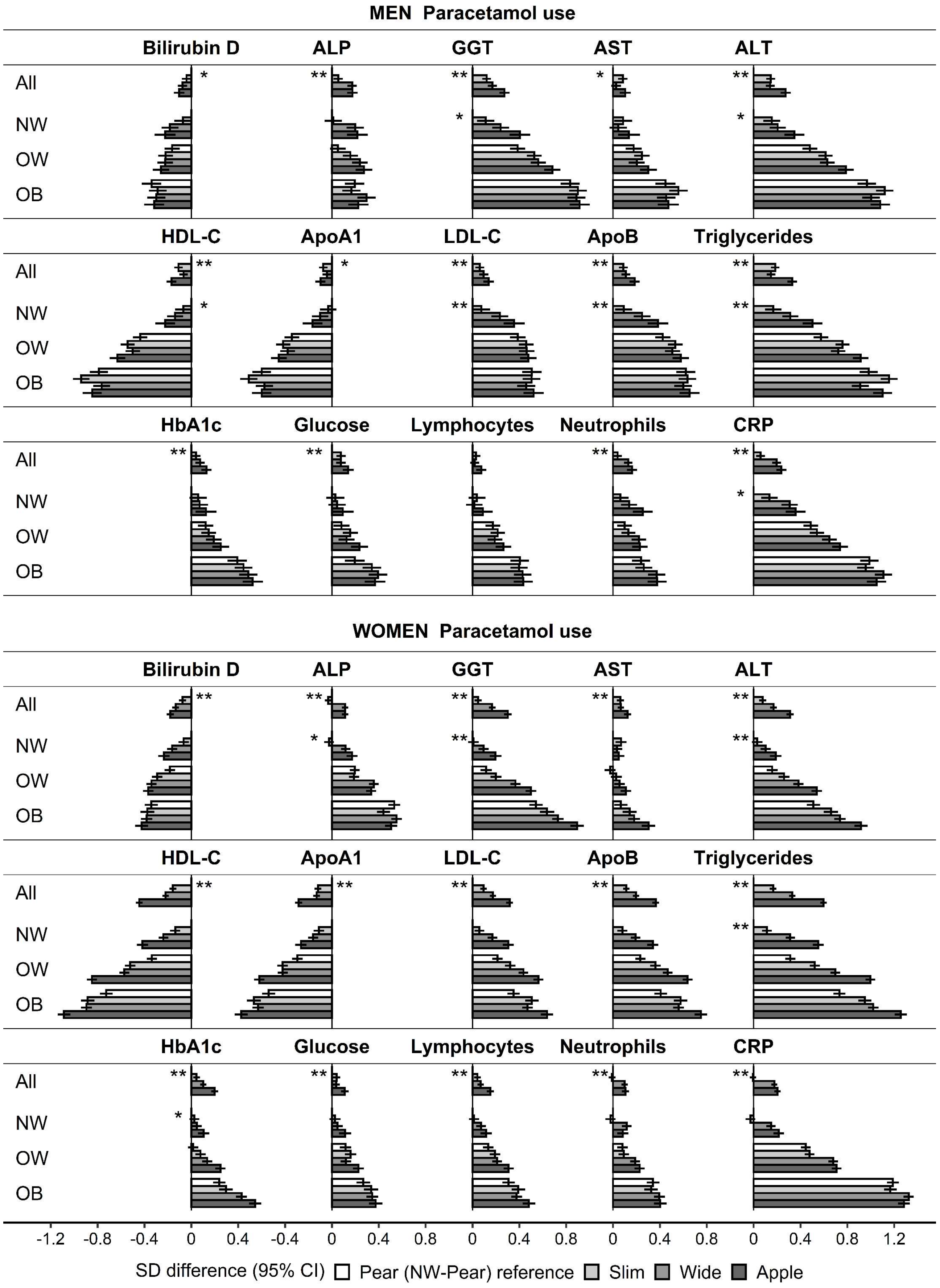


**Supplementary Figure S5D Associations of biomarkers with body shape phenotypes: subgroup with paracetamol use**

**ABSI** – a body shape index (cut-offs ≥80 for men, ≥73 for women); **ALP** – alkaline phosphatase; **ALT** – alanine aminotransferase; **ApoA1** – apolipoprotein A1; **ApoB** – apolipoprotein B; **Apple** – large-ABSI-small-HI; **AST** – aspartate aminotransferase; **Bilirubin D** – direct bilirubin; **BMI** – body mass index; **CRP** – C-reactive protein; **GGT** – gamma-glutamyltransferase; **HbA1c** – haemoglobin A1c (glycated haemoglobin); **HDL-C** – high-density lipoprotein cholesterol; **HI** – hip index (cut-offs ≥49 for men, ≥64 for women); **LDL-C** – low-density lipoprotein cholesterol; **NSAID** – nonsteroidal anti-inflammatory drugs; **NW** – normal weight (BMI≥18.5 to <25 kg/m$^2$); **OB** – obese (BMI≥30 to <45 kg/m$^2$); **OW** – overweight (BMI≥25 to <30 kg/m$^2$); **Pear** – small-ABSI-large-HI; **Slim** – small-ABSI-small-HI; **NO weight change** – no weight loss or weight gain within the last year preceding enrolment (self-reported); **Wide** – large-ABSI-large-HI.

**SD difference (95% CI)** – estimates for standard deviation difference (95% confidence interval) were obtained from multivariable linear regression models including each biomarker on a continuous scale (sex-specific z-scores, following log-transformation) as an outcome variable and the following two combinations as independent variables: an ABSI-by-HI cross-classification and BMI categories (for ALL, reference “pear”), or an BMI-by-ABSI-by-HI cross-classification (for NW, OW, and OB, reference “pear”-NW). All models were adjusted for height, age at enrolment, weight change within the last year preceding enrolment (except for the subgroup with no weight change), smoking status, alcohol consumption, physical activity, Townsend deprivation index, region of the assessment centre, time of blood collection, fasting time, NSAID use (for the subgroups with no weight change and paracetamol use), paracetamol use (for the subgroups with no weight change and NSAID use), and in women also menopausal status, oral contraceptives use, hormone replacement therapy use, and age at the last live birth. Covariates are defined in Supplementary Methods. Participant counts per subgroup are shown in Supplementary Figure S1 and by subgroup and body shape phenotype in Table 1.

**p** $_{\textbf{body shape}}$ (for ALL) – was obtained from a likelihood ratio test comparing a model including only BMI categories and covariates with a model additionally including an ABSI-by-HI cross-classification (evaluates the significance of body-shape overall).

**p** $_{\textbf{interaction}}$ (for NW, OW, and OB) – was obtained from a likelihood ratio test comparing the additive model including the ABSI-by-HI cross-classification, BMI categories, and covariates with the interaction model including the BMI-by-ABSI-by-HI cross-classification and covariates (evaluates heterogeneity by BMI).

* – p<0.0001; ** – $p<1*10^{-6}$

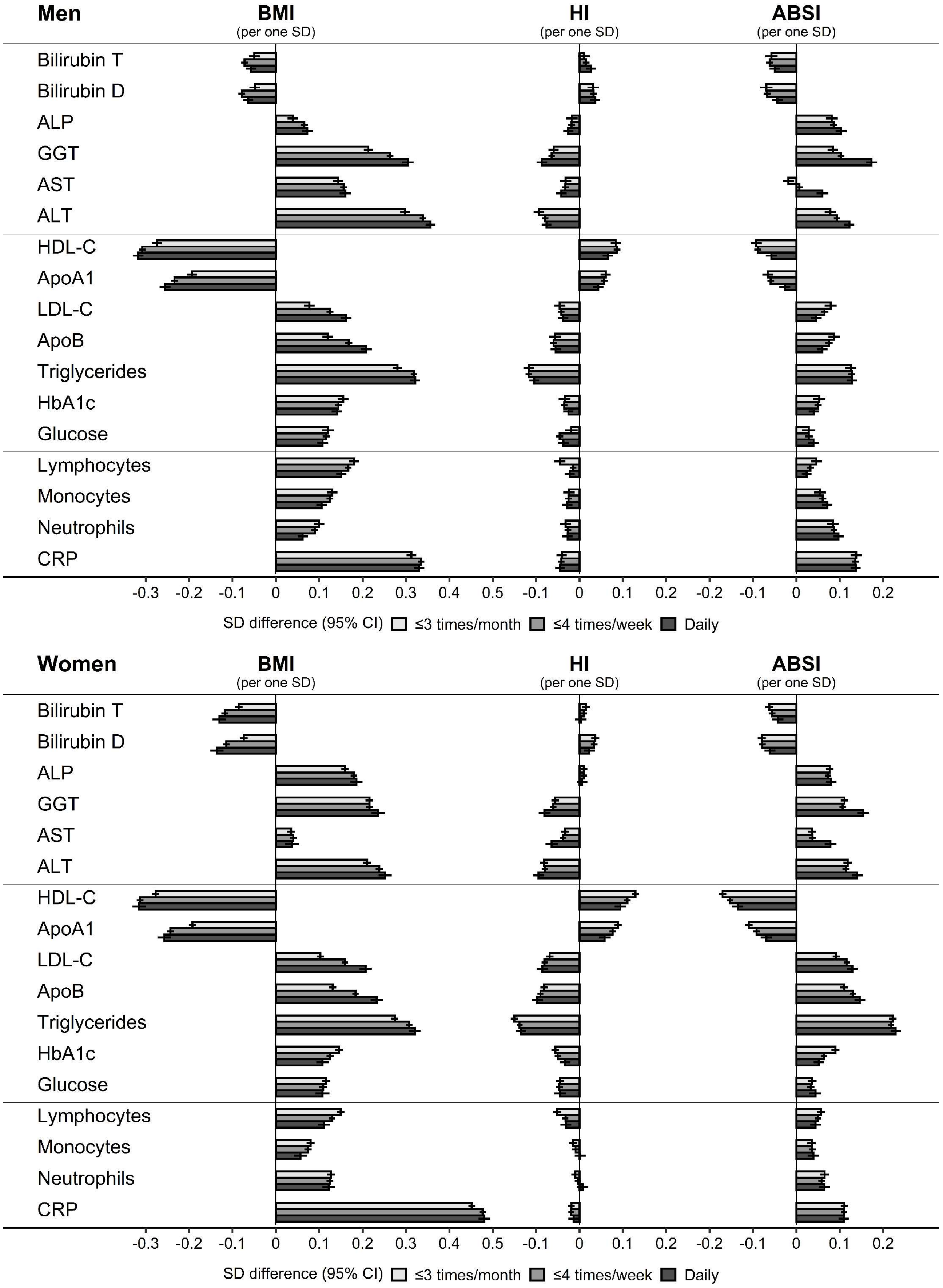


**Supplementary Figure S6 Associations of biomarkers with body size and body shape indices: subgroups according to alcohol consumption**

**ABSI** – a body shape index; **ALP** – alkaline phosphatase; **ALT** – alanine aminotransferase; **ApoA1** – apolipoprotein A1; **ApoB** – apolipoprotein B; **AST** – aspartate aminotransferase; **Bilirubin D** – direct bilirubin; **Bilirubin T** – total bilirubin; **BMI** – body mass index; **CRP** – C-reactive protein; **GGT** – gamma-glutamyltransferase; **HbA1c** – haemoglobin A1c (glycated haemoglobin); **HDL-C** – high-density lipoprotein cholesterol; **HI** – hip index; **LDL-C** – low-density lipoprotein cholesterol; **SD** – standard deviation.

**SD difference (95% CI)** – estimates for standard deviation difference (95% confidence interval) were obtained from multivariable linear regression models including each biomarker on a continuous scale (sex-specific z-scores, following log-transformation) as an outcome variable and BMI, ABSI, and HI on a continuous scale (sex-specific z-scores) as independent variables. All models were adjusted for height, age at enrolment, weight change within the last year preceding enrolment, smoking status, physical activity, Townsend deprivation index, region of the assessment centre, time of blood collection, fasting time, NSAID use, paracetamol use, and in women also menopausal status, oral contraceptives use, hormone replacement therapy use, and age at the last live birth. Covariates are defined in Supplementary Methods.

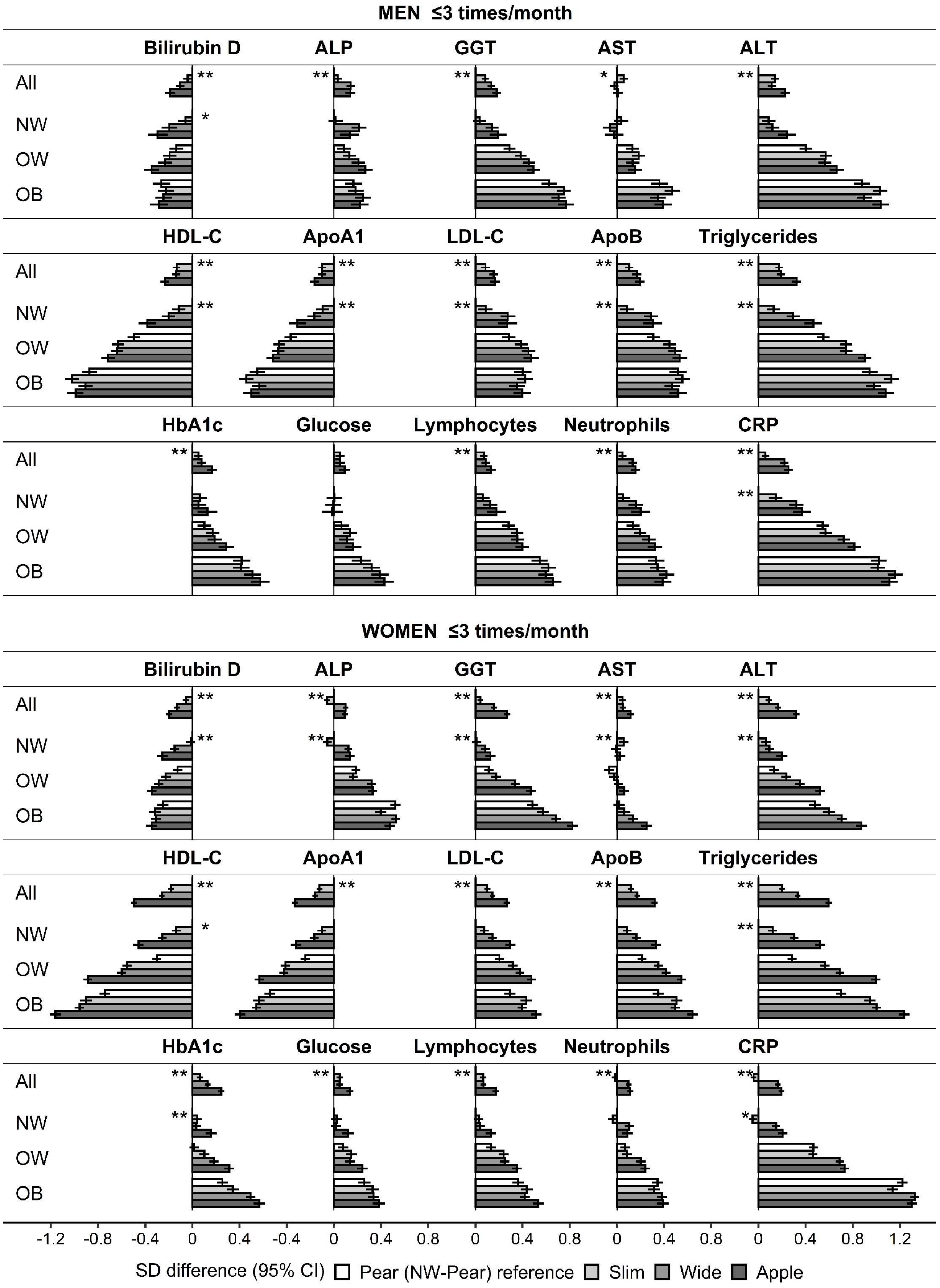


**Supplementary Figure S7A Associations of biomarkers with body shape phenotypes: up to 3 times a month alcohol consumption**

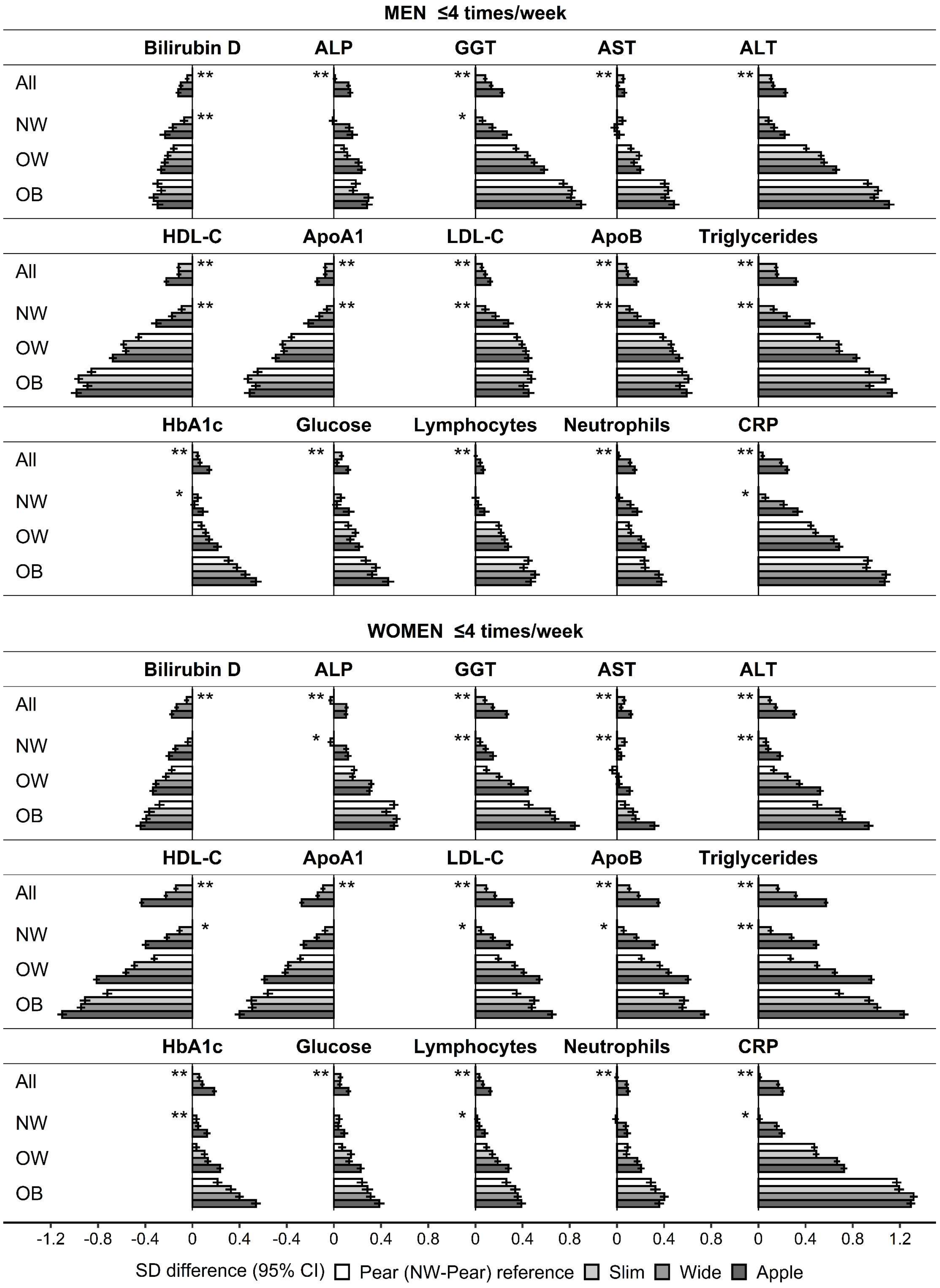


**Supplementary Figure S7B Associations of biomarkers with body shape phenotypes: up to four times a week alcohol consumption**

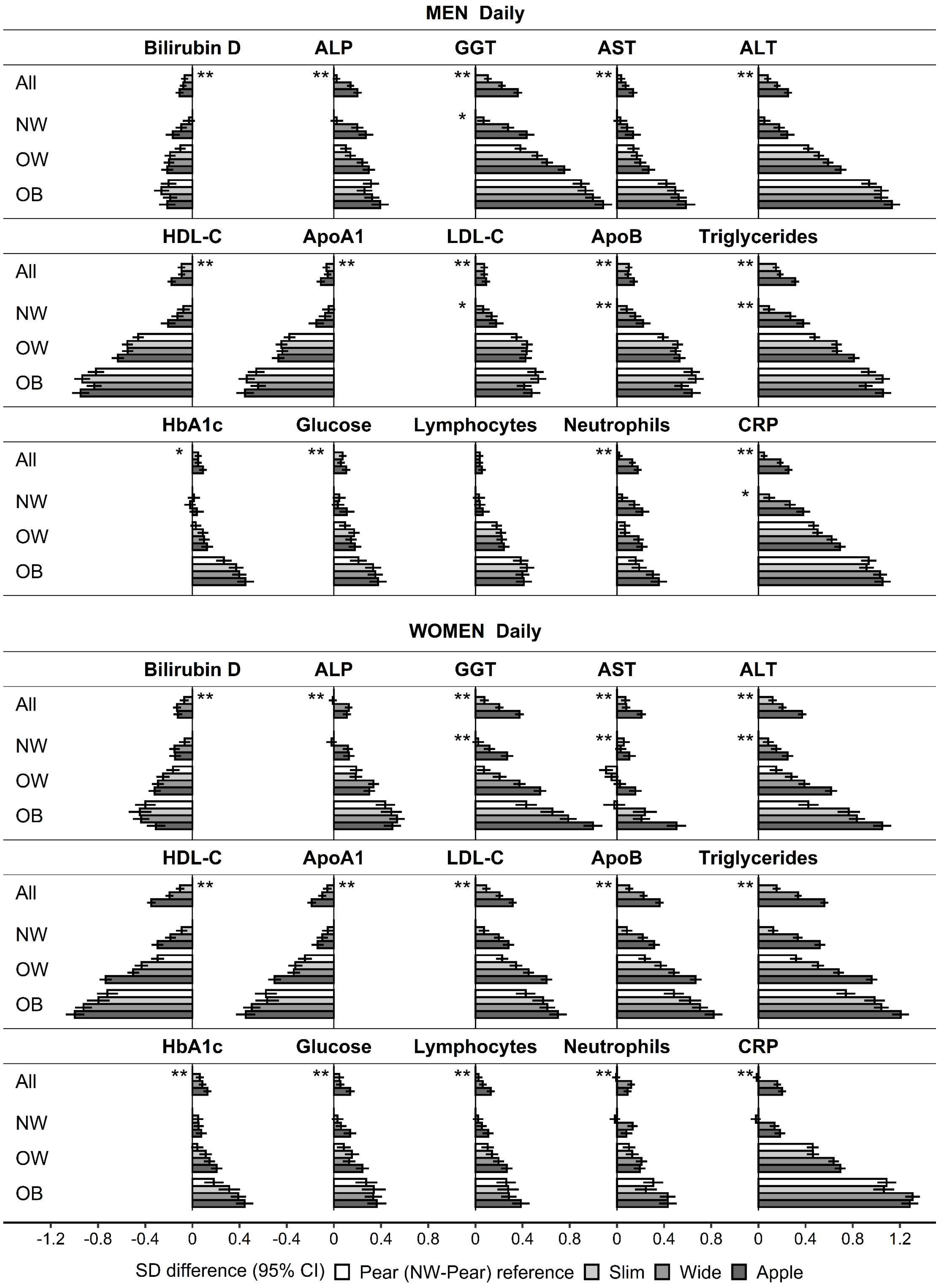


**Supplementary Figure S7C Associations of biomarkers with body shape phenotypes: daily alcohol consumption**

**ABSI** – a body shape index (cut-offs ≥80 for men, ≥73 for women); **ALP** – alkaline phosphatase; **ALT** – alanine aminotransferase; **ApoA1** – apolipoprotein A1; **ApoB** – apolipoprotein B; **Apple** – large-ABSI-small-HI; **AST** – aspartate aminotransferase; **Bilirubin D** – direct bilirubin; **BMI** – body mass index; **CRP** – C-reactive protein; **GGT** – gamma-glutamyltransferase; **HbA1c** – haemoglobin A1c (glycated haemoglobin); **HDL-C** – high-density lipoprotein cholesterol; **HI** – hip index (cut-offs ≥49 for men, ≥64 for women); **LDL-C** – low-density lipoprotein cholesterol; **NSAID** – nonsteroidal anti-inflammatory drugs; **NW** – normal weight (BMI≥18.5 to <25 kg/m$^2$); **OB** – obese (BMI≥30 to <45 kg/m$^2$); **OW** – overweight (BMI≥25 to <30 kg/m$^2$); **Pear** – small-ABSI-large-HI; **Slim** – small-ABSI-small-HI; **Wide** – large-ABSI-large-HI.

**SD difference (95% CI)** – estimates for standard deviation difference (95% confidence interval) were obtained from multivariable linear regression models including each biomarker on a continuous scale (sex-specific z-scores, following log-transformation) as an outcome variable and the following two combinations as independent variables: an ABSI-by-HI cross-classification and BMI categories (for ALL, reference “pear”), or an BMI-by-ABSI-by-HI cross-classification (for NW, OW, and OB, reference “pear”-NW). All models were adjusted for height, age at enrolment, weight change within the last year preceding enrolment, smoking status, physical activity, Townsend deprivation index, region of the assessment centre, time of blood collection, fasting time, use of nonsteroidal anti-inflammatory drugs, paracetamol use, and in women also menopausal status, oral contraceptives use, hormone replacement therapy use, and age at the last live birth. Covariates are defined in Supplementary Methods. Participant counts per subgroup and body shape phenotype are shown in Supplementary Table S3 (missing values were assigned the sex-specific median category, ≤4 times/week for all).

**p** $_{\textbf{body shape}}$ (for ALL) – was obtained from a likelihood ratio test comparing a model including only BMI categories and covariates with a model additionally including an ABSI-by-HI cross-classification (evaluates the significance of body-shape overall).

**p** $_{\textbf{interaction}}$ (for NW, OW, and OB) – was obtained from a likelihood ratio test comparing the additive model including the ABSI-by-HI cross-classification, BMI categories, and covariates with the interaction model including the BMI-by-ABSI-by-HI cross-classification and covariates (evaluates heterogeneity by BMI).

* – p<0.0001; ** – $p<1*10^{-6}$

## References

The numbers of the cited references correspond to the main document.

10. Christakoudi, S., Tsilidis, K. K., Evangelou, E., Riboli, E. A Body Shape Index (ABSI), hip index and risk of cancer in the UK Biobank cohort. *Cancer Med* **10,** 5614-5628; 10.1002/CAM4.4097 (2021).

14. Christakoudi, S., Tsilidis, K. K., Evangelou, E. & Riboli, E. Association of body-shape phenotypes with imaging measures of body composition in the UK Biobank cohort: relevance to colon cancer risk. *BMC Cancer* **21**, 1160; 10.1186/s12885-021-08820-6 (2021).
